# *BRAVO* self-confined expression through WOX5 in the *Arabidopsis* root stem-cell niche

**DOI:** 10.1242/dev.200510

**Published:** 2022-07-28

**Authors:** Josep Mercadal, Isabel Betegón-Putze, Nadja Bosch, Ana I. Caño-Delgado, Marta Ibañes

**Affiliations:** 1 Departament de Física de la Matèria Condensada, Facultat de Física, Universitat de Barcelona, 08028 Barcelona, Spain; 2 Universitat de Barcelona Institute of Complex Systems (UBICS), Universitat de Barcelona, 08028 Barcelona, Spain; 3 Department of Molecular Genetics, Centre for Research in Agricultural Genomics (CRAG), CSIC-IRTA-UAB-UB, Campus UAB (Cerdanyola del Vallès), 08193 Barcelona, Spain

**Keywords:** BRAVO, WOX5, Root, Stem-cell niche, Diffusion, Sequestration

## Abstract

In animals and plants, stem-cell niches are local microenvironments that are tightly regulated to preserve their unique identity while communicating with adjacent cells that will give rise to specialized cell types. In the primary root of *Arabidopsis thaliana*, two transcription factors, BRAVO and WOX5, among others, are expressed in the stem-cell niche. Intriguingly, BRAVO, a repressor of quiescent center divisions, confines its own gene expression to the stem-cell niche, as evidenced in a *bravo* mutant background. Here, we propose through mathematical modeling that BRAVO confines its own expression domain to the stem-cell niche by attenuating a WOX5-dependent diffusible activator of BRAVO. This negative feedback drives WOX5 activity to be spatially restricted as well. The results show that WOX5 diffusion and sequestration by binding to BRAVO are sufficient to drive the experimentally observed confined *BRAVO* expression at the stem-cell niche. We propose that the attenuation of a diffusible activator can be a general mechanism acting at other stem-cell niches to spatially confine genetic activity to a small region while maintaining signaling within them and with the surrounding cells.

## Abstract (precis)

**Summary:** Mathematical modeling of transcription factors expressed in the *Arabidopsis* root stem-cell niche shows that immobilization of WOX5 by BRAVO sequestration is sufficient to drive spatially self-confined *BRAVO* expression.

## INTRODUCTION

Both in animals and plants, stem cells are maintained in tightly regulated microenvironments called stem-cell niches (SCNs), in which they remain in an undifferentiated state and provide a continuous flux of precursors of more specialized cells that sustain growth and replace old or damaged tissues (Sablowski, 2007). SCNs usually consist of a few stem cells that are maintained by short-range signals produced by localized sources or ‘organizing centers’, groups of cells that sustain neighboring cells in a stem-cell state (Scadden, 2006; Spradling et al., 2001). As stem-cell daughters are placed outside the reach of these signals, they begin to differentiate, giving rise to more specialized cell types (Sablowski, 2004). In animals, diffusible ligands are common signals that preserve stem cells (Chen and McKearin, 2003; Han et al., 2008; Xie and Spradling, 1998; Zhang and Kalderon, 2001). Overproduction of these signals can drive an increase in the number of stem cells, resulting in enlarged niches and often leading to malfunctioning of the surrounding tissue or even whole organs (Spradling et al., 2001). Knowing the origin and function of these signals is therefore essential to understand the role of stem cells in the processes underlying organism development and sustenance.

In the model plant *Arabidopsis thaliana*, highly mobile hormones, such as auxin (Aida et al., 2004; Ding and Friml, 2010; Müller and Sheen, 2008), as well as short-range transcription factors like WUSCHEL and WUSCHEL-RELATED HOMEOBOX 5 (WOX5) (Sarkar et al., 2007; Yadav et al., 2011) are involved in specifying stem-cell niches. In the root apical meristem of *Arabidopsis*, the SCN lies at the tip of the root, a location known to be established by positional information conferred by the overlapping of the SCARECROW and SHORTROOT transcription factors, together with the activation of PLETHORA genes by the hormone auxin, the levels of which peak at the position where the SCN is established (Kidner et al., 2000; Shimotohno et al., 2018; van den Berg et al., 1995). This positional signaling allows for the necessary plasticity to establish a new niche when it has been destroyed or damaged, by a continual supply and renewal of stem cells at the same location (Sena et al., 2009).

**Fig. 1.**
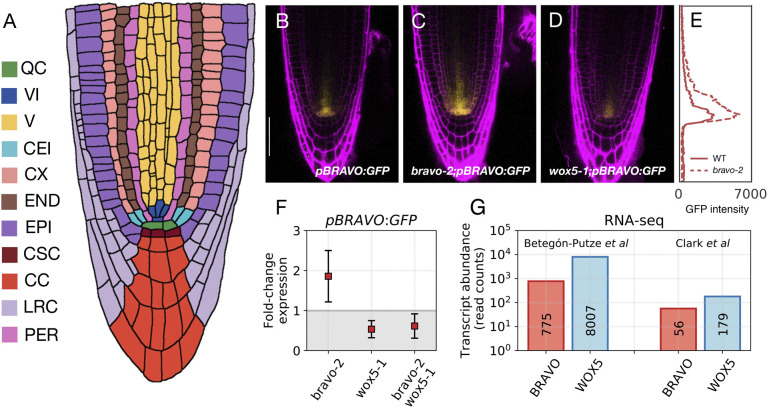
**Spatial confinement of *BRAVO* promoter expression.** (A) Cartoon of the root apical meristem depicting its organization in cell-types: quiescent center, QC; vascular initials, VI; vascular cells, V; cortex/endodermis initials, CEI; cortex, CX; endodermis, END; epidermis, EPI; columella stem cells, CSC; columella cells, CC; lateral root cap, LRC; pericycle, PER. (B-D) Confocal images of propidium iodide-stained (magenta) 6-day-old roots. GFP-tagged protein expression is shown in yellow. Activity of the *BRAVO* promoter in WT (B), and in *bravo-2* (C) and *wox5-1* (D) loss-of-function backgrounds is shown. Scale bar: 50 μm. The promoter activity of *BRAVO* expands its domain in the *bravo-2* mutant but not in the *wox5-1* mutant. Data and images in B-D are taken from experiments conducted for Betegón-Putze et al. (2021). (E) Total GFP fluorescence intensity along the vertical axis for the images shown in B (WT, solid line in E) and C (*bravo-2* mutant, dashed line in E), indicated as the sum of the fluorescence intensity measured in the horizontal direction. The images were analyzed using a custom Python script. (F) Data from Betegón-Putze et al. (2021) on fold-changes of spatially integrated *pBRAVO-GFP* expression over the whole SCN in *bravo-2*, *wox5-1* and *bravo-2 wox5-1* mutants relative to that in WT (indicated as mean±s.d.). (G) Transcript abundance (read counts) of *BRAVO* and *WOX5* in QC cells from RNA-sequencing data in Betegón-Putze et al. (2021) and Clark et al. (2019) [Gene Expression Omnibus (GEO) accession numbers GSE173945 and GSE98204, respectively].

The SCN is composed of a small group of rarely dividing pluripotent cells called the quiescent center (QC) and of the immediately surrounding stem cells, i.e. the vascular initials (VIs), columella stem cells (CSCs) and cortex-endodermis initials (CEIs) (Fig. 1A) (Dolan et al., 1993). Direct cell-cell contact from the QC to its surrounding stem cells is important for stem cell identity (Liu et al., 2017b; van den Berg et al., 1997) and can involve the transport of short-range signals from the QC. The homeodomain transcription factor WOX5 is specifically expressed at the QC (Sarkar et al., 2007) and can move towards adjacent cells (Berckmans et al., 2020; Clark et al., 2020; Pi et al., 2015). WOX5 itself has been proposed to act as the long-sought short-range signal to repress columella stem cell differentiation (Pi et al., 2015), albeit recent results challenge this view (Berckmans et al., 2020). Although short-range signaling is thought to ensure that stem-cell numbers are restrained and the SCN does not become displaced from the growing root tip, it is yet unclear how this is achieved (Matosevich and Efroni, 2021; Rahni et al., 2016; Scheres, 2007).

The R2R3-MYB transcription factor BRASSINOSTEROIDS AT VASCULAR AND ORGANIZING CENTER (BRAVO) has recently been linked to the maintenance of QC homeostasis (Betegón-Putze et al., 2021; Vilarrasa-Blasi et al., 2014). BRAVO is expressed at the QC and VI (Vilarrasa-Blasi et al., 2014) and colocalizes with WOX5 in the QC (Betegón-Putze et al., 2021). Both BRAVO and WOX5 have been shown to individually promote quiescence, as mutant roots with disrupted BRAVO or WOX5 exhibit increased QC divisions, supporting their role as essential factors for QC homeostasis (Forzani et al., 2014; Vilarrasa-Blasi et al., 2014). We have recently shown that BRAVO and WOX5 are co-dependent, promoting expression of each other and physically interacting, presumably through their binding into a protein complex (e.g. as a heterodimer) (Betegón-Putze et al., 2021). These results suggested that BRAVO promotes *WOX5* expression indirectly through WOX5, via the formation of the complex and the subsequent disruption of a WOX5 negative feedback on itself. In contrast, since the expression of *BRAVO* increases upon WOX5 overexpression, WOX5 activates (directly or through targets) *BRAVO* expression (Betegón-Putze et al., 2021).

Our previous study revealed the effective regulations between BRAVO and WOX5, but did not address the mechanisms driving spatial changes in their expression. The expression of the *BRAVO* promoter, restricted to the QC and VI in wild-type (WT) roots, increases and expands towards the vasculature, cortex and endodermis in the *bravo-2* loss-of-function mutant (Betegón-Putze et al., 2021) (Fig. 1B,C). This expansion suggests that *BRAVO* actively confines in space its own expression domain. Although our previous study considered the increase in total (spatially integrated) *BRAVO* promoter expression in the *bravo-2* mutant through negative feedback (Betegón-Putze et al., 2021), it did not address how the expression of *BRAVO* is regulated in space to generate self-confinement. Here, we address this problem through mathematical and computational modeling.

To this end, we used the finding that WOX5 can activate *BRAVO* expression and can bind to BRAVO protein (Betegón-Putze et al., 2021) to evaluate how these interactions can be regulated in space to drive spatial self-confinement of *BRAVO* expression. We also used the finding that BRAVO and WOX5 can act antagonistically on common targets (Betegón-Putze et al., 2021) to propose an additional mechanism for self-confinement. To assess whether these different scenarios can drive a spatial confinement that is compatible with the reported experimental data, we first studied them through minimal models in one spatial dimension, separately and in combination. These simplified schemes enabled thorough parameter space exploration. Finally, to overcome the limitations set by the simplified geometry, we translated these scenarios to a realistic two-dimensional geometry. Our results support that WOX5 diffusion (Berckmans et al., 2020; Clark et al., 2020; Pi et al., 2015) is sufficient to drive the spatial self-confinement of *BRAVO* expression. *BRAVO* self-confinement also reduces the spatial domain of WOX5 activity, overall providing a natural way for stem-cell-specific factors to locally regulate SCN maintenance. Altogether, our results shed light on the regulatory principles balancing the confinement of transcription factors to a microenvironment and their communication with the surrounding cells.

## RESULTS

### BRAVO can confine its own expression domain by immobilizing WOX5

The expansion of *BRAVO* promoter expression towards the vasculature in the *bravo-2* loss-of-function mutant compared with the WT (Betegón-Putze et al., 2021) (Fig. 1B-E) suggests that, in the WT, BRAVO confines its own expression to the SCN. Because there is no expansion in the loss-of-function *wox5-1* mutant (Fig. 1D) nor in the *bravo-2 wox5-1* mutant (Betegón-Putze et al., 2021), we considered that the mechanism for *BRAVO* self-confinement requires WOX5. To decipher how this self-confinement can be attained, we first considered that BRAVO transcription is ultimately activated by WOX5 (either directly or through WOX5 targets) (Betegón-Putze et al., 2021) and that WOX5 proteins are able to move from the QC to the VI (Berckmans et al., 2020; Clark et al., 2020). Thus, WOX5, by moving to the VI cells and shootwards, is expected to induce *BRAVO* expression in these cells. Additionally, we considered that BRAVO and WOX5 can physically interact at the protein level, presumably by binding together, as suggested by co-immunoprecipitation and fluorescence resonance energy transfer by fluorescence lifetime imaging (FRET-FLIM) analysis (Betegón-Putze et al., 2021).

Although the mobility of WOX5 (possibly through the plasmodesmata) has been experimentally tested *in planta* (Berckmans et al., 2020; Clark et al., 2020), no evidence of intercellular BRAVO transport has been reported. Due to its larger size (BRAVO has a molecular mass of ∼36 kDa compared with ∼20 kDa for WOX5; The Arabidopsis Information Resource, TAIR, loci AT5G17800 and AT3G11260, respectively), BRAVO proteins are expected to be less mobile than WOX5 proteins, if mobile at all. Moreover, the BRAVO-WOX5 complex, owing to its even larger size, is not expected to move very much from cell to cell. In this respect, although the bounds of the size exclusion limit (SEL) of the plasmodesmata vary, estimates place the SEL lower bound to be 27 kDa and upper bound to be <54 kDa between the QC and the cortex and between the QC and columella stem cells (Rim et al., 2011), yet these values may change with environmental conditions and developmental stages.

**Fig. 2.**
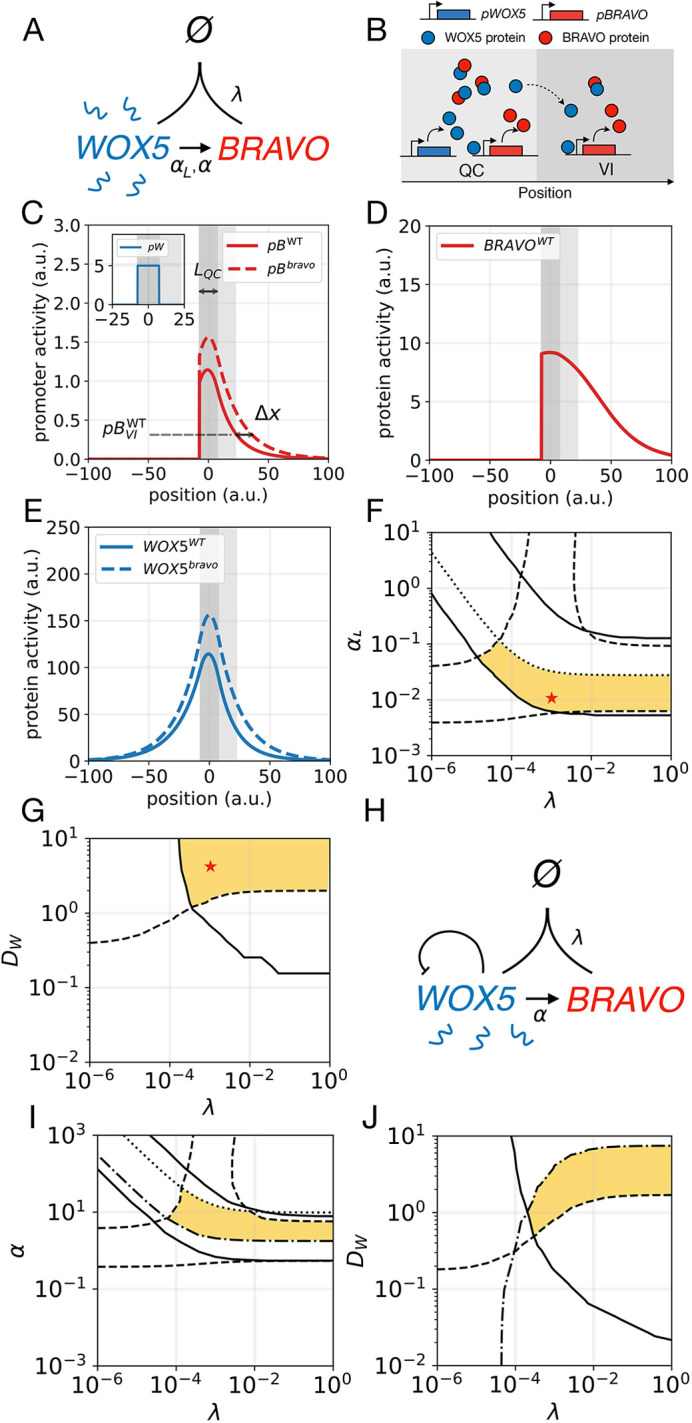
**‘Immobilization by sequestration’ model.** (A) Cartoon displaying the interactions in the model. WOX5 diffuses (wavy lines) and activates (arrow) BRAVO. In turn, BRAVO immobilizes WOX5 by sequestering it into an immobile and inactive complex (depicted by 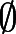). The main parameters characterizing the regulations are shown. (B) Cartoon of the model in the one-dimensional spatial framework. Proteins are denoted by circles and promoters by rectangles. (C) Stationary profiles of *BRAVO* and *WOX5* promoter activities (*pB* and *pW*) obtained with this model in the WT (continuous lines) and in the *bravo* mutant (dashed lines). The QC region (dark gray) has a size of *L*_*QC*_ and is where *pW* is active. For simplicity, the VI region (light gray) is defined with this same size. The *pB* value in the WT at the end of the VI region is denoted by 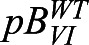. The quantity Δ*x* measures *pB* expansion in the *bravo* mutant 
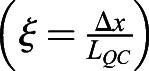
. If BRAVO confines its own expression in the WT, then Δ*x*>0. (D,E) Stationary protein profiles of BRAVO and WOX5 (not bound to each other) in WT (continuous lines) and in the *bravo* mutant (dashed lines) corresponding to the simulations in C. (F,G) Parameter domains (yellow) compatible with experimental observations for (F) BRAVO synthesis rate *α*_*L*_ and binding strength *λ* and (G) WOX5 diffusion coefficient *D_W_* and binding strength *λ* parameter spaces. Solid, dashed and dotted lines denote the observables *ξ*, *R*_*B*_ and *R*_*BW*_, respectively, taking the maximum or minimum value that we considered to be compatible with observations, as defined in the main text. Red stars in F and G mark the values used for the simulations in C-E. In C-G, activation of BRAVO by WOX5 is linear. (H) Cartoon of the model with WOX5 self-repression added. (I,J) Analogous to panels F and G, parameter domains (yellow) compatible with experimental observations are shown for the model in H: (I) BRAVO synthesis saturation rate *α* and binding strength *λ*, and (J) WOX5 diffusion coefficient *D*_*W*_ and binding strength *λ* parameter spaces, with *R*_*W*_ (dot-dashed line) included. Productions are nonlinear functions with saturation [*α* (saturated rate) is used instead of *α*_*L*_]. The values of observables for each parameter space are shown in Figs S1 and S2. Parameter values are given in Table S1. a.u., arbitrary units.

Taken together, these observations allow us to propose the following mechanism for BRAVO to confine its own expression (Fig. 2A,B). WOX5 proteins, produced in QC cells and mobile towards the VI cells, can activate *BRAVO* expression in the VI. In turn, BRAVO proteins sequester WOX5 into an immobile (i.e. from cell to cell) and inactive complex, thus disrupting WOX5 movement and impeding the activation of *BRAVO* expression in the VI. Hence, the activation of the *BRAVO* promoter by WOX5 is spatially confined by BRAVO proteins, a restriction which can be released when BRAVO can no longer immobilize WOX5, e.g. in the *bravo* mutant.

To evaluate this mechanism, we constructed a minimal mathematical model, hereafter named the ‘immobilization by sequestration’ model (see Materials and Methods; Fig. 2A,B) in which the main assumptions are: (1) WOX5 induces *BRAVO* expression (Betegón-Putze et al., 2021); (2) the WOX5 protein diffuses (Berckmans et al., 2020; Clark et al., 2020), whereas BRAVO does not; (3) the BRAVO and WOX5 proteins reversibly bind together forming a complex that does not induce *BRAVO* expression (Betegón-Putze et al., 2021); (4) the BRAVO-WOX5 complex is immobile, i.e. it cannot diffuse; and (5) all molecules, the complex included, are degraded.

We studied the model in a one-dimensional space in which WOX5 is produced only at a localized region (dark grey shaded area in Fig. 2B,C) that we identify as the QC, and can diffuse in both directions, namely towards regions that could be identified as the CSCs and columella cells (negative values of the position in Fig. 2B,C), or towards the vasculature (positive values of the position in Fig. 2B,C, where the light gray shaded region is identified as VI cells). Conversely, BRAVO was set to be activated by WOX5 only in regions from the QC and towards the vasculature, but not towards the columella, thus mimicking the asymmetric activity of the *BRAVO* promoter in the *Arabidopsis* primary root.

In Fig. 2C-E, we show the stationary activity profiles of *BRAVO* and *WOX5* promoters (*pB* and *pW*, respectively) and the stationary BRAVO and WOX5 protein concentrations (*B* and *W*, respectively), obtained by numerically solving the model equations with linear activation of BRAVO by WOX5 (see Materials and Methods). The concentration of the protein complex (which is proportional to the product *BW*) is shown in Fig. S1. The stationary profiles obtained when modeling the *bravo* mutant condition (see Materials and Methods) are also depicted. These results support that the ‘immobilization by sequestration’ mechanism can drive self-confinement and constitute a potential way for BRAVO to spatially confine its own expression in the WT root.

To address whether this mechanism drives *BRAVO* expression and self-confinement consistent with experimental observations, we defined three different observables (see Materials and Methods). The first observable, ξ, focuses on the spatial expansion of *BRAVO* promoter expression in the *bravo* mutant compared with that in the WT (Fig. 2C). We considered that expansions within 0.5 and five times the QC size were consistent with experimental observations (Fig. 1E). The second observable, *R*_*B*_, evaluates the increase of *BRAVO* expression levels in the *bravo* mutant (Fig. 2C). The model results are considered compatible with experimental data when *BRAVO* promoter activity at the QC increases up to twofold in the *bravo* mutant (Fig. 1F). The third observable, *R*_*BW*_, compares *BRAVO* and *WOX5* expressions in WT, as BRAVO transcripts are less abundant (Fig. 1G) (Betegón-Putze et al., 2021; Clark et al., 2019). We considered that the simulation results also were compatible with observations from real roots if *BRAVO* promoter expression at the end of the VI over *WOX5* promoter expression at the QC, both in the WT, was within the range of 0.05 and 0.5.

Exploration of different parameter spaces shows large domains in which the model is compatible with experimental data, for linear (Fig. 2F,G; Fig. S1A) and nonlinear regulatory functions (Fig. S2A,B). In this mechanism of self-confinement, the binding between WOX5 and BRAVO (mediated by the binding strength *λ*) as well as WOX5 diffusion are necessary (Fig. 2F). However, too much sequestration of WOX5 by BRAVO (large *λ* and high BRAVO synthesis rate *α*_*L*_) can lead to an overly exaggerated confinement, with BRAVO being highly expressed at the QC but not in VI cells (Fig. 2G; Fig. S1C), a situation which would disagree with the experimentally observed WT expression (Fig. 1B). The results also show that if WOX5 is set to have a large diffusion coefficient, *BRAVO* expression in the WT expands but gets dimmer (Fig. S1E). This suggests that WOX5 diffusion might be rather small, in agreement with observed protein distributions of WOX5 within the root SCN, which primarily reach cells adjacent to the QC (Berckmans et al., 2020; Clark et al., 2020). Overall, our results indicate that the ‘immobilization by sequestration’ mechanism is a plausible candidate model to explain the observed self-confinement of *BRAVO* expression.

As sequestration is a necessary factor for this mechanism to work, one could argue that the presence of other molecules also binding to BRAVO and/or WOX5 might impair or even destroy the confinement. Indeed, it is known that WOX5 and BRAVO proteins can bind to additional molecules, such as TOPLESS (TPL) and BES1 (Betegón-Putze et al., 2021; Vilarrasa-Blasi et al., 2014). These are relatively large molecules (∼39 kDa and ∼124 kDa, respectively; The Arabidopsis Information Resource, TAIR) and it would be possible for them to immobilize WOX5. We modeled this situation assuming the most limiting case, i.e. that these molecules (S) only act as competitors for the binding of BRAVO and WOX5 (see supplementary Materials and Methods; Table S2). The results show that *BRAVO* self-confinement takes place if S is much less produced than BRAVO (for the same degradation rate, Fig. S3). If instead S production is similar to that of BRAVO, then the *BRAVO* promoter expression domain is similarly confined in the WT and in the *bravo* mutant. In this mutant, S overcomes the absence of BRAVO by binding to WOX5 and immobilizing it (Fig. S3). As TPL and BES1 transcript abundance at the QC is not very high [ranging 0.57-3.08 times that of BRAVO and 0.13-0.39 times that of WOX5, based on read counts (Clark et al., 2019; Betegón-Putze et al., 2021)], the results suggest that the ‘immobilization by sequestration’ mechanism may take place.

To further assess the plausibility of the ‘immobilization by sequestration’ mechanism in the context of BRAVO and WOX5, we included the fact that BRAVO promotes *WOX5* expression, as the expression of GFP under the *WOX5* promoter decreases in the *bravo-2* mutant (Betegón-Putze et al., 2021; Vilarrasa-Blasi et al., 2014). Specifically, we previously proposed that BRAVO promotes *WOX5* expression through a mechanism that involves a WOX5 negative feedback (modeled as self-repression) and the binding of the WOX5 and BRAVO proteins (Betegón-Putze et al., 2021). WOX5 self-repression is consistent with increased *WOX5* promoter expression in the *wox5-1* mutant (Betegón-Putze et al., 2021). Therefore, as the ‘immobilization by sequestration’ model already included the binding between BRAVO and WOX5, we only added WOX5 self-repression to the model (Fig. 2H, Materials and Methods) and defined a fourth observable, *R*_*W*_, which measures the ratio between *WOX5* promoter activity in the *bravo* mutant and that in the WT. We considered that simulation results within the range of 0.5 and 0.9 are compatible with experimental data (Betegón-Putze et al., 2021). The results show that the *BRAVO* expression profile remains similar when WOX5 self-repression is included (Fig. S2). *R*_*W*_ sets additional constraints to the lower bound of BRAVO production rate (Fig. 2I) and limits the diffusion coefficient of WOX5 (Fig. 2J). This last restriction is not expected to be very limiting as the experimental data suggest a small amount of WOX5 diffusion (Berckmans et al., 2020; Clark et al., 2020).

Altogether, the ‘immobilization by sequestration’ mechanism constitutes a plausible candidate for explaining the self-confined *BRAVO* expression to both the QC and VI cells in the WT. Notably, the WT stationary profile of the WOX5 protein (*W*, not bound to BRAVO) obtained for this model is not symmetric, but decays differently above and below the QC (Fig. 2E). This behavior is caused by the presence and absence, respectively, of BRAVO proteins in the two distinct spatial regions. Above the QC, the gradient of WOX5 proteins is steeper than below the QC, where BRAVO cannot be activated. Hence, the presence of BRAVO makes the WOX5 gradient more abrupt, restricting the spatial domain in which WOX5 proteins are concentrated, and consequently confining the *BRAVO* expression domain. Therefore, this mechanism not only drives self-confined *BRAVO* expression but also results in a confined action of WOX5 proteins.

### A mobile intermediary can facilitate BRAVO self-confinement

**Fig. 3.**
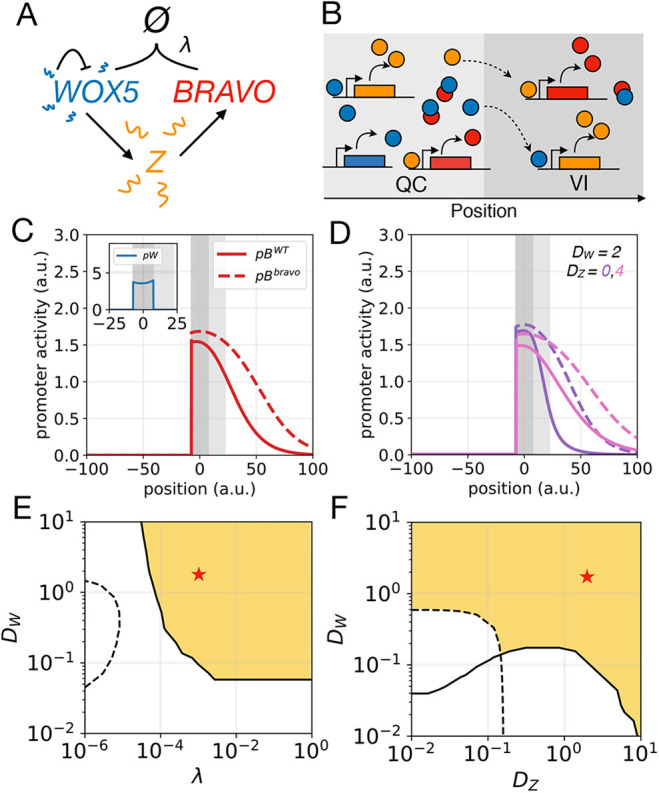
**‘Immobilization by sequestration’ model with intermediary.** (A) Cartoon of the model, same as Fig. 2H except that WOX5 activates a diffusible intermediary Z, which in turn activates BRAVO. (B) Cartoon of the model in the one-dimensional spatial framework. WOX5 self-repression is not represented for clarity. (C) Stationary activity profiles *pB* and *pW* (inset) in WT (continuous lines) and in the *bravo* mutant (dashed lines) for equal WOX5 and Z diffusion coefficients (*D_W_*=*D_Z_*=2). (D) Stationary *pB* profiles in the WT (continuous) and in *bravo* mutant (dashed) for non-mobile Z (violet) and for stronger mobile Z (pink). (E,F) Parameter domains (yellow) compatible with experimental observations for (E) WOX5 diffusion coefficient *D_W_* and binding strength *λ*, and (F) WOX5 diffusion coefficient *D*_*W*_ and Z diffusion coefficient *D*_*Z*_ parameter spaces. Conditions for *R*_*W*_ are satisfied in all the depicted spaces (indicated by the absence of a dot-dashed line). Fig. S4 depicts the values of the observables. Red stars mark the parameter values used in C. Production functions are nonlinear. Parameter values are given in Table S1. a.u., arbitrary units.

The self-confinement of *BRAVO* expression in the ‘immobilization by sequestration’ model requires WOX5 to diffuse. Experiments suggest that although it is mobile, WOX5 does not move very large distances (Berckmans et al., 2020; Clark et al., 2020). Hence, its low mobility may not be sufficient to explain the self-confinement of *BRAVO* expression observed in real roots. As activation of *BRAVO* by WOX5 could occur through intermediary molecules, we asked whether realistic BRAVO self-confinement can arise for smaller WOX5 diffusion coefficients if WOX5 activates *BRAVO* through a mobile intermediary molecule, Z. To evaluate this scenario, we included such an intermediary molecule in the ‘immobilization by sequestration’ model and studied its implications by numerically simulating the WT and *bravo* mutant in one dimension, as previously done. In this model (Fig. 3A,B) the assumptions are analogous to those mentioned earlier but include Z as follows: (1) WOX5 activates Z, which activates BRAVO; (2) Z and WOX5 diffuse, whereas the BRAVO protein does not; (3) BRAVO and WOX5 proteins reversibly bind together forming a complex that cannot induce Z; (4) the BRAVO-WOX5 complex is immobile, i.e. it cannot diffuse; (5) all molecules are degraded; and, (6) additionally, WOX5 self-represses as in Fig. 2H.

The results show that Z drives a wider *BRAVO* expression, as expected, which is still self-confined (Fig. 3C,D). The mobility of Z enables BRAVO self-confinement at lower diffusion coefficients of WOX5 (compare Fig. 3E with Fig. 2J, Fig. 3J) as BRAVO, when binding to WOX5, confines its expression by also attenuating the production of Z (Fig. S4).

### BRAVO can confine its own expression domain by repressing its mobile activator

**Fig. 4.**
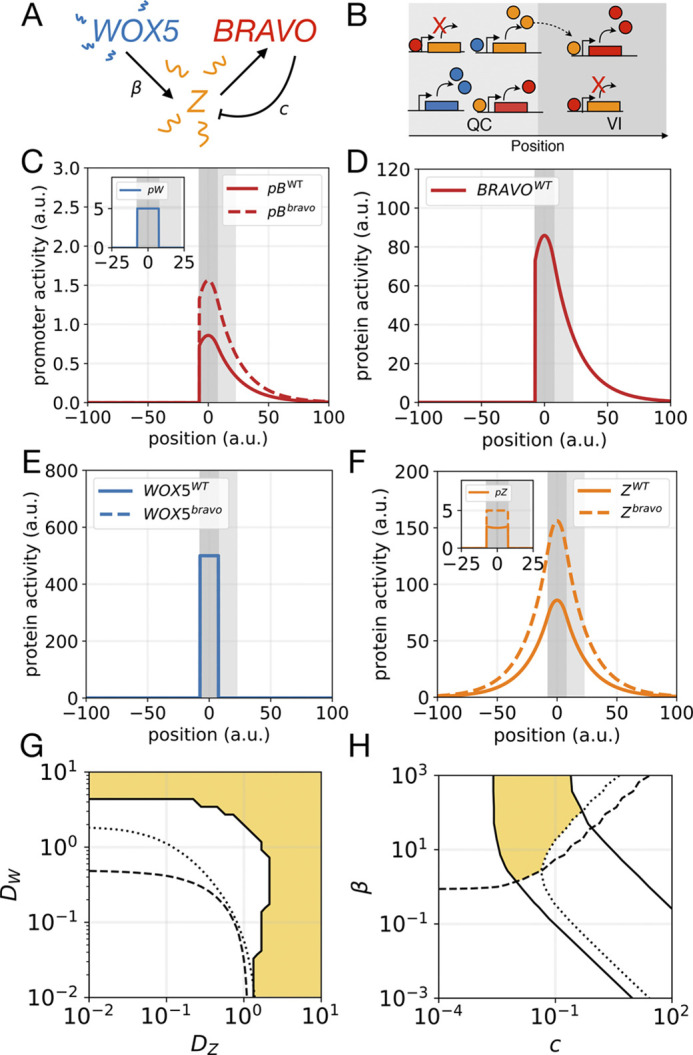
**‘Repression’ model.** (A) Cartoon of the interactions. WOX5 diffuses and activates a diffusible factor Z, which activates BRAVO. BRAVO, in turn, represses (blunt arrow) Z. (B) Cartoon of the model in the one-dimensional spatial framework. WOX5 movement is not depicted. (C) Stationary activity profiles of *pB* and *pW* in WT (continuous lines) and in the *bravo* mutant (dashed lines) obtained with the ‘repression’ model assuming negligible diffusion of WOX5 (*D_W_*=0) for *D_Z_*=4. (D-F) Stationary profiles of BRAVO, WOX5 and Z proteins as well as promoter activity *pZ* in WT (continuous lines) and in the *bravo* mutant (dashed lines) corresponding to the simulations in B. (G,H) Parameter domains (yellow) compatible with experimental observations for (G) WOX5 and Z diffusion coefficients, *D_W_* and *D_Z_*, respectively, and for (H) Z saturated activation rate *β* and repression strength *c*. C-G use linear activation functions. H uses nonlinear functions and *D_W_*=0. Figs S5 and S6 depict the values of the observables for G and H, respectively. Parameter values are given in Table S1. a.u., arbitrary units.

The previous model showed that a transcription factor can confine its own expression by reducing the production of its mobile activator Z. Based on this, we envisaged an additional scenario that drives a reduction of the mobile activator but is not based on the sequestration of BRAVO and WOX5. In this case, BRAVO represses the production of its activator Z, which is mobile and is activated by WOX5 (Fig. 4A,B). Reported transcriptomics of QC cells have revealed that several of the genes of which the mRNA levels are de-regulated in *wox5-1* and *bravo-2* mutants show opposite regulations (Betegón-Putze et al., 2021), thus opening the possibility of these genes to express or regulate the intermediate factor Z. We called this the ‘repression’ model (see Materials and Methods) and its assumptions are: (1) WOX5 activates Z, which activates BRAVO; (2) Z and WOX5 diffuse, whereas BRAVO does not; (3) BRAVO represses the production of Z (but the BRAVO-WOX5 complex does not); and (4) all molecules degrade. Note that all these assumptions except (3) were already present in the previous model.

Simulations indicate that the ‘repression’ mechanism is able to induce self-confinement of *BRAVO* expression without requiring WOX5 to be mobile (Fig. 4C-G). The action of WOX5 mediated by its target Z, but not the WOX5 protein, becomes spatially restricted in the WT, whereas it expands in the *bravo* mutant (Fig. 4F,E). Strong repression enhances the self-confinement, but can result in unrealistic *BRAVO* expression profiles in the WT, limited only to the QC and not reaching the VI cells (Fig. S5). In addition, strong repression involves not only spatial confinement but also a dramatic reduction of *BRAVO* expression at the QC in the WT compared with the *bravo* mutant (Fig. S5), a situation which is not observed experimentally (Betegón-Putze et al., 2021) (Fig. 1B,C). This sets strong limiting conditions on the repression strength and on the rate of Z production when assuming linear regulatory functions (Fig. S5), but not as much for nonlinear functions, as expected (Fig. 4H; Fig. S6).

**Fig. 5.**
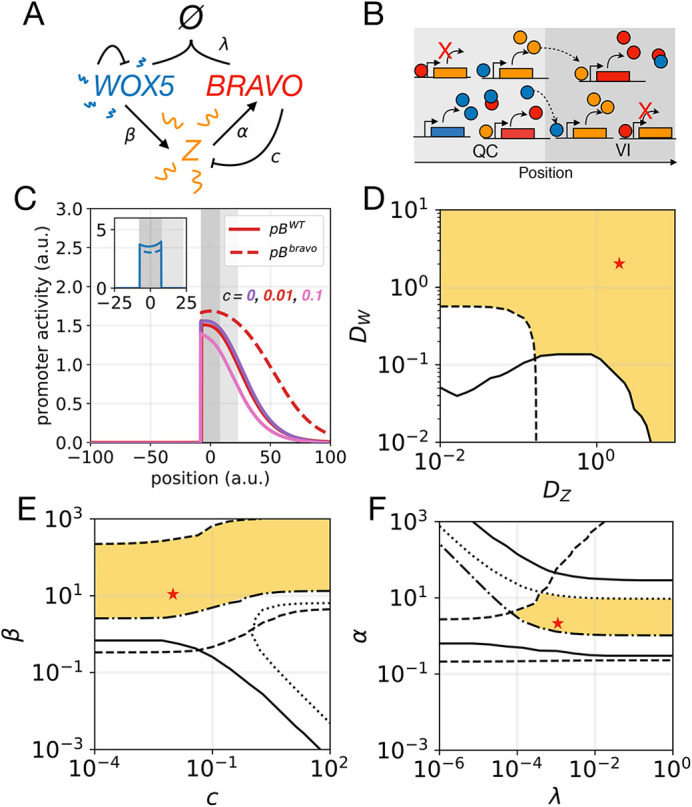
**Mixed model.** (A) Cartoon of the interactions, which involve the ‘immobilization by sequestration’ and the ‘repression’ models. (B) Cartoon of the model in the one-dimensional spatial framework. (C) Stationary *pB* and *pW* (inset) profiles in WT (continuous lines) and in the *bravo* mutant (dashed lines) for several repression strengths. All cases have the same profile in the *bravo* mutant. (D-F) Parameter domains (yellow) compatible with experimental observations in (D) WOX5 and Z diffusion coefficients, *D_W_* and *D_Z_*, respectively, (E) Z saturated activation rate *β* and repression strength *c*, and (F) BRAVO production saturation rate *α* and binding strength *λ* parameter spaces. Red stars mark the parameter values used in C for *c*=0.01. Fig. S7 depicts the values of the observables. Nonlinear production functions used. Parameter values are given in Table S1. a.u., arbitrary units.

Finally, we considered that BRAVO promotes *WOX5* expression and assumed that this occurs through binding with WOX5 and WOX5 self-repression. We termed this the ‘mixed’ model, as it involves both the ‘repression’ and the ‘immobilization by sequestration’ models (Fig. 5A,B). Despite the sequestering of BRAVO by (large amounts of) WOX5 reduces the BRAVO-mediated repression, the presence of both the repression and immobilization mechanisms enhances self-confinement (Fig. 5C). In addition, *BRAVO* self-confinement that is compatible with experimental observations happens in larger parameter domains, i.e. when repression is not sufficient, sequestration takes its place (Fig. 5D-F). Note that sequestration is required to account for decreased *WOX5* expression in the *bravo* mutant but not for *BRAVO* self-confinement. Similar results are found when basal production of BRAVO, i.e. independent of WOX5, is set at the VI region (Fig. S7) to mimic the situation in which these cells still show *BRAVO* promoter expression in the *wox5-1* mutant (Betegón-Putze et al., 2021) (Fig. 1D).

### The ‘immobilization by sequestration’ mechanism is sufficient and the ‘repression’ mechanism enhances self-confinement in *Arabidopsis*

Our previous results suggest that the mixed model is a conceivable candidate to explain the self-confined expression of *BRAVO* in the *Arabidopsis* root SCN. However, it remains unclear whether the degree of diffusion of WOX5 is sufficiently large to generate realistic expansions. To address this issue, we modeled the mixed model on realistic two-dimensional root layouts, for which the cellular geometry of the roots, as well as the presence of cell walls, was explicitly incorporated. We also modeled the dynamics of GFP produced by the *BRAVO* and the *WOX5* promoters (*pBRAVO:GFP* and *pWOX5:GFP*, respectively; see Materials and Methods). The main features included in this new framework can be enumerated as follows (for further details, see Materials and Methods and supplementary Materials and Methods). (1) The spatial discretization of the realistic root layout was made at the pixel scale. Hence, the size of the cytoplasm and cell walls is determined by the number and localization of their corresponding pixels (Fig. S8). (2) The dynamics of the molecular components in the interior of the cells is distinct from the dynamics in the cell walls. Specifically, in all the pixels belonging to the interior of the cell, molecules can be produced, regulated or degraded, can sequester other molecules and are able to diffuse. Inside the cell walls, however, molecules are only able to diffuse. (3) The diffusion coefficient in cell walls is set to be smaller than in the interior of the cells. With this assumption, the physical boundaries between cells are naturally incorporated. These are to be expected for any molecule diffusing freely inside the cytoplasm but moving only occasionally from cell to cell, as it may happen, for example, in plasmodesmata-mediated transport. For simplicity (and lack of evidence), we assume that BRAVO and the BRAVO-WOX5 complex can only move inside cells, being unable to diffuse through cell walls. Conversely, WOX5 and Z proteins can move inside the cytoplasm with diffusion coefficients *D_W_^cyt^* and *D_Z_^cyt^*, respectively, and between cells with diffusion coefficients *D_W_^wall^* and *D_Z_^wall^*, respectively. Because GFP proteins are set to diffuse from cell to cell, they can reach cells in which there is no promoter activity. However, the degree of GFP diffusion is set sufficiently low so that this effect is small, such that GFP expression under the *WOX5* promoter is mostly localized at the QC (Betegón-Putze et al., 2021; Vilarrasa-Blasi et al., 2014). (4) Cells are classified by cell type. We defined nine different cell types: quiescent center (QC), vascular initials (VI), vascular cells (V), cortex-endodermis initials (CEI), pericycle (PER), cortex (CX), endodermis (END), epidermis (EPI), columella stem cells (CSC), columella cells (CC) and lateral root cap cells (LRC) (Fig. 1A). This classification enables setting different dynamics for the proteins in distinct cell types, as well as different transport properties (through different diffusion coefficients). However, we set the diffusion coefficient of each molecular species to be the same in all cell types. In contrast, different cell types differ in their protein production dynamics. WOX5 only activates BRAVO in the QC, VI, vascular cells, cortex and endodermis, but not in the remaining cell types (Fig. S9). This assumption is based on the experimental observation that *BRAVO* is expressed only in inner tissues, i.e. from the SCN upwards. Basal production of BRAVO – independent of WOX5 – is set in a few of these cells (Fig. S10). For simplicity, WOX5 is set to be only produced at the QC [albeit low amounts of promoter expression are also present in the vascular initials (Berckmans et al., 2020)]. For each species, the degradation rate was assumed to be the same in all cell types.

**Fig. 6.**
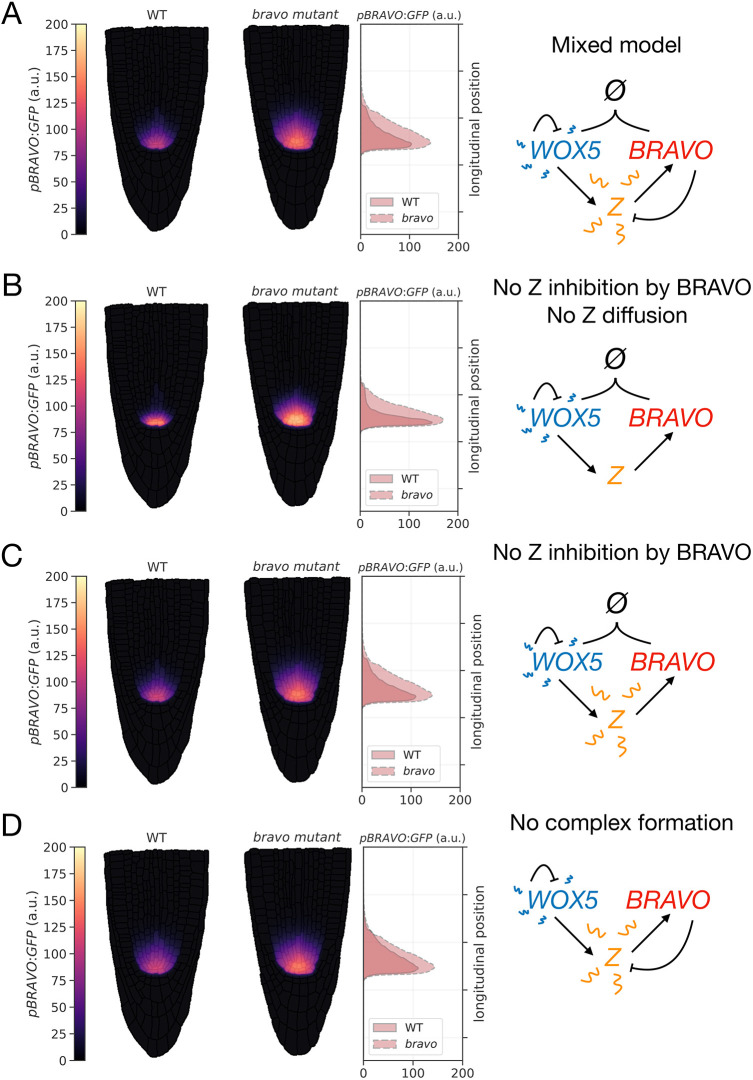
***pBRAVO:GFP* simulated in realistic root layouts.** Simulation results for the stationary activity of *pBRAVO:GFP* in WT (left root) and in *bravo* mutant (right root) for different regulatory interactions (each depicted as a cartoon on the right). The middle panel depicts *pBRAVO:GFP* (integrated transversally) along the vertical axis, from simulation images on the left. (A) Mixed model. In the WT, *pBRAVO:GFP* is confined to the SCN, and it is expanded in the *bravo* mutant. Fig. S12 depicts the stationary profiles of all other molecules. (B) ‘Immobilization by sequestration’ model with non-diffusible Z. Self-confined expression of *pBRAVO:GFP* is also obtained. (C) Mixed model without repression (i.e. ‘immobilization by sequestration’ model with diffusible Z). (D) Mixed model without BRAVO-WOX5 binding. C and D show similar results to A. Hence, sequestration can compensate for the absence of repression and vice versa. Simulations in the *bravo* mutant lead to the same *pBRAVO:GFP* for A,C and D. In all panels, cell walls are superimposed (in black with transparency) over the colormap so that they can be easily visualized. Parameter values are given in Table S3. For other parameter values, repression can be more relevant (Fig. S14). a.u., arbitrary units.

We chose parameter values for WOX5 dynamics so that WOX5 proteins, in the absence of any regulatory factor, remain mostly localized at the QC, VI and CSCs (Fig. S11). This is consistent with reported data of GFP bound to WOX5 proteins (Berckmans et al., 2020; Clark et al., 2020). The other parameter values were chosen to drive *pWOX5:GFP* and *pBRAVO:GFP* expression patterns compatible with those observed in real WT roots (Table S3). The mixed model can explain the behavior of *pBRAVO:GFP* in the WT and the expansion of its domain in the *bravo* mutant (compare Fig. 6A with Fig. 1E). BRAVO self-confinement additionally involves the confinement of WOX5 proteins and of the diffusible target Z (Fig. S12). Although different layouts (Fig. S10) are used to model WT and *bravo* mutant roots (Fig. S10), this does not introduce significant differences (Fig. S13).

The small diffusion of WOX5 is sufficient to drive a realistic confinement of *pBRAVO:GFP* through the ‘immobilization by sequestration’ mechanism without the need of Z mobility (and thereby, of Z) (Fig. 6B). Yet, the presence of a mobile Z cannot be discarded. For such a mobile Z, sequestration and repression can provide an attenuation of Z that contributes to confine *BRAVO* expression (Fig. 6A,C,D; Fig. S14). Taken together, the results in the realistic root layout support the idea that the immobilization of WOX5 by BRAVO is sufficient for the self-confined expression of *BRAVO* in the root SCN.

## DISCUSSION

We have previously shown that BRAVO and WOX5 regulate the expression of each other, and that their binding into a protein complex can be relevant for these regulations and for BRAVO and WOX5 action on QC divisions (Betegón-Putze et al., 2021). Here, we show that these interactions, together with the diffusion of WOX5, are sufficient to drive the spatially self-confined expression of *BRAVO*, as revealed by the ‘immobilization by sequestration’ mechanism. Furthermore, the opposite regulation of common targets by WOX5 and BRAVO, as inferred by transcriptional profiling (Betegón-Putze et al., 2021), proposes a complementary scenario in which this confinement is induced by negative feedback between BRAVO and a factor activated by WOX5 (the ‘repression’ mechanism). According to transcriptomics analyses at the QC, only the *AT3G41762* gene has a significantly decreased expression in the *wox5-1* mutant and increased expression in the *bravo-2* mutant, making it a potential candidate to act as Z if it induces *BRAVO*. Notably, this gene is strongly expressed in the root SCN of WT *Arabidopsis* (Fig. S15) (Clark et al., 2019), but its function awaits to be characterized. The remaining genes that are antagonistically and significantly regulated by BRAVO and WOX5 show increased expression in the *wox5-1* mutant and decreased expression in the *bravo-2* mutant (Betegón-Putze et al., 2021). Hence, they are repressed by WOX5 and activated by BRAVO. If any of these genes inhibits a particular factor, this factor might be acting as Z (Fig. S15). Such a potential case is exemplified by peroxidase proteins, which are repressed by WOX5 but activated by BRAVO, and which remove the mobile hydrogen peroxide. Based on their expression patterns in the SCN (Fig. S15) (Clark et al., 2019), they may play a role in confining *BRAVO* expression through hydrogen peroxide, pointing to the relevance of preventing oxidative stress in the stem-cell microenvironment. Whether any of these candidates regulates BRAVO and can drive the ‘repression’ mechanism remains to be elucidated.

The ‘immobilization by sequestration’ and ‘repression’ mechanisms share the idea that the emergence of self-confinement involves negative feedback of BRAVO on itself with a mobile activator (WOX5 or Z) and an immobile inhibitor (BRAVO). As such, they mechanistically account, at least partially, for the effective negative self-regulation of *BRAVO* expression in the whole SCN that was previously proposed (Betegón-Putze et al., 2021). Each of these two mechanisms can be understood as a different regulatory way for this negative feedback to be accomplished: by immobilizing the activator or by reducing the production of the activator. Therefore, they can be encompassed within a general framework of self-confinement, i.e. an ‘attenuation of a mobile activator’ model. Both mechanisms drive the self-confinement of both the repressor and the activator. Yet, the mechanisms are not equivalent, each of them having distinct characteristics, as our analysis revealed. The most noticeable feature is perhaps the fact that the ‘immobilization by sequestration’ mechanism involves a change in the gradient profile of the activator (Fig. 2E; supplementary Materials and Methods), whereas the ‘repression’ mechanism does not.

A different mechanism to drive self-confined expression has been proposed for WUSCHEL in the shoot apical meristem of *Arabidopsis*. According to this mechanism, the WUSCHEL protein confines its own expression by activating its repressor, CLAVATA3, which is highly mobile (Yadav et al., 2011; Zhou et al., 2018). Both this mechanism and the ‘attenuation of a mobile activator’ mechanism studied here share the idea that negative feedback is responsible for the self-confinement. However, in the ‘attenuation of a mobile activator’ mechanism, the strongly mobile component is the activator and not the repressor. This mechanism is thus a distinct mechanism for self-confinement and, because of its minimal assumptions, we expect it, or a variant thereof, to take place in very distinct developmental contexts. For instance, Hedgehog signaling in the *Drosophila* wing confines its own expression by activating one of its repressors, the nuclear zinc finger master of thickveins (mtv, also known as sbb) (Bejarano et al., 2007). Thus, this Hedgehog self-confinement may be framed within the ‘attenuation of a mobile activator’ mechanism, in which Hedgehog acts as the mobile activator (Z in the repressor model) and master of thickveins as the immobile repressor.

The mechanism of ‘immobilization by sequestration’ can be related to mechanisms that generate robust morphogen gradient profiles (Eldar et al., 2003; Shilo and Barkai, 2016). Morphogens are ligand molecules that are produced at localized sources but can diffuse, generating an activity gradient that can then be interpreted by different genes, leading to their activation or repression in a concentration-dependent manner. It has been proposed that the sequestering of the morphogen by receptors might lead to an effective non-linear degradation of the ligand, resulting in concentration profiles that are robust to changes in the rate of production at the source (Eldar et al., 2003). We could make the correlation between such models and ours by identifying WOX5 as the morphogen and BRAVO as the receptor. In agreement with what has been described for morphogen gradients induced by non-linear degradation (Eldar et al., 2003), we find that the gradient of WOX5 decays more abruptly in the presence of BRAVO than without, thus suggesting that the specific regulations between BRAVO and WOX5 may be tuned to achieve robust activity profiles. Yet, we have not seen a robust response to changes in *WOX5* expression levels for parameter values compatible with experimental observations (Fig. S16) and further analysis is required to address whether this model enhances BRAVO robustness to WOX5.

Modeling of root tissues has been most commonly done in terms of idealized realistic simplified geometries in which cells and cell walls are each subdivided in squares or rectangles with molecules diffusing between them (Grieneisen et al., 2007; Tian et al., 2014), or by considering real cellular layouts with diffusing molecules between but not within cells (Jönsson et al., 2005; Voß et al., 2014; Yadav et al., 2011). By using pixels as the basic unit for discretization, we are able to model the shapes of cells in a realistic manner and consider both the interactions within and between cells. A similar pixel-based approach has been used to model hormonal crosstalk in the *Arabidopsis* root (Liu et al., 2017a). Mathematically, our framework can be characterized as a reaction-diffusion model in heterogeneous media, in which the spatial inhomogeneities appear due to the presence of cell walls, which involve different diffusion coefficients. The realistic root layout used for the simulations can be extended to include internal structures within the cells (such as the nucleus) as well as structures in the cell walls (e.g. specific communication channels). Therefore, it has the potential to implement and evaluate much more complex scenarios in a manageable way.

The similarities between stem-cell niche organization in animals and plants may represent the outcome of convergent evolution (Dinneny and Benfey, 2008). Multicellularity – a necessary condition for stem-cell niches to emerge – is thought to have evolved independently in both kingdoms (Grosberg and Strathmann, 2007; Knoll, 2011), implying that the presence of stem-cell niches in widely disparate organisms may be a direct consequence of developmental constraints and not of historical contingencies. Similar mechanisms of niche regulation are therefore to be expected, not through common genes or molecules, but through more general regulatory principles. The fact that stem-cell niches consist of narrow regions of a few cells clustered together, in opposition to large numbers of cells distributed over the whole organism, possibly emerged as a way to ensure a proper balance between centralized renewal and genome integrity, by minimizing deleterious mutations that may be able to spread across whole cell lineages (Sena et al., 2009; Yoshiyamaa et al., 2009). Indeed, the smaller the population of stem cells and the lower their division rate, the less likely for deleterious mutations to accumulate in differentiated tissues (Cannataro et al., 2017). The mechanism proposed in this paper establishes a balance between the communication mediated by the activator (WOX5) with confining its action, ensuring communication remains local.

In the *Arabidopsis* SCN, these signals allow cells to communicate between them and with other cell types, at the same time as they create boundaries within which local information can be transmitted. Indeed, QC cells have been shown to influence neighboring cell types such as the CSCs, in which WOX5 can play a crucial role as a signaling agent (Pi et al., 2015). We propose that towards the vasculature, BRAVO can be a signaling molecule, which, by actively restraining its own expression from reaching cells far away from its source, ensures that the small microenvironment of the SCN remains confined within the root. The molecular processes underlying this spatial restriction and their implications for proper stem-cell renewal are just beginning to be uncovered. Mechanisms like the ones proposed here involve very general principles that contribute to the understanding of stem cell populations not only in plants, but in multicellular organisms on the whole.

## MATERIALS AND METHODS

The models formulated set the rate of change of protein concentrations of BRAVO (*B*) and WOX5 (*W*), by using partial differential equations in which the transport of the mobile proteins is modeled through diffusion. In the models in which an intermediate factor (*Z*) is present, its dynamics are also considered. We only focus on the stationary solutions of the models, assuming these account for the experimentally reported expressions.

In all the models, the rate of synthesis of each protein is assumed to be proportional to its corresponding promoter activity and the quasi-stationary approximation for mRNAs (i.e. mRNAs dynamics are assumed to be very fast compared with the dynamics of proteins) is done (see supplementary Materials and Methods). For simplicity, proteins are assumed to degrade linearly. Mass action law is set for the rate at which two proteins form a complex (i.e. ∝ *BW* for the BRAVO-WOX5 complex). We only consider pairwise interactions, omitting higher order reactions. Complexes are taken to be unable to transcriptionally regulate any of the proteins considered (for simplicity, we name them inactive complexes, albeit they could regulate other factors not modeled herein). The supplementary Materials and Methods contain the derivation of the equations of the minimal models from the full set of equations that include mRNAs and complexes. The stationary solutions are the same for both (full and minimal) models.

The following subsections describe the one-dimensional minimal models in WT scenarios, the modeling of *bravo* mutants, the parameter space exploration with the definition of the observables and their comparison with experimental data, the construction of the realistic root layout, the equations used for the simulations in the realistic root layout and the numerical details of all models simulated.

### ‘Immobilization by sequestration’ mathematical model

In this case, WOX5 activates BRAVO, and both proteins are able to form an inactive complex, which is degraded. In the WT, the dynamics of *B* and *W* are:
(1)

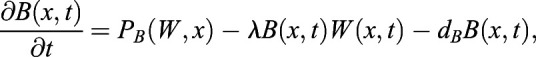

(2)

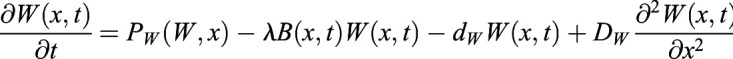
where *x* denotes the spatial position in one dimension and *t* denotes time. Here *B* and *W* indicate BRAVO and WOX5 protein concentrations, respectively, when they are not bound to each other, and the complex they form is not explicitly modeled as a variable (see supplementary Materials and Methods). *P*_*B*_(*W*, *x*) is the production of BRAVO through WOX5, which is only allowed from the QC to VI direction. For the simplest scenario of linear regulatory functions we use *P*_*B*_(*W*,*x*)=*α*_*L*_*W*Θ(*x*+*L*_*QC*_/2), where the parameter *α*_*L*_measures the production rate of BRAVO per unit concentration of WOX5 (in units of inverse time) and the Heaviside function Θ(*x*+*L*_*QC*_/2) denotes that production can only occur at *x*≥− *L*_*QC*_/2 (i.e. from the QC towards the VI direction). For nonlinear regulations with saturation, we use 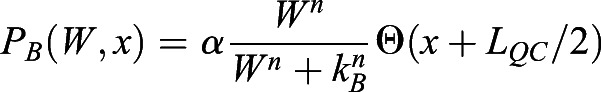, where *α* is the saturated rate of production (in units of concentration over time), *k*_*B*_ is the concentration of WOX5 at which the production is half the saturated value and exponent *n* sets the cooperativity. WOX5 is only produced at the QC. In the simplest scenario, WOX5 production is constant (independent of WOX5 itself) such that *P*_*W*_(*W*, *x*)=*γ*_*QC*_(*x*), where *γ*_*QC*_(*x*) denotes that production is only at the QC. We choose *γ*_*QC*_(*x*) to be a Heaviside function:
(3)

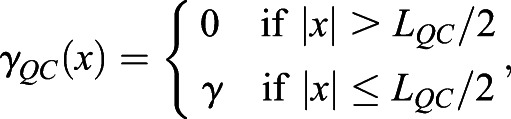
where *L*_*QC*_is the total length of the QC region. This implies that WOX5 production only occurs in the region delimited by − *L*_*QC*_/2≤*x*≤*L*_*QC*_/2, with constant production rate *γ*. When WOX5 negative feedback is included, we use 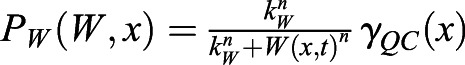, *k*_*W*_ being the WOX5 concentration at which WOX5 production at the QC is *γ*/2. Degradation of BRAVO and WOX5 is controlled by the parameters *d*_*B*_ and *d*_*W*_, respectively. Complex formation between BRAVO and WOX5 is mediated by the parameter *λ*, which sets the rate at which the two factors interact per concentration unit of each of them and involves binding, unbinding and degradation rates (see supplementary Materials and Methods). Finally, WOX5 is able to diffuse from the QC with rate *D*_*W*_. As explained in detail in the supplementary Materials and Methods, in order to have confinement through the ‘immobilization by sequestration’ mechanism, it is essential for the formation of the BRAVO-WOX5 complex to be either irreversible, or reversible but being subject to degradation. The ‘immobilization by sequestration’ model constitutes a spatially dependent version of the ‘complex formation model’ proposed in Betegón-Putze et al. (2021), which explained the regulations between BRAVO and WOX5 in the whole *Arabidopsis* stem-cell niche and did not contain any transport or spatial framework.

The promoter activities of BRAVO and WOX5, which are computed at the stationary state (i.e. when all time derivatives are zero), are defined as *pB*(*x*)=*P*_*B*_(*W*_*s*_(*x*), *x*) and *pW*(*x*)=*P*_*W*_(*W*_*s*_(*x*), *x*), respectively, where *W*_*s*_(*x*) denotes the spatial profile of WOX5 in the stationary state, as indicated by the subscript s. We also refer to these activities as promoter expressions.

Another version of this model where an additional sequestrator (S) affects the dynamics of BRAVO and WOX5 is described in the supplementary Materials and Methods (results in Fig. S3).

### ‘Immobilization by sequestration with intermediary Z’ mathematical model

The activation of BRAVO by WOX5 is set through the intermediate factor Z:
(4)

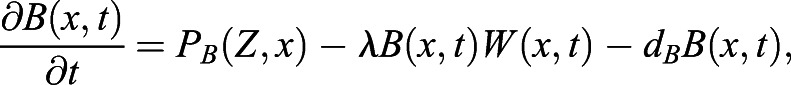

(5)

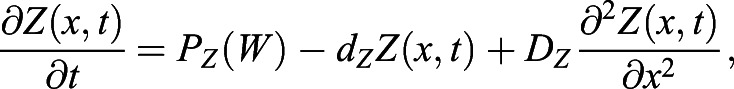

(6)

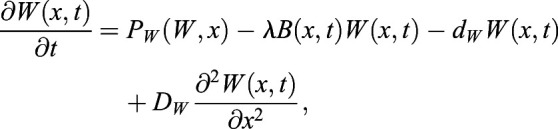
with non-linear regulatory functions 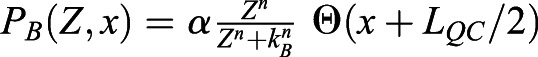, 
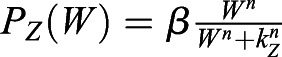
 and 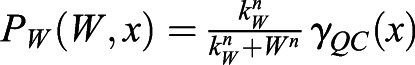, where *Z* and *W* stand for *Z(x,t)* and *W(x,t)*, respectively. The parameters *β*, *d*_*z*_ and *D*_*z*_ describe the production, degradation and diffusion of Z, respectively. The stationary promoter activities (expressions) of BRAVO, WOX5 and Z are defined as *pB*(*x*)=*P*_*B*_(*Z*_*s*_(*x*), *x*), *pW*(*x*)=*P*_*W*_(*W*_*s*_(*x*), *x*) and *pZ*(*x*)=*P*_*Z*_(*W*_*s*_(*x*)), respectively, where the subscript s denotes the protein profile in the stationary state. Fig. S4B evaluates the effect of introducing a basal production of BRAVO at the VI (i.e. 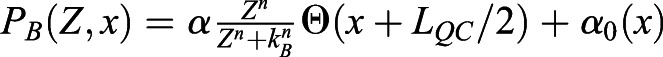, where the term *α*_0_(*x*) corresponds to a basal production of value *α*_0_ only at the VI region).

### ‘Repression of a mobile activator’ mathematical model

This model reads:
(7)

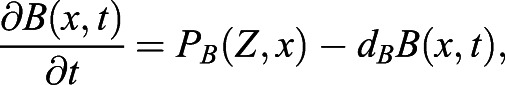

(8)

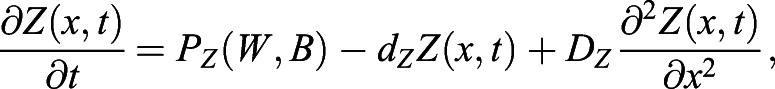

(9)

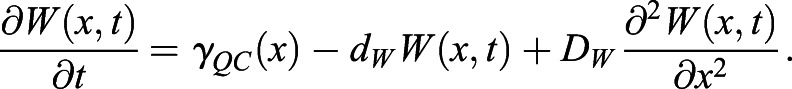


For linear activations, the productions are *P*_*B*_(*Z*, *x*)=*αZ*Θ(*x*+*L*_*QC*_/2) and 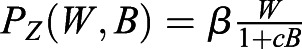, whereas for non-linear saturating activations, we use 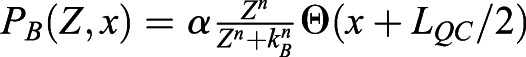 and 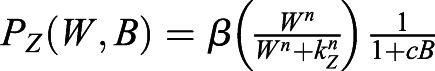. These nonlinear *P*_*B*_(*Z*, *x*) and *P*_*Z*_(*W*, *B*) are the same functions as those in the immobilization model with intermediary Z, except that *P*_*Z*_(*W*, *B*) includes the repression of BRAVO on Z. *c* sets the threshold of Z repression by BRAVO. Promoter activities in the stationary state are given by *pB*(*x*)=*P*_*B*_(*Z*_*s*_(*x*), *x*), *pZ*(*x*)=*P*_*Z*_(*W*_*s*_(*x*)) and *pW*(*x*)=*γ*_*QC*_(*x*), where again the subscript s indicates that the concentration profiles are those corresponding to the stationary state.

### Mixed model

This model combines the immobilization by sequestration with intermediary Z and the repression models. It reads:
(10)

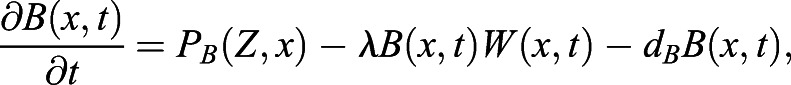

(11)

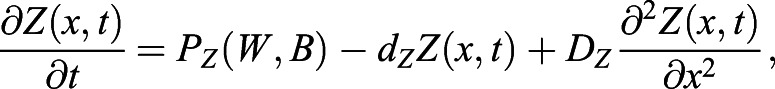

(12)

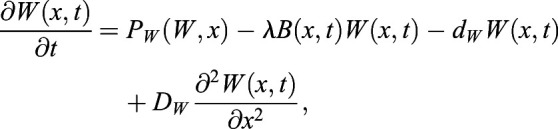
with the production terms 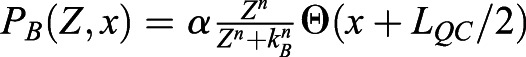, 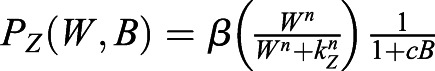 and 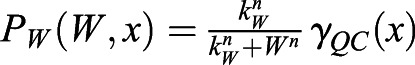. When basal activation of BRAVO at the VI regions is included (Fig. S7C), we use 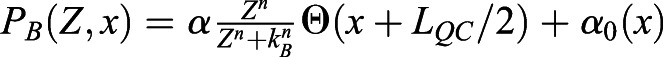, as done for the immobilization model. The stationary promoter activities (expressions) of BRAVO, WOX5 and Z are defined as *pB*(*x*)=*P*_*B*_(*Z*_*s*_(*x*), *x*), *pW*(*x*)=*P*_*W*_(*W*_*s*_(*x*), *x*) and *pZ*(*x*)=*P*_*Z*_(*W*_*s*_(*x*), *B*_*s*_(*x*)), respectively, where the subscript s denotes the protein profile in the stationary state.

### Modeling *bravo* mutants

The same type of approach as in Betegón-Putze et al. (2021) is used to simulate the *bravo* mutant. Specifically, to model this mutant, the very same dynamical equations and the same parameter values are used as those to model the WT (as described in the models above), except for BRAVO, which is set as *B*(*x*, *t*)=0 for all *x* and *t*. This leads to stationary values of *W*_*S*_(*x*) and *Z*_*S*_(*x*) that are different than in the WT. Although no dynamical equation for *B*(*x*, *t*) is set, there is a promoter activity of BRAVO in the stationary state, *pB*(*x*), which is as defined for the WT but with the stationary profiles of the mutant. We exemplify this with the ‘immobilization by sequestration’ model defined by Eqns 1 and 2. Setting *B*(*x*, *t*)=0 into Eqns 1 and 2, we get:
(13)

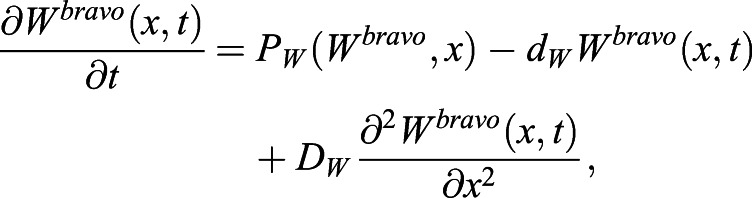
where the new superscript *bravo* indicates that the model corresponds to the *bravo* mutant and *P*_*W*_(*W*^*bravo*^, *x*) is the same function as the one used for the WT, but using the variable *W*^*bravo*^ instead of *W*. The stationary *BRAVO* promoter is 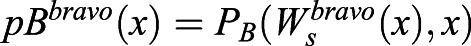, where 
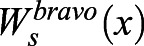
 is the stationary solution of Eqn 13. The same procedure is applied for the other models.

### Parameter space exploration

Parameter space exploration was done for the models above in one spatial (1D) dimension. For simplicity, in this 1D geometry, the VI region (e.g. light gray area in Fig. 2B-E, Fig. 3B-D, Fig. 4B-F, Fig. 5B,C) was defined to be of the same size as the QC region 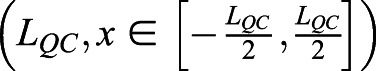. Hence, the end of the VI is at position *x*_*VI*_=3*L*_*QC*_/2. To assess whether the simulation results are compatible with experimental observations, we considered four different observables, *ξ*, *R*_*B*_, *R*_*BW*_ and *R*_*W*_, defined as follows:

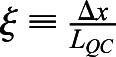
 quantifies the expansion of the stationary *BRAVO* promoter expression in the *bravo* mutant compared with the WT, Δ*x*, relative to *L*_*QC*_ (QC size), i.e. *ξ*=1 means that the stationary *BRAVO* promoter expression in the *bravo* mutant is expanded to a region as large as the QC compared with that in the WT. To define Δ*x* (Fig. 2C) we compute the value of the stationary *BRAVO* promoter expression in the WT at the end of the VI region and define this value as 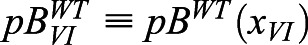. We then compute the spatial position at which the stationary *BRAVO* promoter expression in the *bravo* mutant takes this value, and define this position as *x*^*bravo*^ (i.e. it is defined as 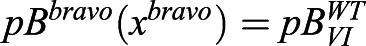). Δ*x* is defined as Δ*x*≡*x*^*bravo*^−*x*_*VI*_. Because of our simplified one-dimensional geometry, *ξ* values are not expected to be equal to those in real roots.
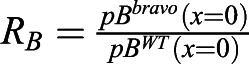
 is the ratio of stationary *BRAVO* promoter expression at the center of the QC in the *bravo* mutant over that in the WT. Because the twofold change from experimental data (Fig. 1F) corresponds to the increase of the spatially integrated GFP expression across the midplane, and thus involves the increase at the QC as well as the increase (and thereby expansion) in other regions, we considered that *R*_*B*_ values consistent with experimental observations range between 1 and 2.
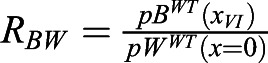
 is the ratio of stationary *BRAVO* promoter expression at the end of the VI over stationary *WOX5* promoter expression at the center of the QC, both in the WT.
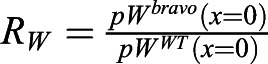
 is the ratio of *WOX5* promoter expression at the QC in the *bravo* mutant over that in the WT. This observable is only used when the models include the (indirect) action of BRAVO on the *WOX5* promoter, i.e. when there is WOX5 self-repression and BRAVO-WOX5 sequestration.

We compute these four observables in distinct parameter planes by solving, for each model for the WT and the *bravo* mutant, the corresponding equations in time, until the stationary state is reached (see simulation details in the ‘Numerical implementation of the mathematical models’ subsection below). Each parameter space in the logarithmic scale is discretized into a regular rectangular lattice of 15×15 pairs of values, for which ξ, *R_BW_*, *R_B_* and *R_W_* are evaluated. Heatmaps are generated using the Python contourf function for each observable. The orange domains correspond to those regions where all observables meet the conditions compatible with experimental observations.

### Construction of realistic root layouts

In order to build two-dimensional realistic root layouts with which we could subsequently implement the corresponding reaction-diffusion equations (e.g. the mixed model in next subsection), we started by taking a confocal image of a middle plane of the root with propidium iodide-stained cell walls and we applied a segmentation routine that divides the root at the pixel scale and into its constituent cellular regions and cell walls (Fig. S8). To do this, we made extensive use of the *scikit-image* collection of Python-based algorithms (*threshold_otsu*, *skeletonize* and *label*) (van der Walt et al., 2014). In particular, we first defined the cell boundaries with the *threshold_otsu* method, which transforms the original image into a thresholded binary image in which only cell wall pixels remain. We then used *skeletonize* to obtain a cell wall with a fixed width (2 pixels). We then applied the *label* function on the modified image to label distinct regions (each label constitutes the collection of pixels belonging to the particular region). We chose to define labels that enable us to distinguish between cells and between the cell wall and the outside of the root as follows: all pixels within a cell have the same label, which is distinct from that of pixels in any other cell. With this routine, we could access each cell as an individual entity, and we could modify its properties as a whole (e.g. change the parameter values of the protein dynamics in all pixels of that cell). A single label is assigned to all the pixels in any cell wall. Thus, all cell walls constitute a single, the same, entity. Another single region is defined by all pixels outside of the root.

The pixel grid is the spatial grid on which the dynamical equations of protein concentrations are set. For the images used, 1 pixel 
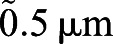
. We assigned the same dynamical equations and parameter values in all pixels within each labeled region. These equations and parameter values can be distinct between regions. Specifically, the equations applied on the cell wall are distinct from those within cells, as described in the next subsection. The diffusion coefficient of a protein within all pixels of the cell wall is the same. For simplicity, the diffusion coefficient of a protein is set to be the same in all the cell regions (i.e. within any cell), but distinct from that in the cell wall. The only differences set between different cells are in the protein production terms. Further details on the construction and implementation of the model in the realistic root layouts can be found in the supplementary Materials and Methods.

### Mixed model in a realistic root layout

The equations for the rate of change of the concentrations of each type of protein across space 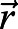
and time *t* are:
(14)

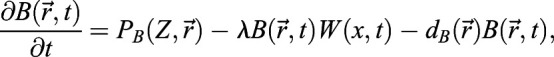

(15)

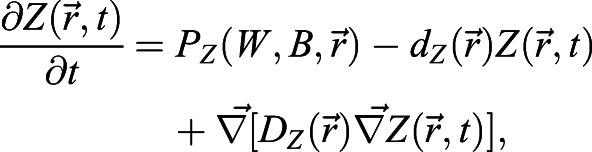

(16)

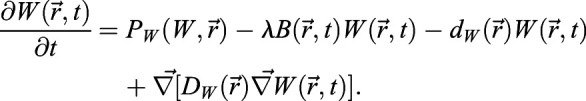


The GFP proteins produced under the promoters of *BRAVO* (*B*_*GFP*_), of *WOX5* (*W*_*GFP*_) and of *Z* (*Z*_*GFP*_) have the same production rate as BRAVO, WOX5 and Z, respectively. All these GFP proteins have the same diffusion coefficient and all degrade linearly with the same degradation rate (i.e. that of GFP, *d*_*GFP*_). Hence, the equations for the dynamics of these GFP proteins are:
(17)

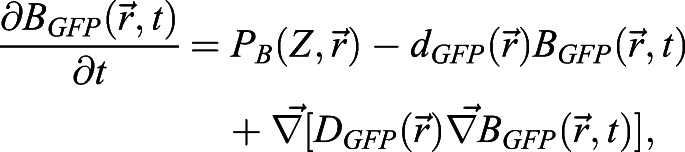

(18)

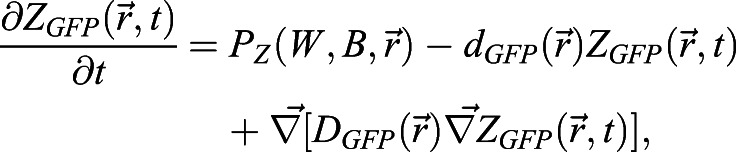

(19)

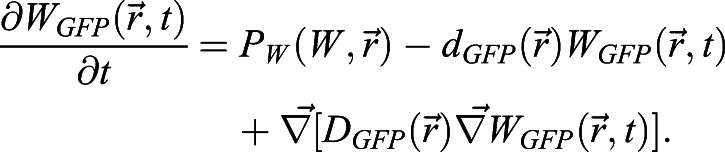


In all these equations, production and degradation only happens in pixels labeled as cells. Within cells, the production functions 
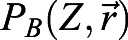
, 
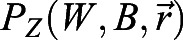
 and 
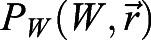
 depend on *Z*, *B* and *W* as in the one-dimensional mixed model with basal production of BRAVO, i.e. 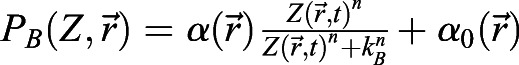, 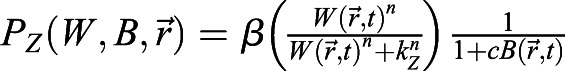 and 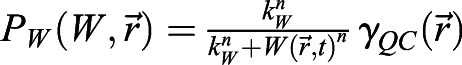. In these expressions, 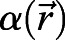 takes the constant value *α* only in the cells shown in Fig. S9, being zero in all the other cells and regions; 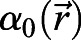 takes the constant value *α*_0_ only in the pixels corresponding to the cells shown in Fig. S10, being zero in all other regions; 
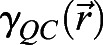
 takes the constant value *γ* only in QC cells (Fig. S9), being zero in all the other cells and regions.

We impose the restriction that inside cell walls only diffusion can happen, whereas no reaction (production, degradation or binding) can occur. Hence,

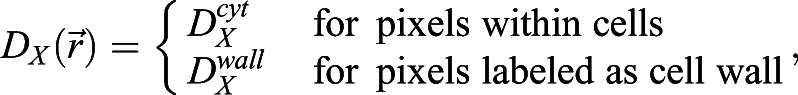


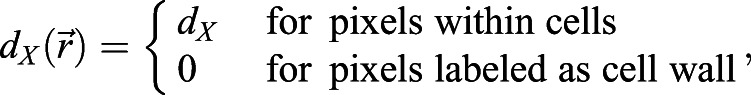


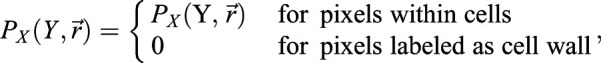
where X stands for any of the modeled species and Y for those species that regulate the production of X. The diffusion coefficient in the cell wall (denoted by superscript *wall*) is distinct from that inside cells (denoted by superscript *cyt*). No differences between cells are set on degradation rates or diffusion coefficients.

All equations described up to this point correspond to the WT condition. To model the *bravo* mutant, the same equations with the same parameter values are used, but the BRAVO protein concentration is set to zero (i.e. the mutant corresponds to Eqns 15-19 and 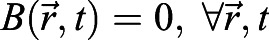.) Note that to model this mutant, the GFP reporter for *BRAVO* promoter, 
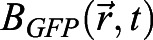
, is described with the same equations as in the WT (i.e. with Eqn 17) and hence is not set to zero.

In Fig. 6 and Figs S11-S14, *pBRAVO:GFP* corresponds to the variable 
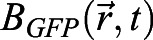
computed at the stationary state (i.e. the activity of the *BRAVO* promoter as seen through its GFP reporter), whereas 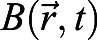 represents the stationary BRAVO protein concentration. Analogous definitions for WOX5 and Z are used. Fig. 6B-D use the same parameter values as in Fig. 6A (Table S3) except for *c*=0 and 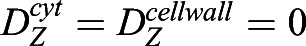 (Fig. 6B); *c*=0 (Fig. 6C); and *λ*=0 (Fig. 6D).

### Numerical implementation of the mathematical models

To find the stationary spatial profiles of the 1D models (‘immobilization by sequestration’, ‘immobilization by sequestration with intermediary Z’, ‘repression’ and mixed models), we first reduced the system of differential equations to second-order ordinary differential equations for the diffusible variables. To do this, we first set to zero the equation for the non-diffusible variables and substituted the result on the other equations. The remaining system could be cast as a boundary value problem, which we solved numerically by using the solve_bvp routine embedded in the Python-based *SciPy* library (Virtanen et al., 2020). In all these calculations, we used a spatial step size *δx*=0.05 a.u. (arbitrary units), in a domain of *x*∈[−600, 600]a.u.. We use a QC size of *L*_*QC*_=15 a.u., and an equally long VI region. With this methodology, the explicit time dependence of the variables is not computed, but only their stationary state. To double-check the validity of our solutions, we also simulated the whole temporal dynamics of the equations with a forward Euler method, obtaining the same stationary solutions as with the solve_bvp method, thus confirming the results (Figs S1-S7). For all the models, the values of *B*, *W* and *Z* at the boundaries (*x*=600 and *x*=− 600) are set to zero, except for the additional sequestrator variable *S* (Fig. S3), for which periodic boundary conditions are implemented.

The results displayed on the heatmaps were obtained by simulating the dynamical equations corresponding to each model for the WT and the *bravo* mutant scenarios until the stationary state was reached. To compute the temporal dynamics, we used a forward Euler method and discretized the 1D Laplacian term as 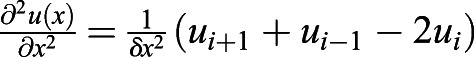, where *u*(*x*) represents either *W* and/or *Z*. The spatial step is *δx*=1 and uses a domain *x*∈[−400, 400]a.u.. The time step is dt=0.01 and the final time is tfinal=1000, where the steady state is reached. All variables are set to zero as the initial conditions and all variables are set to zero at the boundaries. The stationary profiles in Figs S1-S7 were computed with the Euler algorithm.

To simulate the dynamics of the mixed model (and its modifications) in the realistic root layout, we solved the corresponding reaction-diffusion equations with heterogeneous diffusion coefficients with a forward-time central-space scheme (FTCS), in which time is discretized in steps of size Δ*t*=0.1 a.u. (so that after k steps, time is *t*_*k*_=*k*Δ*t*) and space is discretized in steps of size (*δx*=1, *δy*=1) pixels, so that (*x*_*i*_, *y*_*j*_)=(*iδx*, *jδy*), with a lattice size of (*L*_*x*_, *L*_*y*_)=(228, 448) pixels for the WT root and (*L*_*x*_, *L*_*y*_)=(231, 448) pixels for the *bravo* mutant root. For the images used, 1 pixel≈0.5 μm.

We ran the simulations up to time *t*=3000 a.u., (Fig. 6; Figs S11-S14) this time point can be taken to correspond to the stationary state. All the diffusion coefficients outside the root layout are set to be zero, restricting the domain of the equations to the interior of the root. Additionally, this condition automatically implements reflecting boundary conditions at the root borders.

## Supplementary Material

Supplementary information

Reviewer comments
