# Supplementary material for: BRAVO self-confined expression through WOX5 in the Arabidopsis root stem-cell niche
Source: Development. 2022 Jul 28;149(15):dev200510. doi: 10.1242/dev.200510 (PMC9440757; doi:10.1242/dev.200510)
Supplement: Supplementary information [file develop-149-200510-s1.pdf]

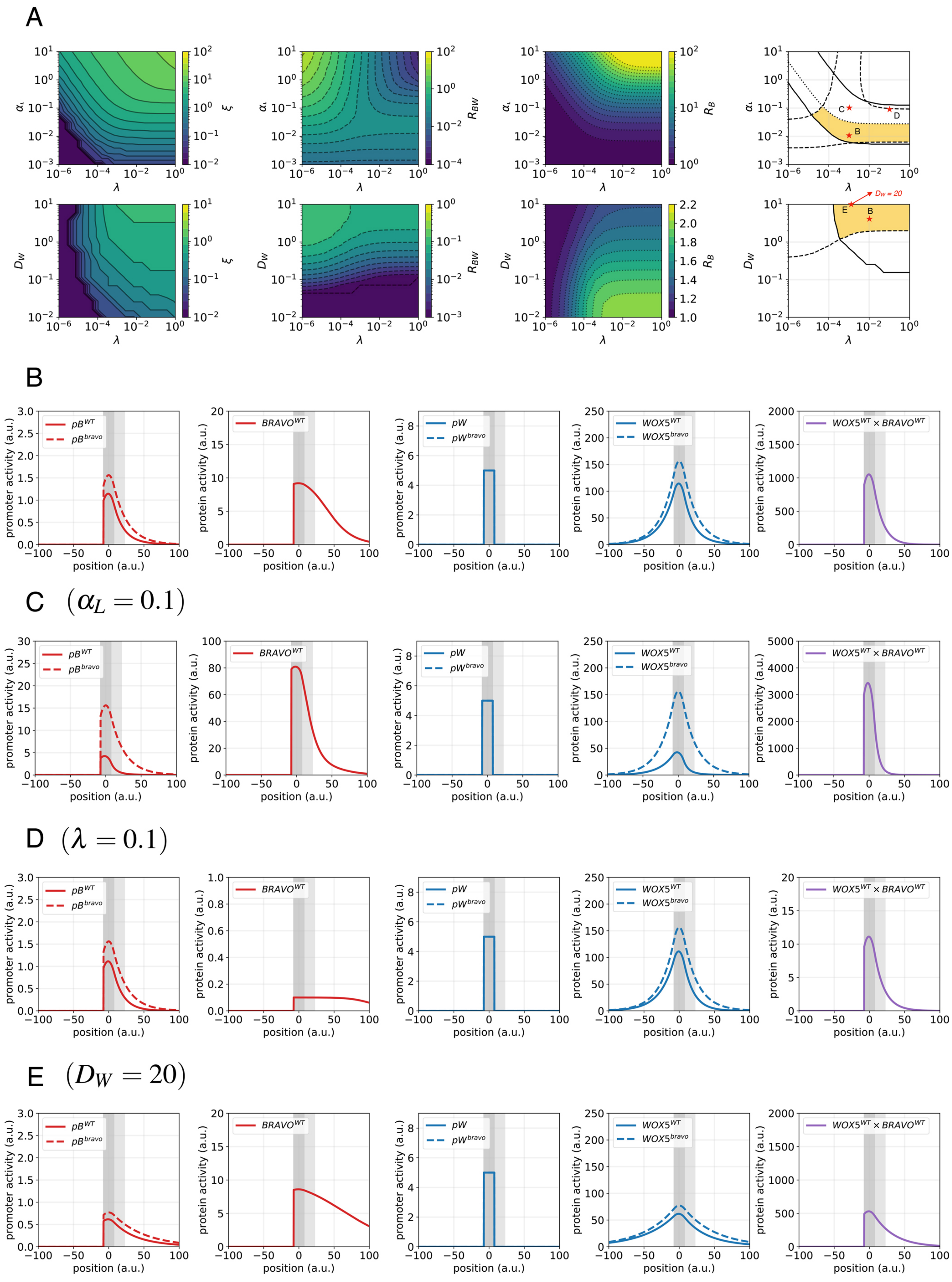

Development • Supplementary information

Fig. S1. Parameter exploration for the Immobilization by sequestration model with linear regulations.

(A) Heatmaps and contour lines showing the values of ξ, R_{BW}, and R_B in the α_L - λ (top) and D_W - λ (bottom) planes. In the rightmost panels, the coloured orange areas enclose the parameter regions fulfilling the conditions $0.05 \leq \xi \leq 5$ (continuous curves), $0.05 \leq R_{BW} \leq 0.5$ (dashed curves) and $1 \leq R_B \leq 2$ (dotted curves). These rightmost panels are the same as those in Fig. 2F,G and are repeated here to facilitate the understanding of how they are related to the heatmaps of each observable. To induce an expansion of *pB(x)* in the *bravo* mutant consistent with experimental results, sufficiently large values of complex formation and WOX5 diffusion are needed. Stars labelled by the letters B, C, D, and E point to the parameter pairs corresponding to the profiles shown in panels B, C, D, and E, respectively. Rest of the parameters as in Table S1. (B) Stationary profiles of *pB(x)*, *B(x)*, *pW(x)*, *W(x)*, and the product *B(x)W(x)* in the WT (continuous lines) and in the *bravo* mutant (dashed lines) for the same parameter values as in Fig. 2B-E. *pB(x), B(x), pW(x),* and *W(x)* are the same as those in Fig. 2C, D, and E, respectively, and are repeated here for better comparison to *B(x)W(x)* and panels C-E. (C-E) Analogous to B, for the same parameter values except for one: (C) $\alpha_L = 0.1$, (D) $\lambda = 0.1$ and (E) $D_W = 20$. When compared to B, C-E show the effect of higher BRAVO production, stronger binding or higher WOX5 diffusion respectively. For a higher production rate (C), *pB* in the WT is mostly confined at the QC (with similar levels to *pW*) and nearly absent in the VI. Such strong confinement is not compatible with real expressions in *Arabidopsis* roots. Higher complex formation (D) has a strong impact on the levels of free BRAVO, which are very small due to higher sequestration by WOX5. For higher WOX5 diffusion (E), the concentration of WOX5 proteins remains relatively high across a very broad region beyond the VI. Consequently, *pB(x)* is activated in such a large region.

Development • Supplementary information

A. Non-linear (n=1)

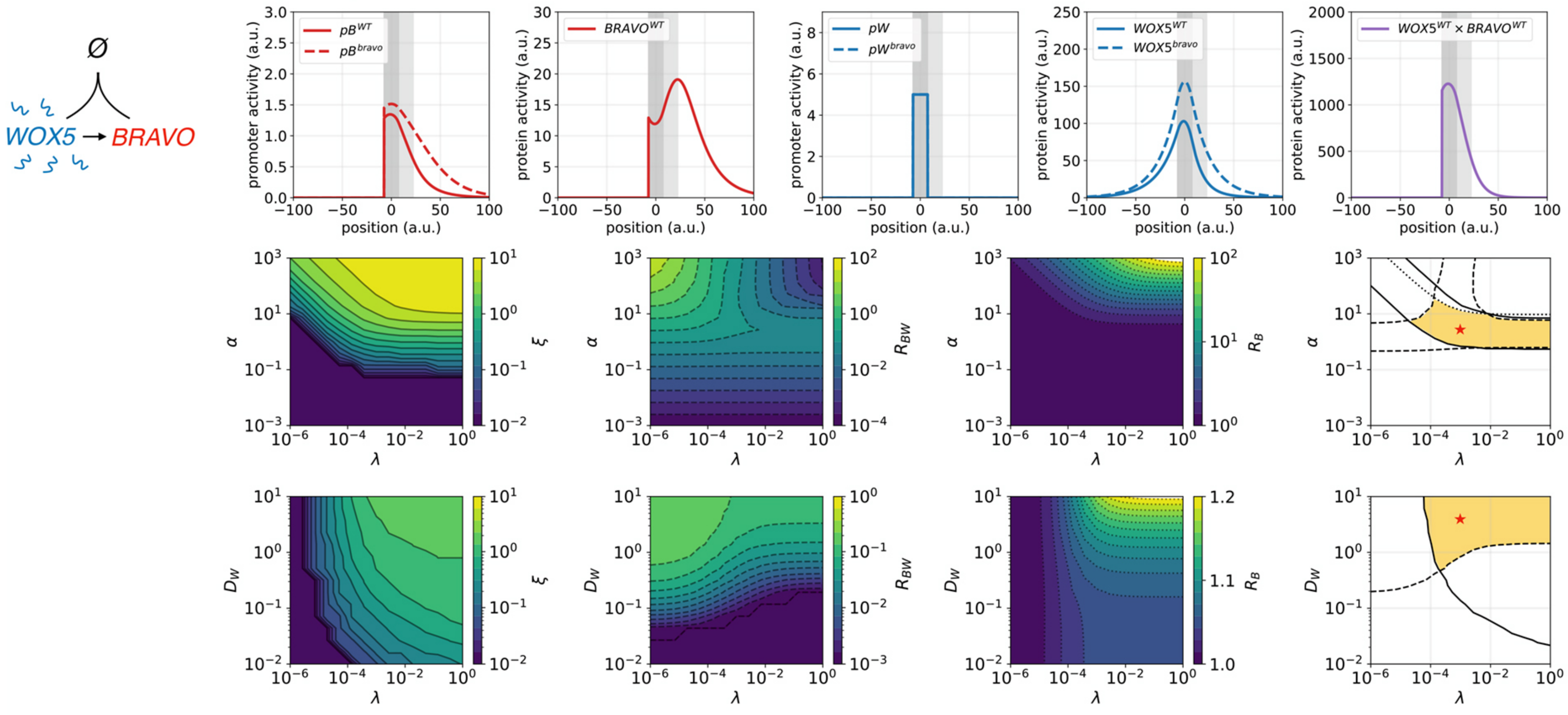

B. Non-linear (n=3)

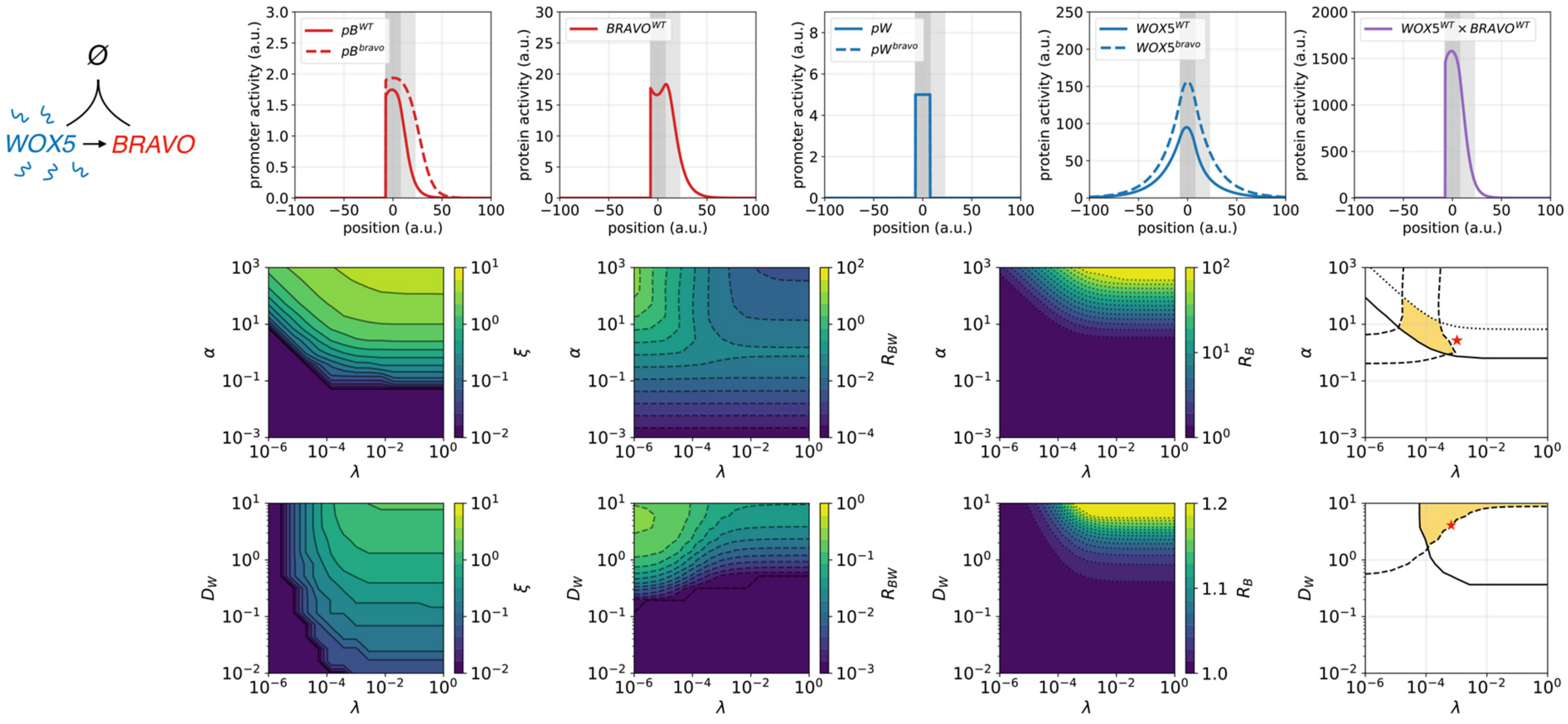

C. Non-linear (n=1) and WOX5 self-repression

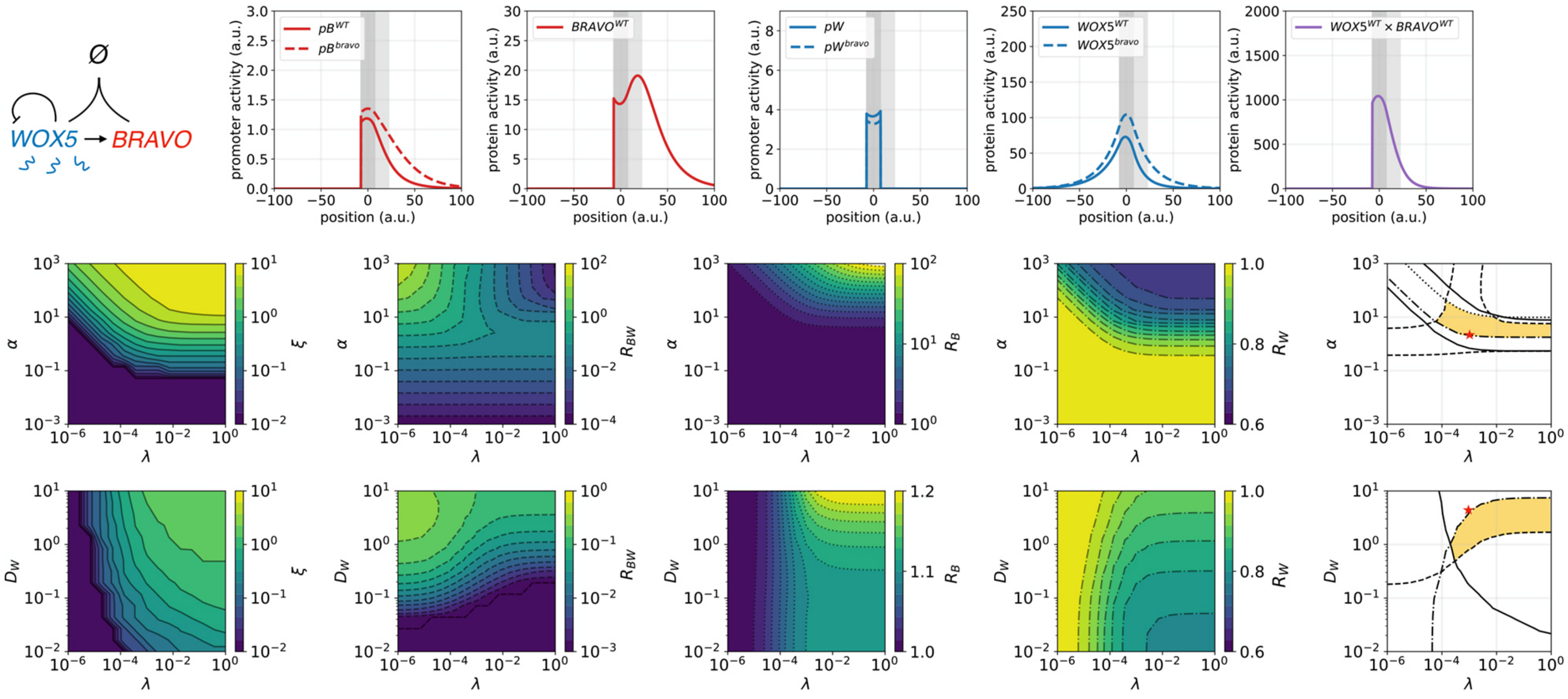

Development • Supplementary information

Fig. S2. Immobilization by sequestration model with non-linear regulations and WOX5 self-repression.

In this figure the results of the immobilization model when we allow the production terms to be non-linear are shown for: (A) all cooperativity exponents $n = 1$; (B) All cooperativity exponents $n = 3;$ (C) All cooperativity exponents $n = 1$ and WOX5 self-repression added. Rest of the parameters as in Table S1. Each panel contains: (top) The stationary profiles of $pB(x)$, $B(x)$, $pW(x)$, $W(x)$ and the product $B(x)W(x)$ in the WT (continuous lines) and in the *bravo* mutant (dashed lines); (middle and bottom) Heatmaps and contour lines depicting the values of ξ, R_{BW}, and R_B in the α_L - λ (middle) and D_W - λ (bottom) planes. Rightmost panels show the parameter regions fulfilling the conditions $0.05 \leq \xi \leq 5$ (continuous curves), $0.05 \leq R_{BW} \leq 0.5$ (dashed curves) and $1 < R_B < 2$ (dotted curves). Panel C also includes results on the fourth observable R_W. In A, we find that the valid parameter regions (orange) are similar to those of the linear model (Fig. S1A), considering that the parameter α in the non-linear model has units of [concentration/time]. In B, the results show that increasing the n exponent reduces the parameter regions where the three conditions for ξ, R_{BW}, and R_B are fulfilled, particularly because in this case the profile of $pB(x)$ decays too abruptly compared to the case with $n = 1$ (a restriction encoded in the quantity R_{BW}, dashed curves). In C, when WOX5 self-repression is introduced, the QC activity of the WOX5 promoter in the *bravo* mutant decreases with respect to the WT, and this is quantified by observable R_W. This imposes a further condition to the valid parameter regions, namely $0.5 \leq R_W \leq 0.9$. The dot-dashed curves in the rightmost panels represent the contour line $R_W = 0.9$. Therefore, introducing this new regulation and its corresponding condition slightly reduces the valid parameter regions.

Development • Supplementary information

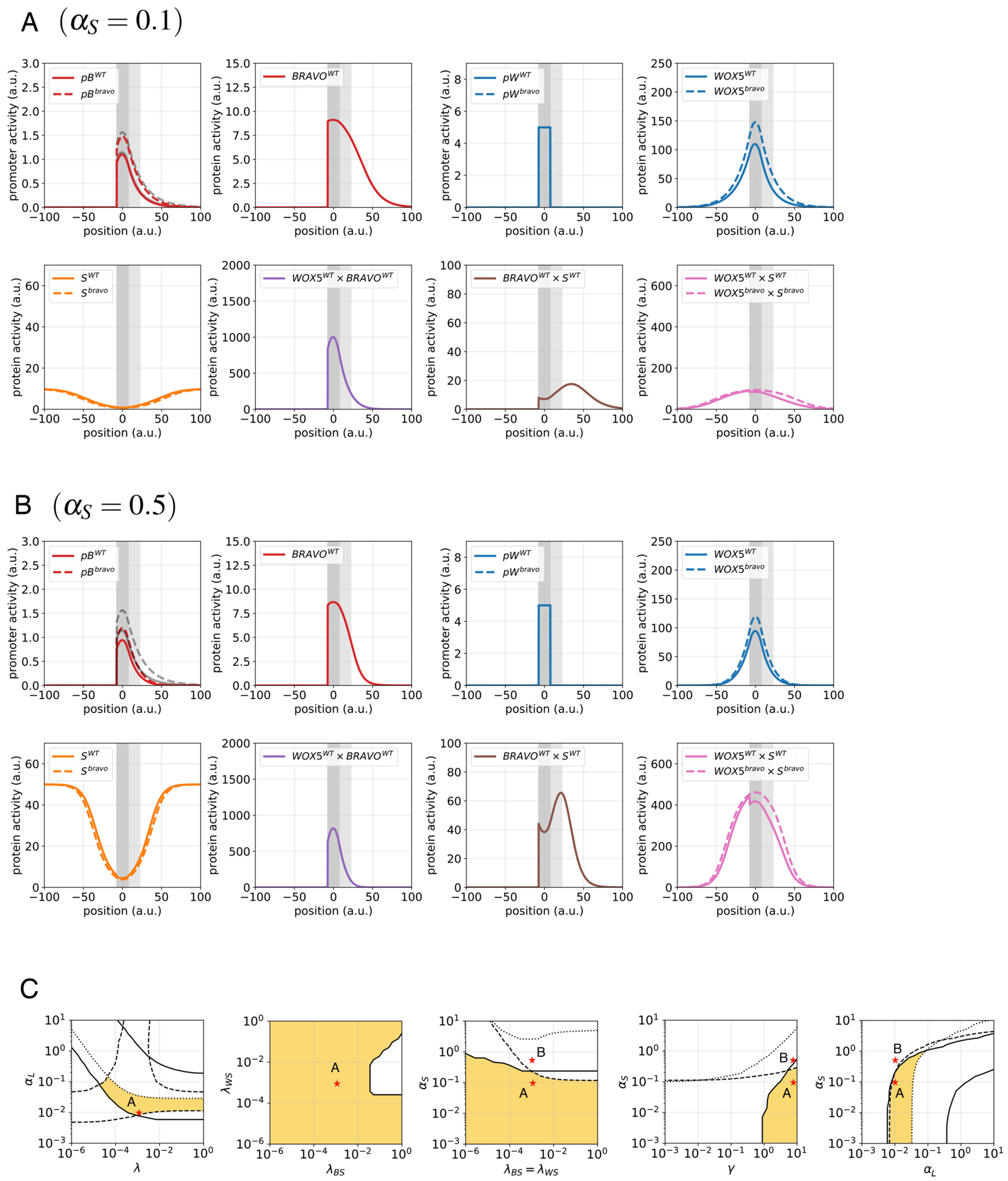

Development • Supplementary information

Fig. S3. Immobilization by sequestration model with an additional sequestrator *S*.

(A, B) Stationary profiles of *pB(x)*, *B(x)*, *pW(x)*, *W(x)* and *S(x)* for two different values of the production of the additional sequestrator, $\alpha_S = 0.1$ (A) and $\alpha_S = 0.5$ (B). At the QC, the rate of production of *B*, *W* and S are $\alpha_L W$, γ, and α_S, respectively (hence, the units of α_L are different from those of γ and α_S). The binding rates between *S* and BRAVO and between *S* and WOX5 are the same as those between BRAVO and WOX5, $\lambda_{BS} = \lambda_{WS} = \lambda_{WB} = 0.001$. The grey profiles plotted in *pB(x)* correspond to $\alpha_S = 0$ (i.e., the same as those in Fig. 2C), and are here plotted to be compared with the red profiles (obtained for $\alpha_S = 0.1$ (A) and $\alpha_S = 0.5$ (B)). The profiles of quantities proportional to the amount of each type of complex (i.e., the product of the unbound proteins) are also plotted. For $\alpha_S = 0.5$, S takes the role of BRAVO in the *bravo* mutant at sequestering WOX5. Notice that the *pB* profile in the *bravo* mutant when S is present (dashed red line) is like that in the WT when there is no S (continuous grey line). (C) As expected, the parameter regions where the expansion of the BRAVO promoter is within the conditions we deem valid decrease in size as α_S gets larger. Red stars mark the parameter pairs shown in A and B. The rest of the parameters are shown in Table S2.

Development • Supplementary information

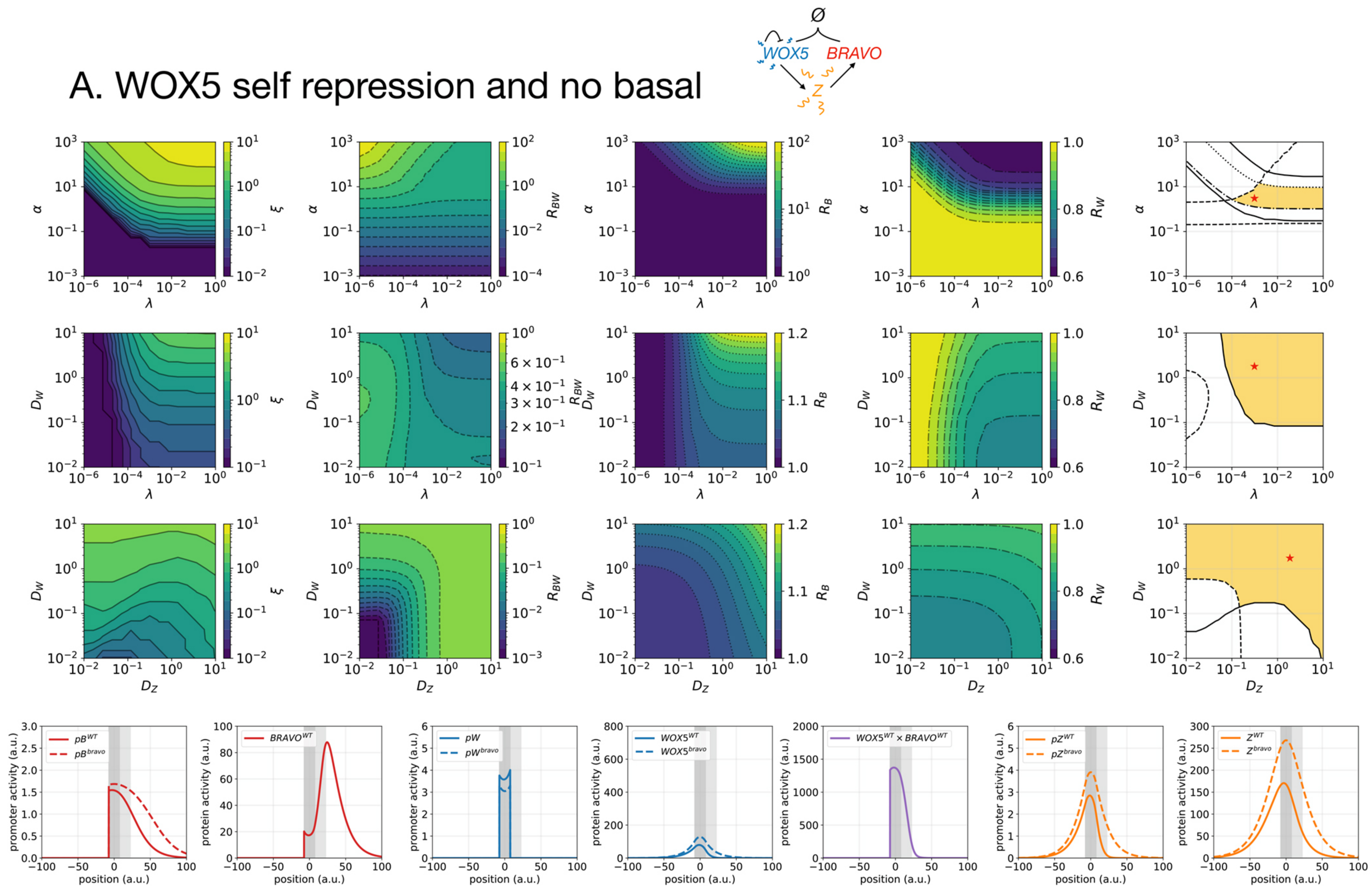

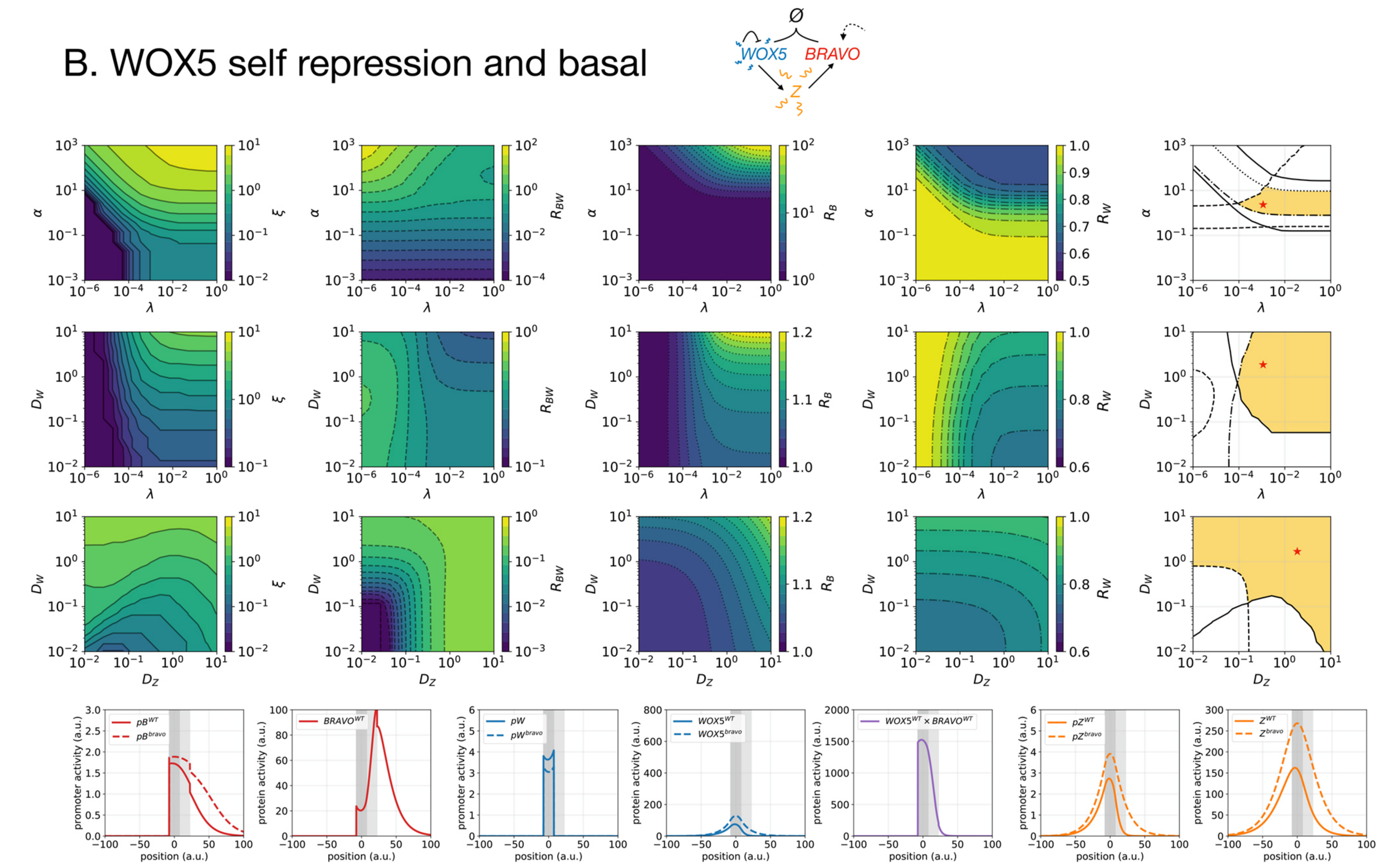

Development • Supplementary information

Figure S4. Immobilization by sequestration with an intermediary.

(A) Heatmaps and contour lines showing the values of ξ, R_{BW}, R_B and R_W for the immobilization by sequestration model when a diffusible intermediary Z is present, in the parameter planes α - λ, D_W - λ and D_W - D_Z. The rightmost panels depict the valid (i.e., compatible with experimental data) parameter domains in each plane. The D_W - λ and D_W - D_Z plots depicting the valid domains are the same as those in Fig.3E,F and are here repeated for completeness. Stationary spatial profiles (for all molecular components and the $B*W$ product) shown below correspond to the parameter values marked by a red star. In the α - λ plane, the valid (orange) regions are similar to the immobilization model without Z (Fig. S2A). In contrast, the valid region in the D_W - λ plane increases, particularly towards lower values of D_W, when compared to the model without Z (Fig. S2A). This happens because for small or absent WOX5 diffusion, the diffusion of Z can take its place as the mobile factor. This is evidenced in the D_W - D_Z plane, where very small WOX5 diffusion can be compensated by that of Z. The rest of the parameters as in Table S1. (B) Analogous to A but when a basal BRAVO production in the VI region (with rate $\alpha_0 = 0.2$) is introduced. As can be seen, introducing a basal activity of BRAVO does not introduce important changes to the valid parameter regions.

Development • Supplementary information

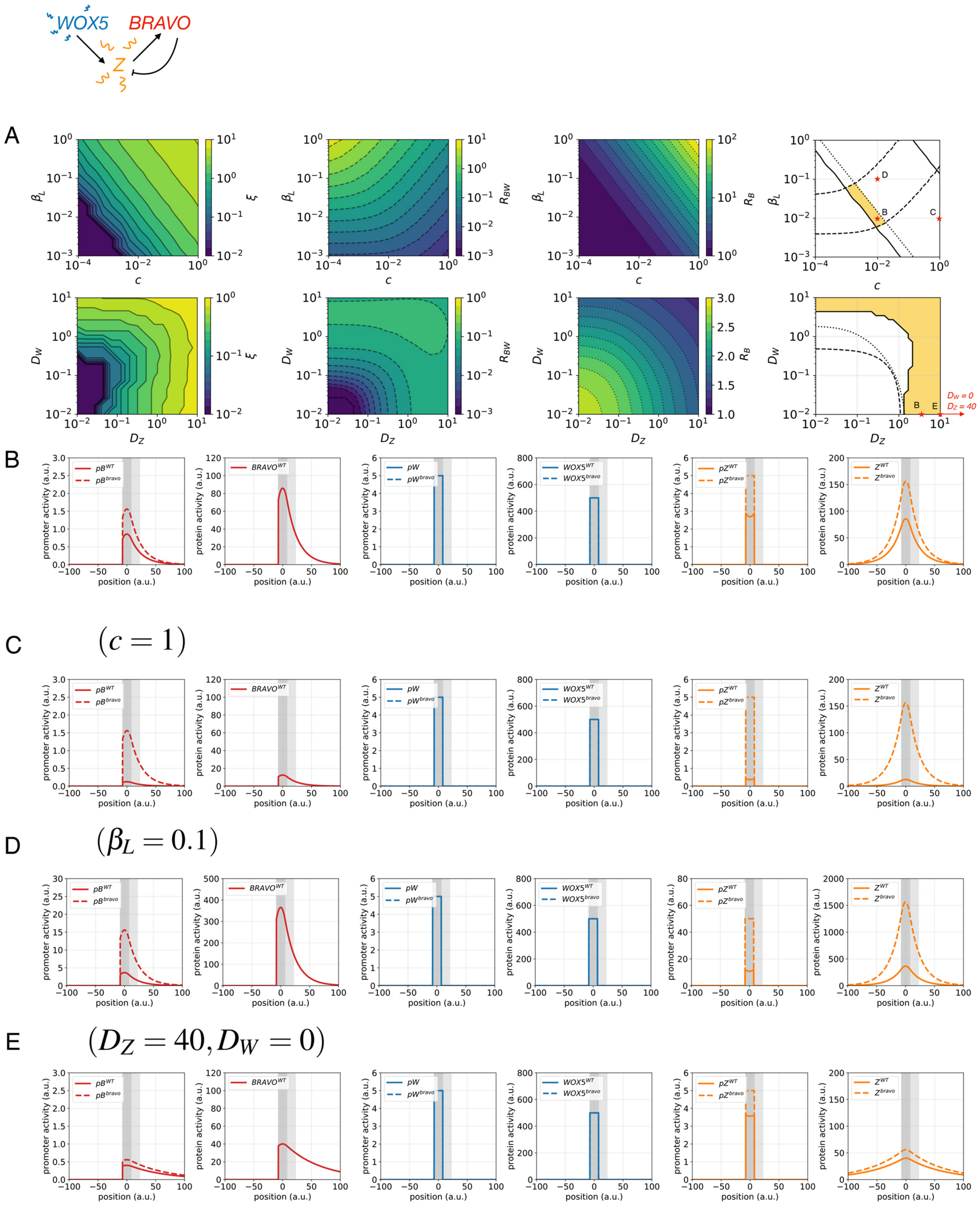

Development • Supplementary information

Figure S5. Parameter exploration for the Repression model with linear productions.

(A)Heatmaps and contour lines showing the values of ξ, R_{BW}, and R_B in the β_L - c and D_W - D_Z planes, corresponding to the repression model when productions are linear functions (Methods). The rightmost panels depict the valid (i.e. compatible with experimental data) parameter domains in each plane. The D_W - D_Z plot depicting the valid domain is the same as that in Fig.4G and is here repeated for completeness. As shown in the rightmost panels, only a small region of β_L - c values enable a valid domain: a spatial expansion of *BRAVO* expression in the *bravo* mutant large enough ($\xi \geq 0.5$, continous lines) but with small increase of *BRAVO* expression at the QC ($R_B \leq 2$, dotted line). Moreover, either (large) diffusion of WOX5 or diffusion of Z is needed, and one can compensate the diffusion of the other. Stars labelled by the letters B, C, D and E point to the parameter values corresponding to the stationary spatial profiles shown in panels B, C, D and E, respectively. In the D_W - D_Z plane, the starts are depicted at the bottom value of D_W but results in B and E correspond to D_W=0. Parameters as in Table S1. (B-E) Stationary profiles of *pB(x)*, *B(x)*, *pW(x)*, *W(x), pZ(x)* and Z*(x)* in the WT (continuous lines) and in the *bravo* mutant (dashed lines), for the same parameter values as in Figure 4C-F except for one: (C) $c = 1$; (D) $\beta_L = 0.1$; and (E) $D_Z = 40$. The panels in B are the same as those in Figure 4C-F and are here repeated for better comparison to C-E. Accordingly, results in C, D and E, when compared to B, depict the effect of higher BRAVO repression to Z, higher production of Z and higher diffusion of Z, respectively. In C, the higher repression strength drives very low *pB* in the WT, while it increases and spans very dramatically in the *bravo* mutant, which is not compatible with experimental data. In D, for this higher production rate of Z, *pB* in the WT is very high, and increases very dramatically in the *bravo* mutant, which is not compatible with experimental data. In E, for this higher Z diffusion coefficient, *pB* in the WT is fainter and more spanned.

Development • Supplementary information

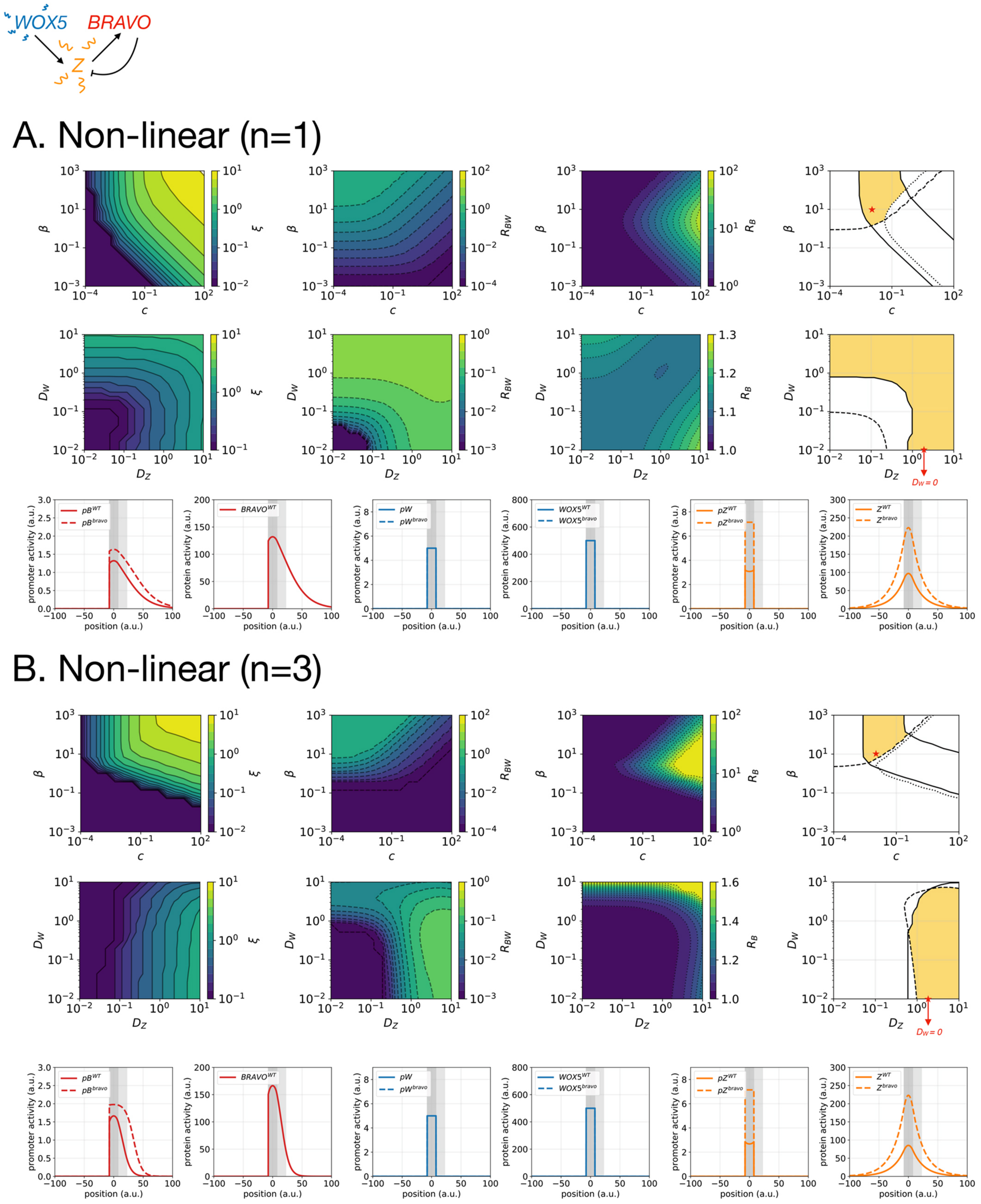

Development • Supplementary information

Figure S6. Repression model with non-linear regulations.

(A)Heatmaps and contour lines showing the values of ξ, R_{BW}, and R_B in the β - c and D_W - D_Z planes, corresponding to the repression model when regulations are nonlinear functions, using all exponent n=1 (Methods). The rightmost panels depict the valid (i.e., compatible with experimental data) parameter domains in each plane. The rightmost panel in the β - c plane is the same as Fig. 4H and is here repeated for completeness. For nonlinear regulations the valid parameter region in the β - c plane changes with respect to the linear model (Fig. S5A), being much larger now. In the D_W - D_Z plane the valid region for n=1 is similar to the linear case (Fig. S5A). Stationary spatial profiles (for all molecular components) shown below correspond to the parameter values marked by a red star. (B) Analogous to A but for all cooperativity exponents $n = 3$. Increasing this exponent reduces the parameter regions in the D_W - D_Z plane but no in the β - c plane. In particular, for this high cooperativity, the diffusion of Z becomes necessary, as WOX5 alone cannot induce an expansion large enough to be considered valid within our imposed conditions.

Development • Supplementary information

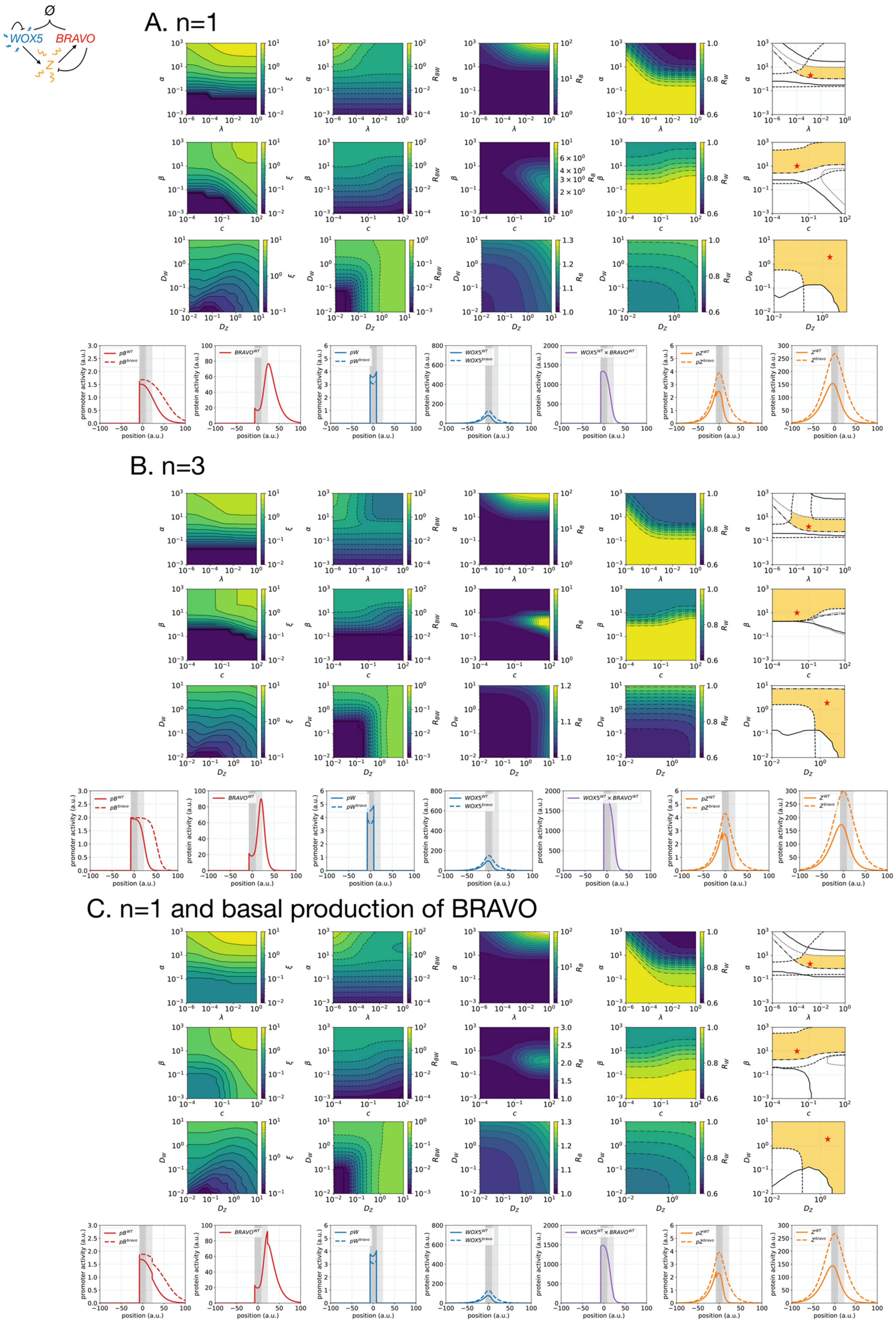

Development • Supplementary information

Figure S7. Mixed model with higher nonlinearities and basal BRAVO production.

(A)Heatmaps and contour lines showing the values of ξ, R_{BW}, R_B and R_W in the $\alpha - \lambda$ (top), β - c (middle) and D_W - D_Z (bottom) planes, corresponding to the mixed model (Figure 5). The rightmost panels depict the valid (i.e., compatible with experimental data) parameter domains in each plane. These are the same as those depicted in Fig. 5D-F and are repeated here for completeness. While in the α - λ the valid regions are similar to the immobilization by sequestration model (Fig. S4A), in the β - c plane we see that they extend to arbitrarily low values of c when compared to the results for the repression model (Fig. S6A). This happens because in the absence of repression, complex formation can still induce confinement of the BRAVO promoter. Red stars indicate the parameter pairs corresponding to the activity profiles shown below. The profiles of $pB(x)$ and $pW(x)$ shown here are the same as those shown in Fig 5C for c=0.01. The rest of the parameters as in Table S1. (B) Analogous to A but with cooperativity exponent $n = 3$. Increasing this exponent does not modify significantly the valid regions where the conditions on ξ, R_{BW}, R_B and R_W are fulfilled, despite the change in the spatial profile of the BRAVO promoter (there is a more abrupt decay). (C) Analogous to A, but with the introduction of a basal BRAVO production in the VI region (with rate $\alpha_0 = 0.2$). As found for the immobilization by sequestration model (Fig. S4), adding a basal activity of BRAVO does not introduce important changes to the valid parameter regions.

Development • Supplementary information

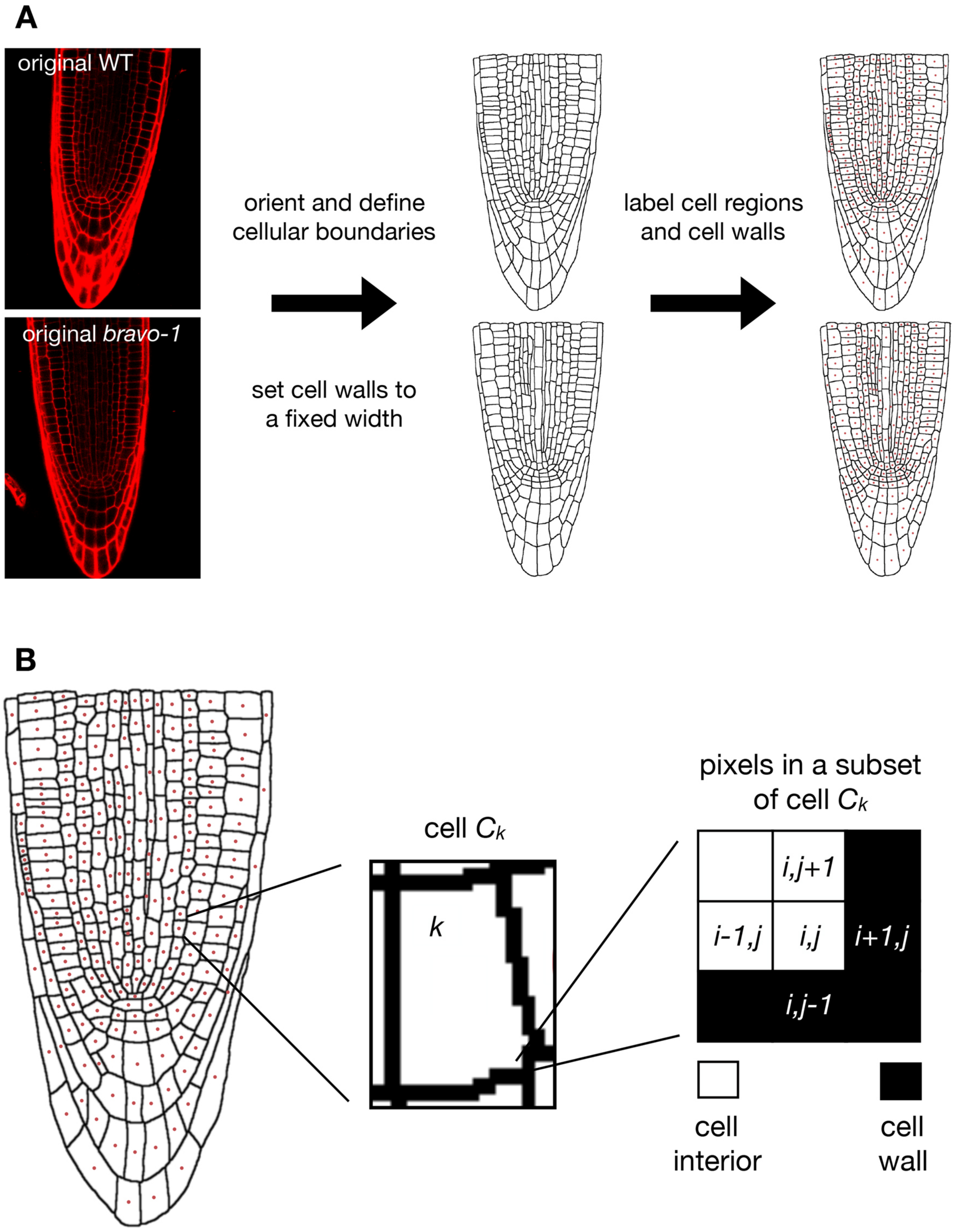

Fig. S8. Construction of a realistic root layout.

(A) We select two root tips, representative for WT and *bravo-2* mutants, from a confocal image of the root where cell walls are PI-stained (red). We first orient and re-scale the roots so that both can be compared. As a result, proportions are slightly modified from the original image. This initial step is optional. We then reset a fixed width for the cell walls (2 pixels in the simulation). Subsequently, we label the images to define each cellular region (marked as red dots located at the centroid of each cell). (B) Cell C_k is the cell that contains the pixels with label k (which define the cell's interior, white). It is surrounded by pixels corresponding to cell walls (black). The spatial position of a pixel is denoted by two indexes, i and j. On this discretized grid we implement the corresponding reaction-diffusion equations.

Development • Supplementary information

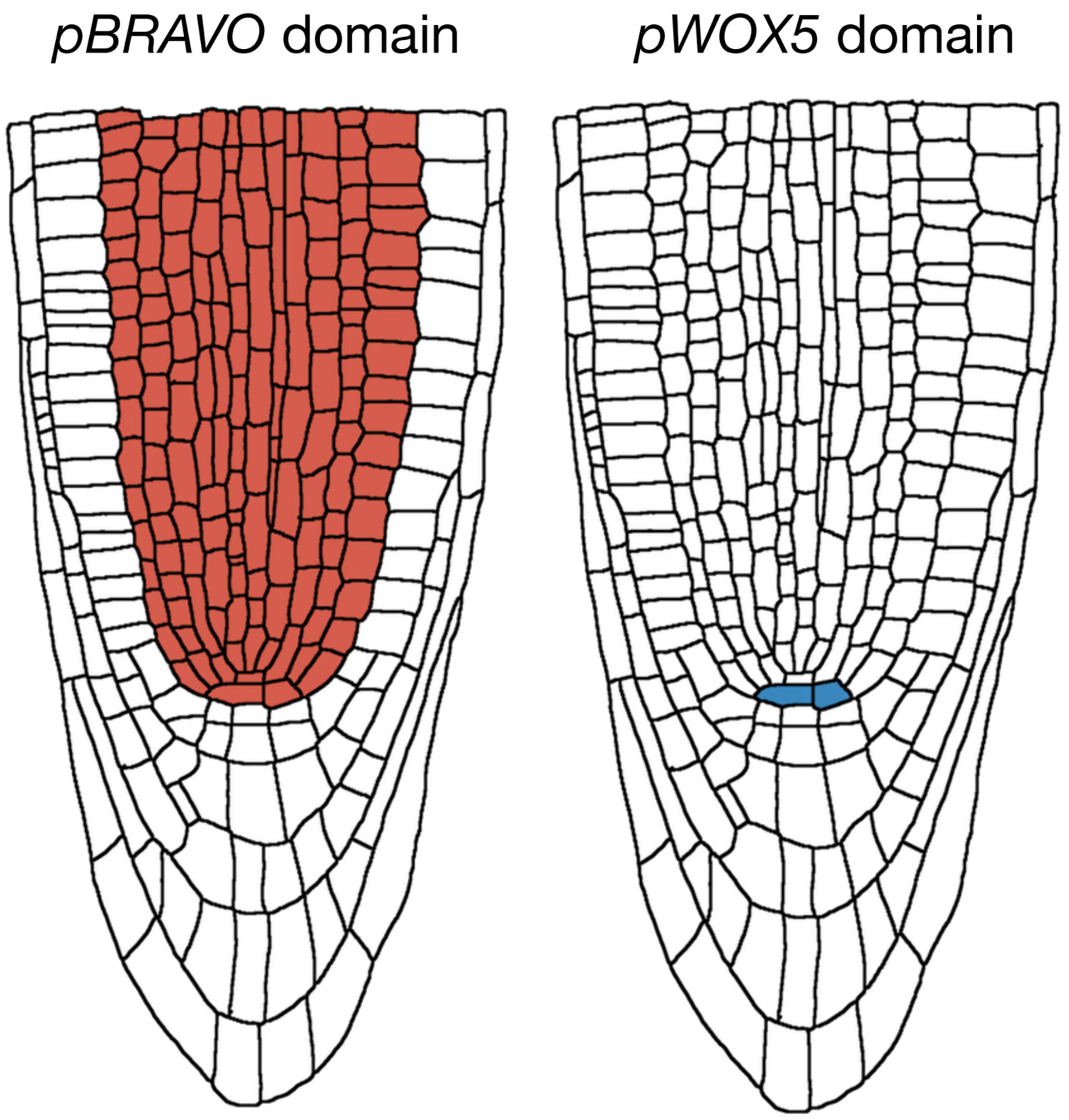

Fig. S9. Cellular domains where production of BRAVO and WOX5 is enabled in the simulations.

The production terms of BRAVO and WOX5 are restricted to the red and blue domains respectively (these regions are denoted as the *pBRAVO* and *pWOX5* domains, since they indicate where the promoters BRAVO and WOX5 are active). The GFP reporters of their promoters are produced only in these domains. In this way, we only allow B and B_{GFP} to be activated by Z in the QC, vasculature, cortex and endodermis, while W and W_{GFP} are only produced at the QC. This does not prevent WOX5 and GFP proteins to be present in other tissues, which can reach through diffusion.

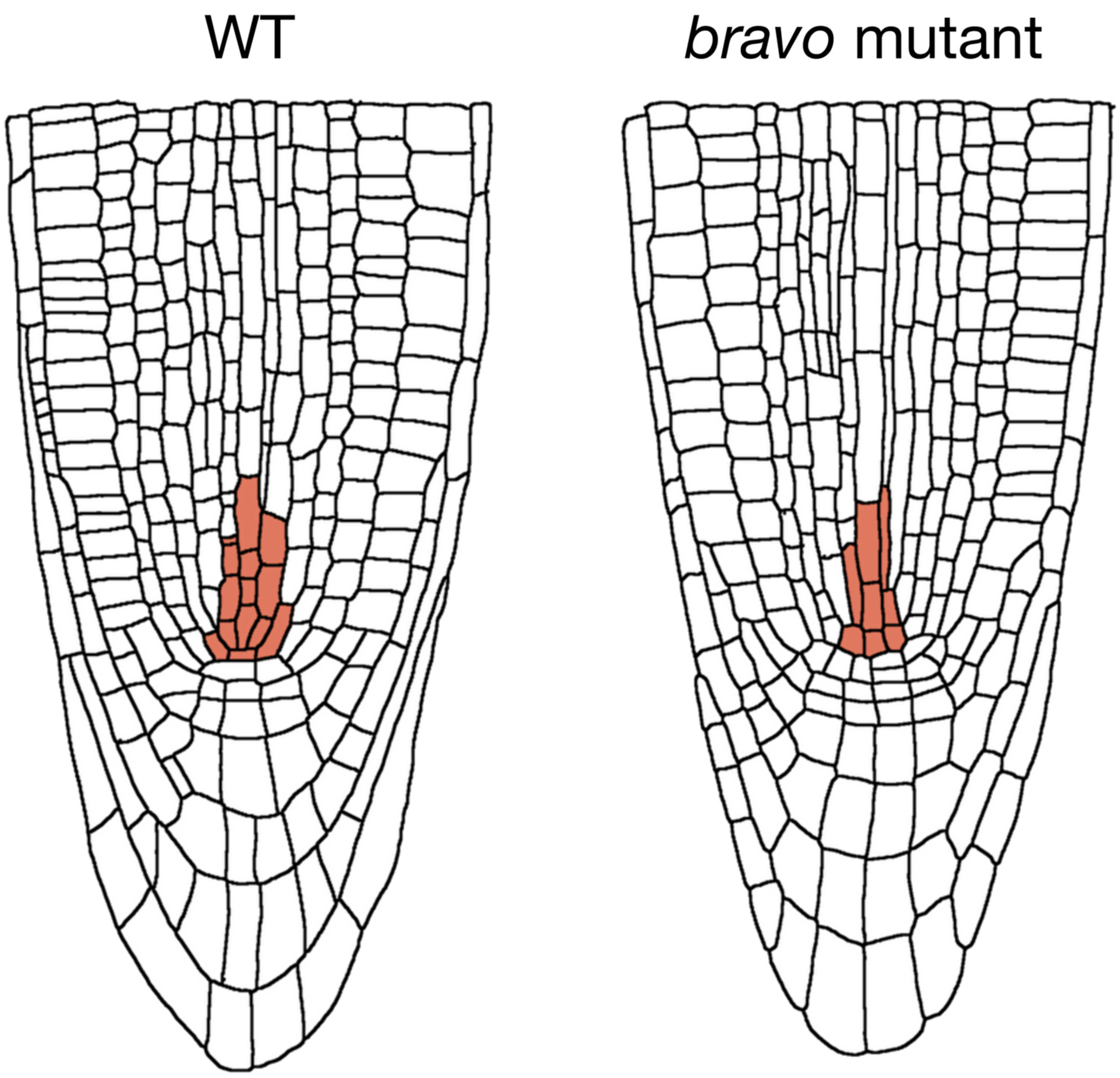

Fig. S10. Domains of basal production of BRAVO.

In the simulations using realistic root layouts, BRAVO is assumed to have basal levels of production only in the first cells of the vasculature coloured in red. This consideration comes from the fact that in double *bravo wox5* mutants, basal expression of *pBRAVO:GFP* can still be observed (Betegón-Putze et al. 2021), with a spatial pattern like the one shown in red. The GFP reporter of BRAVO promoter is also set to have a basal activity only in the red domains. Since WT and *bravo* mutant roots differ in morphology, the specific cells that have basal BRAVO production are slightly different in each root, as depicted.

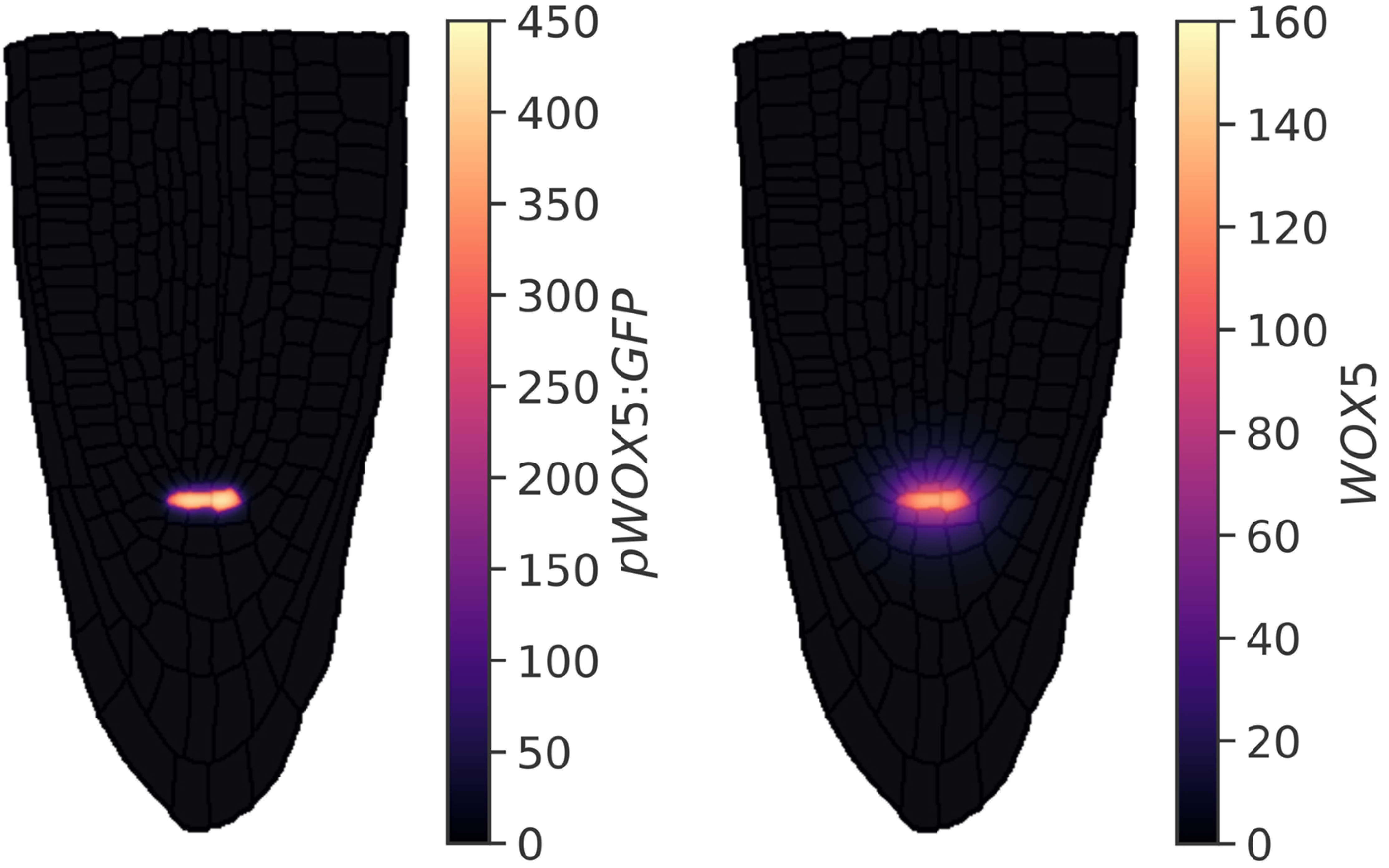

Fig. S11. Concentration of *pWOX5:GFP (W_{GFP})* and *WOX5 (W)* in the absence of other regulatory factors. Stationary patterns in the WT when WOX5 is produced constantly only at the QC, degrades and diffuses, and no other molecule (e.g., BRAVO or Z) is present. The model used is detailed in SI Text. This WOX5 diffusion coefficient allows WOX5 to reach only the cells adjacent to the QC. GFP diffusion and GFP degradation parameter values are also chosen such that *pWOX5:GFP* is mostly localized at the QC. Concentrations shown in this figure are obtained by running the simulations up to a time t = 3000 a.u., at which point the stationary state is already reached. All parameter values of WOX5 and GFP dynamics as in Figure 6 (Table S3).

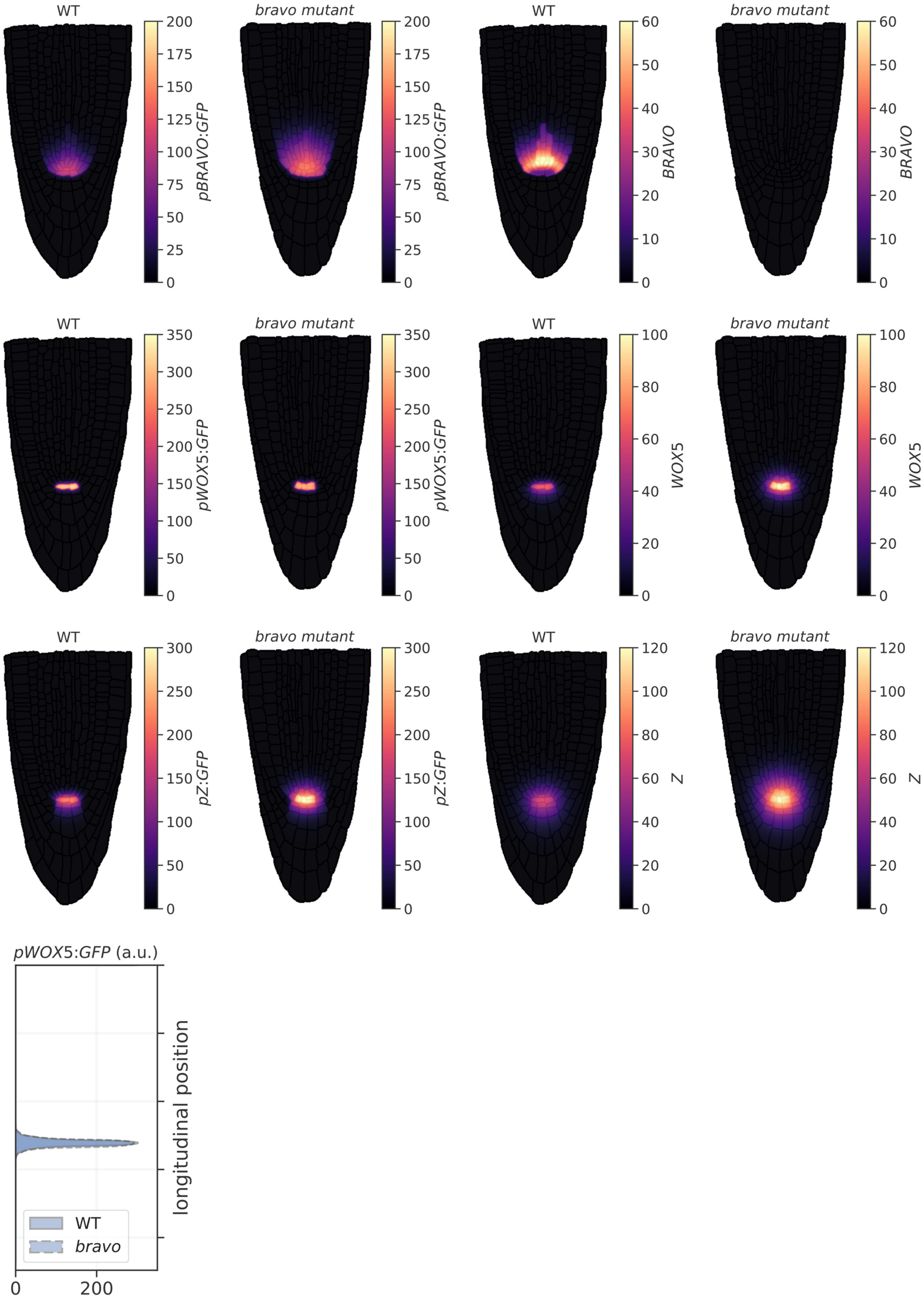

Fig. S12. Stationary profiles of all proteins and GFP reporters of promoters corresponding to the case of Figure 6A (Mixed Model).

The results are shown for the WT and the bravo mutant. The first two panels correspond to the same results shown in Figure 6A and are repeated here for completeness. Parameter values in Table S3. The last panel depicts *pWOX5:GFP* (integrated transversally) along the vertical axis, corresponding to the simulation images above.

Development • Supplementary information

Development: doi:10.1242/dev.200510: Supplementary information

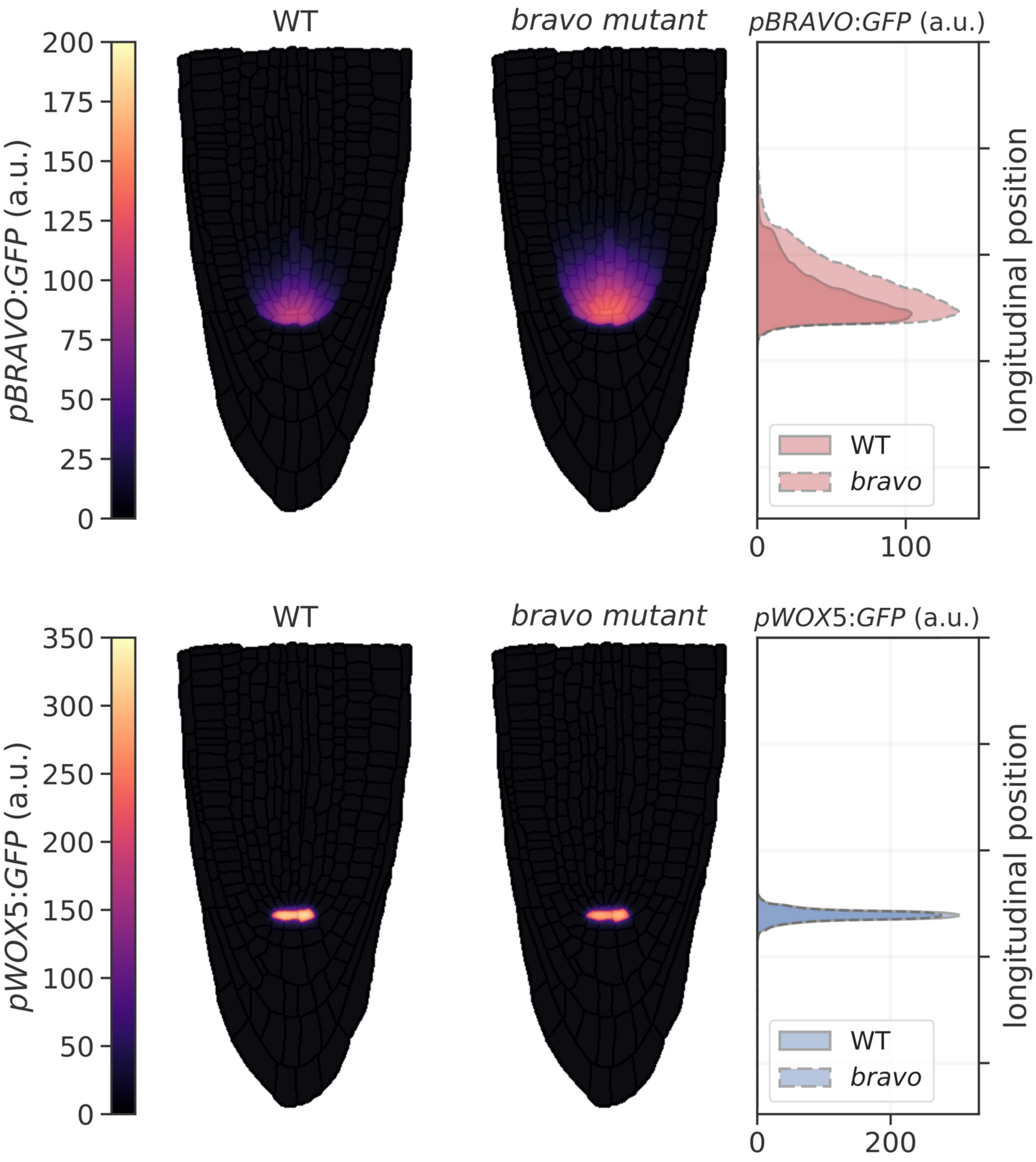

Fig. S13. Effects of root geometry.

GFP reporter activity of the BRAVO and WOX5 promoters for identical root layouts in WT and *bravo* mutants. Results correspond to the same parameters as in Figure 6A and S12 (Table S3). The results are very similar to those obtained using different layouts (Fig. 6A, Fig. S12).

Development • Supplementary information

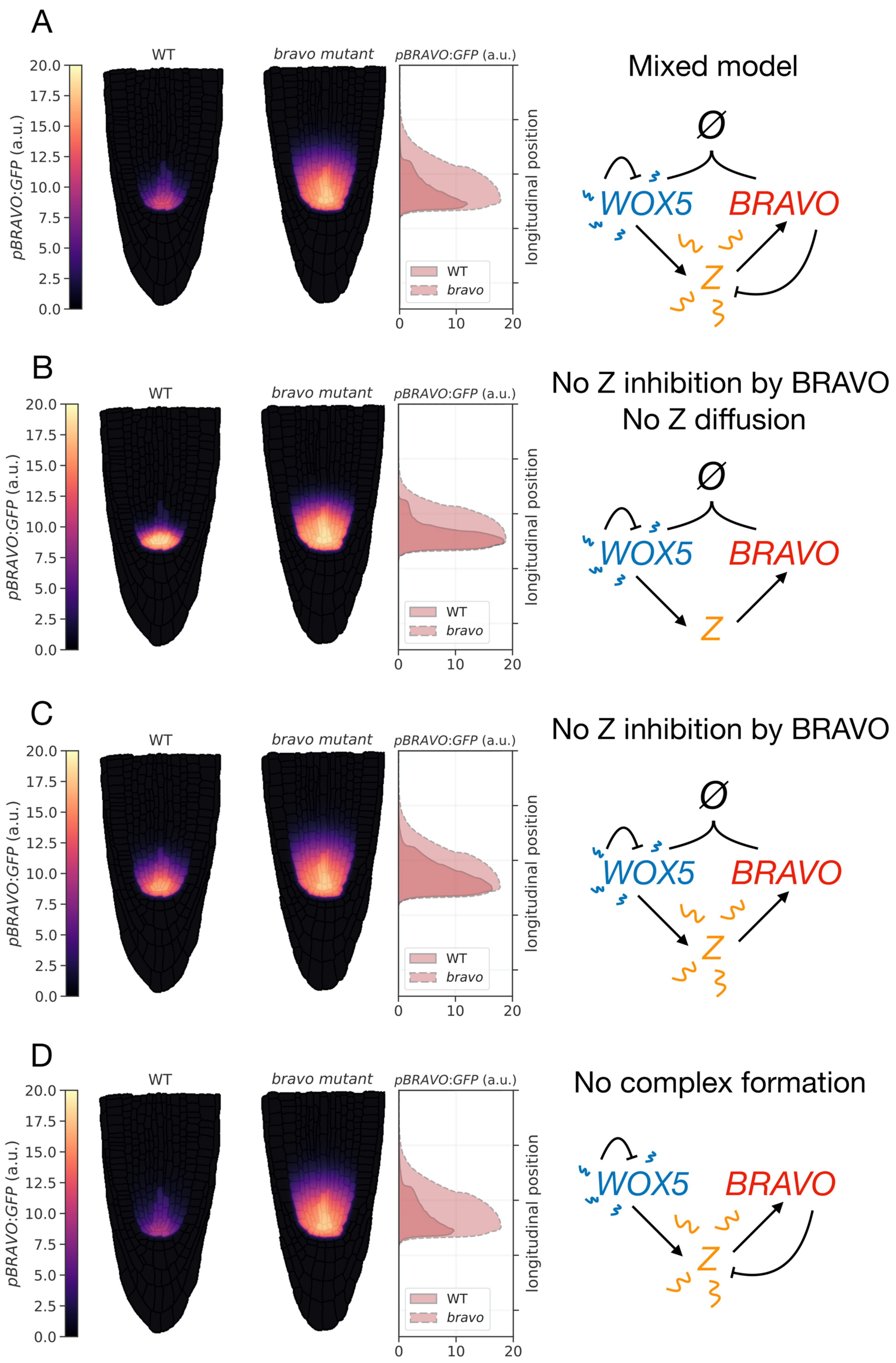

Development • Supplementary information

Fig. S14. ***pBRAVO:GFP*** **simulated in realistic root layouts** for another set of parameter values.

This figure is analogous to Fig. 6 but it is computed for another set of parameter values (that within parenthesis in Table S3). This set drives larger expansions of *pBRAVO:GFP* in the *bravo* mutant. The role of repression is also more relevant here. (A) Mixed model. In the WT, *pBRAVO:GFP* is confined to the SCN, and it is expanded in the *bravo* mutant. (B) Immobilization by sequestration model with non-diffusible Z. This model is sufficient to generate self-confinement in the WT and expansion in the *bravo* mutant. (C) Mixed model without repression (i.e., immobilization by sequestration model with diffusible Z). The absence of repression drives *pBRAVO:GFP* in the WT to reach cells further away from the QC. (D) Absence of complex formation in the mixed model. In this case, the confinement is purely generated by the repression mechanism. For this parameter set, *pBRAVO:GFP* in the WT diminishes due to the stronger repression through Z. Simulations in the *bravo* mutant lead to the same *pBRAVO:GFP* for A, C, and D.

Development • Supplementary information

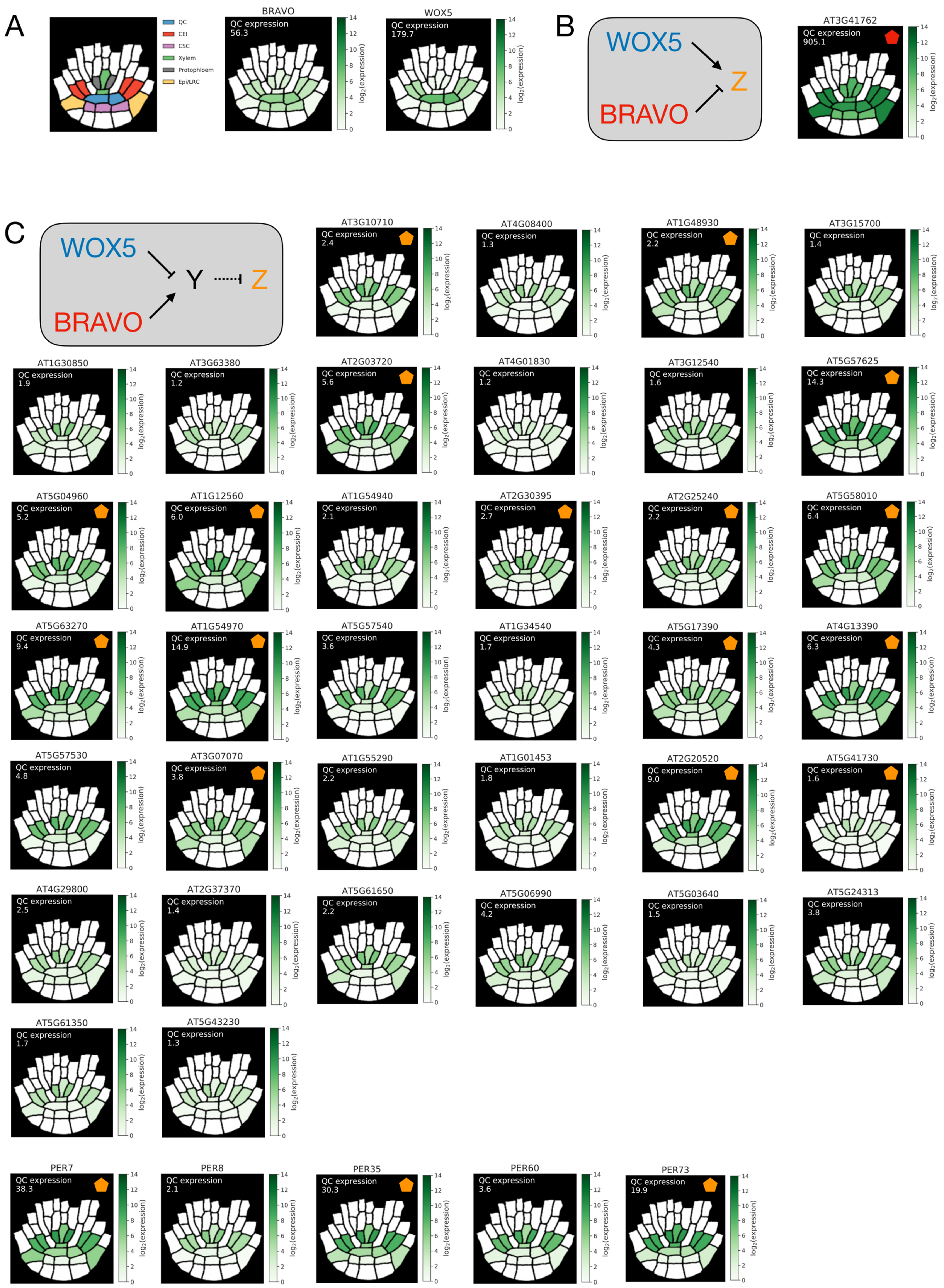
A
QC
CEI
CSC
Xylem
Protophloem
Epi/LRC
BRAVO
QC expression 56.3
WOX5
QC expression 179.7
log2(expression)
B
WOX5
Z
BRAVO
AT3G41762
QC expression 905.1
C
WOX5
Y
Z
BRAVO
AT3G10710
QC expression 2.4
AT4G08400
QC expression 1.3
AT1G48930
QC expression 2.2
AT3G15700
QC expression 1.4
AT1G30850
QC expression 1.9
AT3G63380
QC expression 1.2
AT2G03720
QC expression 5.6
AT4G01830
QC expression 1.2
AT3G12540
QC expression 1.6
AT5G57625
QC expression 14.3
AT5G04960
QC expression 5.2
AT1G12560
QC expression 6.0
AT1G54940
QC expression 2.1
AT2G30395
QC expression 2.7
AT2G25240
QC expression 2.2
AT5G58010
QC expression 6.4
AT5G63270
QC expression 9.4
AT1G54970
QC expression 14.9
AT5G57540
QC expression 3.6
AT1G34540
QC expression 1.7
AT5G17390
QC expression 4.3
AT4G13390
QC expression 6.3
AT5G57530
QC expression 4.8
AT3G07070
QC expression 3.8
AT1G55290
QC expression 2.2
AT1G01453
QC expression 1.8
AT2G20520
QC expression 9.0
AT5G41730
QC expression 1.6
AT4G29800
QC expression 2.5
AT2G37370
QC expression 1.4
AT5G61650
QC expression 2.2
AT5G06990
QC expression 4.2
AT5G03640
QC expression 1.5
AT5G24313
QC expression 3.8
AT5G61350
QC expression 1.7
AT5G43230
QC expression 1.3
PER7
QC expression 38.3
PER8
QC expression 2.1
PER35
QC expression 30.3
PER60
QC expression 3.6
PER73
QC expression 19.9

Fig. S15. Arabidopsis root SCN transcript abundance of genes that are antagonistically regulated by BRAVO and WOX5 in the QC.

(A) Cartoon of the root SCN specifying the cell types. (B-D) WT transcript abundance of different genes in the root SCN of Arabidopsis, specified in each of the cell types indicated in A. The values of transcript abundance in each cell type are taken from RNAseq data from Clark et al. (2019) for WT Arabidopsis and we plotted them in colormap as explained in Methods. Besides their colormap representation, the value of the transcript abundance at the QC is explicitly indicated in each case, below the label "QC expression". (B) BRAVO and WOX5 transcript abundances. (C-D) Transcript abundances for a selection of genes, all of which show antagonistic regulation by BRAVO and WOX5 at the QC. The genes are selected from RNAseq data from Betegón-Putze et al. (2021), from those genes that exhibit significant opposite changes in their transcript abundance at the QC in the *bravo-2* mutant and in the *wox5-1* mutant. (C) Transcript abundance of the only gene that shows significant upregulation in the *bravo-2* mutant and significant downregulation in the *wox5-1* mutant. This gene constitutes a candidate for Z. A diagram of the regulations performed by BRAVO and WOX5 on Z is also plotted: BRAVO represses Z (solid blunt line) and WOX5 induces Z (solid arrow). (D) Transcript abundance of a selection of genes (those in Fig. 6E of Betegón-Putze et al. 2021) that show significant downregulation in the *bravo-2* mutant and upregulation in the *wox5-1* mutant. These genes are candidates for molecule Y, being repressed by WOX5 (blunt line) and induced by BRAVO (arrow). If any of these genes non-transcriptionally inhibits a certain factor, this could be a candidate for Z. In B and C, the coloured pentagons on the top right corners mark the genes that we suggest as more plausible candidates based on the following criteria: In C, if the gene exhibits an abundance higher than BRAVO at the QC in the WT, based on Clark et al. 2019. In D, if the gene in the *wox5-1* mutant exhibits an abundance at the QC higher than BRAVO at the QC of the WT. Hence, the criterium is based on setting BRAVO WT expression at the QC as the minimal abundance and comparing it with the abundance of the genes when they are not repressed.

Development • Supplementary information

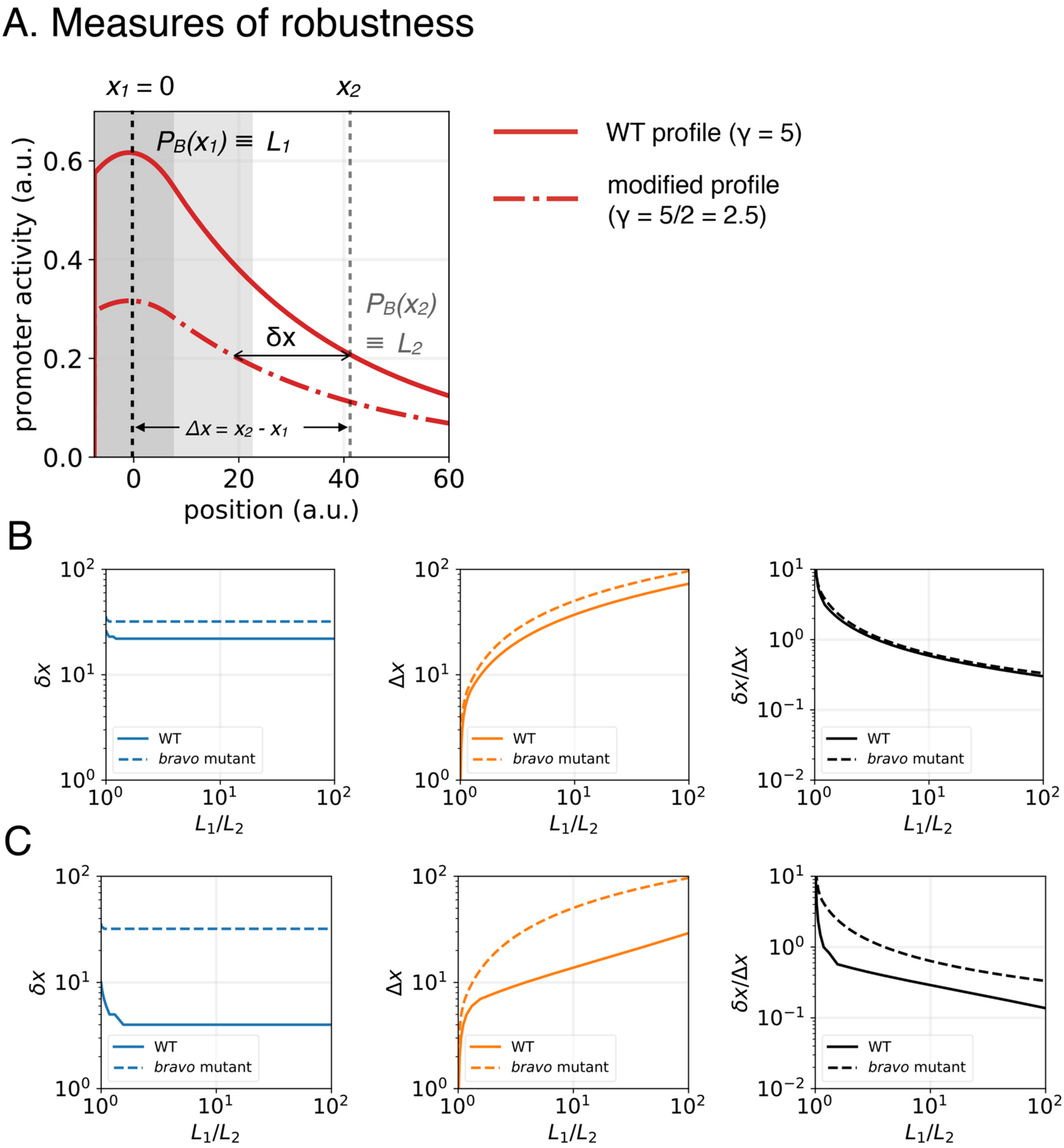

A. Measures of robustness
$x_1 = 0$
x_2
$P_B(x_1) \equiv L_1$
$P_B(x_2) \equiv L_2$
δx
$\Delta x = x_2 - x_1$
promoter activity (a.u.)
position (a.u.)
WT profile (γ = 5)
modified profile (γ = 5/2 = 2.5)
B
C
δx
Δx
δx/Δx
L_1/L_2
WT
bravo mutant

Fig. S16. Robustness of pBRAVO expression upon changes in WOX5 expression in the QC. (A)Sketch showing the measures we use to quantify robustness following Eldar et al. (2003). Two values of the *pB* gradient, L_1 and L_2, are selected. Each of these values corresponds to a spatial position, x_1 and x_2, and define $\Delta x = x_2 - x_1$. The *pB* profile when WOX5 production is reduced to half its original value is computed and the spatial position where it takes the value L_2 is termed x_2'. δx evaluates the spatial change of this position compared to x_2, $\delta x = x_2' - x_2$. L_1 value is set fixed while different values of L_2 are analysed. As L_2 changes, both the quantities δx and Δx change, and robustness is quantified by the ratio $\frac{\delta x}{\Delta x}$ as a function of the ratio $\frac{L_1}{L_2}$. In this figure we show the results of this measure for the immobilization by sequestration model in the WT and in the *bravo* mutant. For this model, in the *bravo* mutant there is no mechanism that can drive robustness (i.e., it has an exponential profile), whereas in the WT we wonder whether sequestration can drive robustness to perturbations on the level of WOX5. The parameters used for the profiles are those of Table S1 for the linear model, with $\gamma = 5$ (continuous line) and $\gamma = 2.5$ (dot-dashed line). (B) In the linear immobilization by sequestration model, WT and *bravo* mutant profiles have very similar values of $\frac{\delta x}{\Delta x}$, meaning that there is no robustness in the spatial profiles of the BRAVO promoter with respect to the *bravo* mutant, at least with the default parameter set in Table S1 (α=0.01). Hence, BRAVO and its sequestration does not seem to confer higher robustness to changes in WOX5 expression. (C) For higher values of α (α=0.5), however, the curve corresponding to the WT profiles show lower values than in the *bravo* mutant, meaning that in the WT the BRAVO expression profiles are more robust to perturbations of WOX5 expression in the QC. This situation corresponds to values of the BRAVO promoter that are too high when compared with those of the WOX5 promoter.

Table S1. Default parameters of the immobilization by sequestration (IS), repression (R) and mixed (M) models for 1D simulations. All parameter values are set in arbitrary units.

Parameter	Description	IS	R	M
α_0	Basal BRAVO production rate	-	0	0.2
α_L (linear)<br>α (non-linear)	Protein production rate of BRAVO	0.01 (linear)<br>2 (non-linear)	0.01 (linear)<br>2 (non-linear)	-<br>2
γ	Protein production rate of WOX5	5	5	5
β_L(linear)<br>β (non-linear)	Protein production rate of Z	-<br>10 (intermediary)	0.01 (linear)<br>10 (non-linear)	-<br>10
c	Threshold of Z repression by BRAVO	-	0.01	0.01
λ	Binding rate between BRAVO and WOX5	0.001	0.001	0.001
k_B	Threshold of BRAVO activation by W or Z	50	50	50
k_W	Threshold of WOX5 self-repression	200	200	200
k_Z	Threshold of Z activation by WOX5	200	200	200
n (only nonlinear)	Cooperativity	1<br>3 (Fig. S2B)	1<br>3 (Fig. S6B)	1<br>3 (Fig. S7B)
d_B	Degradation rate of BRAVO proteins	0.01	0.01	0.01
d_W	Degradation rate of WOX5 proteins	0.01	0.01	0.01
d_Z	Degradation rate of Z proteins	0.01<br>(intermediary)	0.01	0.01
D_W	Diffusion coefficient of WOX5 proteins	4<br>2 (intermediary)	0	2
D_Z	Diffusion coefficient of Z proteins	-<br>2 (intermediary)	4	2

Development • Supplementary information

Table S2. Additional parameters for the immobilization by sequestration model with an additional sequestrator (ISAS). The remaining parameters are the same as in the original immobilization by sequestration model. All parameter values are set in arbitrary units.

Parameter	Description	ISAS
α_s	Protein production rate of *S*	0.1
d_s	Degradation rate of *S* proteins	0.01
λ_{BS}	Complex formation rate between BRAVO and *S*	0.001
λ_{WS}	Complex formation rate between WOX5 and *S*	0.001

Table S3. Default parameters of the mixed model in the realistic root layout. All parameter values are set in arbitrary units of concentration, time and space.

Parameter	Description	Value (a.u.)
α_0	Basal production rate of BRAVO proteins	0.02
α	Regulated production rate of BRAVO proteins	2
γ_{QC}	Production rate of WOX5 proteins	5
β	Production rate of *Z* proteins	10
k_B	Threshold of BRAVO activation by *Z*	50
k_Z	Threshold of *Z* activation by WOX5	200
k_W	Threshold of WOX5 self-repression	200
c	Threshold of *Z* repression by BRAVO	0.01
λ	Binding rate between BRAVO and WOX5	0.001
d_B	Degradation rate of BRAVO proteins	0.01
d_W	Degradation rate of WOX5 proteins	0.01
n	Cooperativity	1
d_Z	Degradation rate of *Z* proteins	0.01
D_W^{cyt}	Diffusion coefficient of WOX5 proteins in the cytoplasm	2
D_W^{wall}	Diffusion coefficient of WOX5 proteins in cell walls	0.5
D_Z^{cyt}	Diffusion coefficient of *Z* proteins in the cytoplasm	4
D_Z^{wall}	Diffusion coefficient of *Z* proteins in cell walls	1
D_{GFP}^{cyt}	Diffusion coefficient of GFP proteins in the cytoplasm	0.1
D_{GFP}^{wall}	Diffusion coefficient of GFP proteins in cell walls	0.1
d_{GFP}	Degradation rate of GFP proteins	0.01

Development • Supplementary information

Supplementary Materials and Methods

1 Derivation of the models

The equations used in all models come from an approximation where complex formation and mRNA dynamics is very fast compared to the dynamics of proteins. We exemplify this approximation with the immobilization by sequestration mechanism with linear production, but the same procedure has been applied to obtain the set of equations of all the other models.

By explicitly considering the mRNA of BRAVO (m_B) and WOX5 (m_W), and the complex formed by the binding of BRAVO and WOX5 proteins (C), the model equations for all these variables and for BRAVO (B) and WOX5 (W) proteins in the immobilization by sequestration mechanism are:

$$\frac{\partial m_B(x,t)}{\partial t} = a_B W(x,t) - \delta_B m_B(x,t) \quad (1)$$

$$\frac{\partial m_W(x,t)}{\partial t} = a_W^{QC}(x) - \delta_W m_W(x,t) \quad (2)$$

$$\frac{\partial B(x,t)}{\partial t} = \alpha_B m_B(x,t) - \mu B(x,t)W(x,t) + \nu C(x,t) - d_B B(x,t) \quad (3)$$

$$\frac{\partial W(x,t)}{\partial t} = \gamma_W m_W(x,t) - \mu B(x,t)W(x,t) + \nu C(x,t) - d_W W(x,t) + D_W \frac{\partial^2 W(x,t)}{\partial x^2} \quad (4)$$

$$\frac{\partial C(x,t)}{\partial t} = \mu B(x,t)W(x,t) - \nu C(x,t) - d_C C(x,t) \quad (5)$$

where a_B, $a_W^{QC}(x)$, δ_B, δ_W are the mRNA synthesis and degradation rates of BRAVO and WOX5 mRNAs, respectively. The superscript and explicit spatial dependence in $a_W^{QC}(x)$ indicates that the mRNA of WOX5 is only produced in the QC region. α_B and γ_W represent the rates of mRNA translation into BRAVO and WOX5 proteins, respectively, and μ and ν are the rates of protein-protein binding and unbinding. Finally, the complex can be degraded with rate d_C. The rest of the parameters have already been defined in the Methods section. If the dynamics of the mRNAs and of complex formation are very fast (by setting their corresponding time derivatives to zero), we obtain:

$$m_B(x) = \frac{a_B W(x)}{\delta_B}, \; m_W(x) = \frac{a_W^{QC}(x)}{\delta_W}, \; C(x) = \frac{\mu B(x)W(x)}{\nu + d_C} \quad (6)$$

Development • Supplementary information

Substituting these relations to the equations for B and W, it results into:

$$\frac{\partial B(x,t)}{\partial t} = \frac{\alpha_B a_B}{\delta_B} W(x,t) - \left(\mu + \frac{\nu\mu}{\nu + d_C}\right) B(x,t)W(x,t) - d_B B(x,t) \quad (7)$$

$$\frac{\partial W(x,t)}{\partial t} = \frac{\gamma_W a_W^{QC}(x)}{\delta_W} - \left(\mu + \frac{\nu\mu}{\nu + d_C}\right) B(x,t)W(x,t) - d_W W(x,t) + D_W \frac{\partial^2 W(x,t)}{\partial x^2} \quad (8)$$

These are the equations of the immobilization by sequestration model used in the main text (Eq. 1 and 2) when the following definitions of parameters are applied: $\alpha_L \equiv \frac{\alpha_B a_B}{\delta_B}$, $\gamma_{QC}(x) \equiv \frac{\gamma_W a_W^{QC}(x)}{\delta_W}$ and $\lambda \equiv \mu + \frac{\nu\mu}{\nu + d_C}$.
Notice that since we only analyse the stationary state of the system, these quasi-steady state approximations do not affect the final result of the spatial profiles.

1.1 Stationary profile of WOX5 in the immobilization by sequestration model

In the stationary state, the previous equations (7) and (8) of the immobilization by sequestration model can be reduced to the following second order ODE:

$$D_W \frac{d^2 W(x)}{dx^2} = -\gamma_{QC}(x) + W(x) \left(\frac{W(x)\lambda\alpha_L}{\lambda W(x) + d_B} + d_W \right) \quad (9)$$

and the BRAVO profile is $B(x) = \frac{\lambda\alpha_L}{\lambda W(x) + d_B}$. This ODE can be interpreted as a diffusing molecule W produced at a source $\gamma_{QC}(x)$, with a *nonlinear* higher degradation rate, given by

$$d'_W[W(x)] \equiv d_W + \frac{W(x)\lambda\alpha_L}{\lambda W(x) + d_B} \quad (10)$$

where the second term comes from the binding between BRAVO and WOX5. This non-linear degradation implies that in the immobilization by sequestration model and outside the source region, the stationary spatial profile of $W(x)$ in the WT is not exponential, but decays spatially more abruptly. In contrast, when modeling the *bravo* mutant, the same ODE applies but with a linear degradation, $d'_W[W(x)] = d_W$ and hence the profile of $W(x)$ outside the source region is exponential in this mutant.

Development • Supplementary information

2 Immobilization by sequestration with an additional sequestrator

In Figure S3 we show the effect of an additional protein (hereafter named sequestrator, S) which can bind BRAVO and WOX5 separately. The equations corresponding to this model are:

$$\frac{\partial B(x,t)}{\partial t} = \alpha_L W(x,t) - \lambda B(x,t)W(x,t) - \lambda_{BS} B(x,t)S(x,t) - d_B B(x,t) \tag{11}$$

$$\frac{\partial W(x,t)}{\partial t} = \gamma_{QC}(x) - \lambda B(x,t)W(x,t) - \lambda_{WS} W(x,t)S(x,t) - d_W W(x,t) + D_W \frac{\partial^2 W(x,t)}{\partial x^2} \tag{12}$$

$$\frac{\partial S(x,t)}{\partial t} = \alpha_S - \lambda_{BS} B(x,t)S(x,t) - \lambda_{WS} W(x,t)S(x,t) - d_S S(x,t) \tag{13}$$

where $S(x,t)$ is the concentration of the protein S across space and time, the new parameters α_S and d_S represent the production and degradation of S proteins, and λ_{BS}, λ_{WS} denote the complex formation rates between S and BRAVO and S and WOX5, respectively.

3 Simulations in a realistic root layout

3.1 Space discretization for diffusion with heterogeneous coefficients

In the realistic root layout, we simulate a reaction-diffusion equation with non-homogeneous diffusion coefficients, that is, diffusion is explicitly dependent on space. In our case, the value of these coefficients depend on whether the spatial position corresponds to the interior of a cell or to the cell wall, with respective diffusion coefficients of D^{cyt} and D^{wall}. These can be encompassed into a single, spatially-dependent coefficient $D(\vec{r})$, where the vector $\vec{r} \equiv (x,y)$ carries the information of the positions within the root layout:

$D(\vec{r}) = D^{cyt}$ for $\vec{r} \in$ cells and $D(\vec{r}) = D^{wall}$ for $\vec{r} \in$ cell wall.

Then, for a given variable $u(\vec{r})$ (i.e. the concentration of one of the proteins), we discretize the spatial term $\vec{\nabla}(D(\vec{r})\vec{\nabla}u(\vec{r}))$ as done in [1], namely:

$$\vec{\nabla}(D_{ij}\vec{\nabla}u_{ij}) = \frac{1}{\Delta x^2}\left(\frac{1}{2}(D_{ij}+D_{i+1,j})(u_{i+1,j}-u_{ij}) - \frac{1}{2}(D_{ij}+D_{i-1,j})(u_{ij}-u_{i-1,j})\right) +$$

Development • Supplementary information

$$+\frac{1}{\Delta y^2}\left(\frac{1}{2}(D_{ij}+D_{i,j+1})(u_{i,j+1}-u_{ij})-\frac{1}{2}(D_{ij}+D_{i,j-1})(u_{ij}-u_{i,j-1})\right)$$

where $D_{ij} = D(x = i\Delta x, y = j\Delta y)$ and $u_{ij} = u(x = i\Delta x, y = j\Delta y)$. Thus the values of indexes i, j specify the value of the diffusion coefficient, whether it is D^{cyt} or D^{wall}.

3.2 Reaction-diffusion model with only WOX5

Figure S11 shows the stationary results when in the QC only WOX5 is produced, becomes degraded and diffuses, in the absence of any other regulatory factor. In this case only the dynamics for WOX5 and the GFP reporter its promoter are simulated, according to the following equations inside the cells:

$$\frac{\partial W(\vec{r},t)}{\partial t} = \gamma_{QC}(\vec{r}) - d_W W(\vec{r},t) + \vec{\nabla}\left[D_W^{cyt}(\vec{r})\vec{\nabla}W(\vec{r},t)\right] \tag{14}$$

$$\frac{\partial W_{GFP}(\vec{r},t)}{\partial t} = \gamma_{QC}(\vec{r}) - d_{GFP}W_{GFP}(\vec{r},t) + \vec{\nabla}\left[D_{GFP}^{cyt}(\vec{r})\vec{\nabla}W_{GFP}(\vec{r},t)\right] \tag{15}$$

and in the cell wall:

$$\frac{\partial W(\vec{r},t)}{\partial t} = \vec{\nabla}\left[D_W^{wall}(\vec{r})\vec{\nabla}W(\vec{r},t)\right] \tag{16}$$

$$\frac{\partial W_{GFP}(\vec{r},t)}{\partial t} = \vec{\nabla}\left[D_{GFP}^{wall}(\vec{r})\vec{\nabla}W_{GFP}(\vec{r},t)\right] \tag{17}$$

Notice that for these variables, these are the same equations as those of the Mixed model but with $\lambda = 0$.

References

[1] Jordan Lee. **Stability of Finite Difference Schemes on the Diffusion Equation with Discontinuous Coefficients**. https://math.mit.edu/research/highschool/rsi/documents/2017Lee.pdf, 2017.

Development • Supplementary information
