# Supplementary material for: BRAVO self-confined expression through WOX5 in the Arabidopsis root stem-cell niche
Source: Development. 2022 Jul 28;149(15):dev200510. doi: 10.1242/dev.200510 (PMC9440757; doi:10.1242/dev.200510)
Supplement: Reviewer comments [file develop-149-200510_review_history.pdf]

BRAVO self-confined expression through WOX5 in the *Arabidopsis* root stem-cell niche

Josep Mercadal, Isabel Betegón-Putze, Nadja Bosch, Ana I. Caño-Delgado and Marta Ibañes
DOI: 10.1242/dev.200510

Editor: Ykä Helariutta

Review timeline

Original submission:	8 January 2022
Editorial decision:	21 February 2022
First revision received:	26 May 2022
Accepted:	23 June 2022

Original submission

First decision letter

MS ID#: DEVELOP/2022/200510

MS TITLE: *BRAVO* self-confined expression through WOX5 in the *Arabidopsis* root stem-cell niche

AUTHORS: Josep Mercadal, Isabel Betegón-Putze, Nadja Bosch, Ana I. Caño-Delgado and Marta Ibañes

I have now received all the referees' reports on the above manuscript, and have reached a decision. The referees' comments are appended below, or you can access them online: please go to BenchPress and click on the 'Manuscripts with Decisions' queue in the Author Area.

As you will see, the referees express considerable interest in your work, but have some significant criticisms and recommend a substantial revision of your manuscript before we can consider publication. If you are able to revise the manuscript along the lines suggested, which may involve further experiments, I will be happy receive a revised version of the manuscript. Your revised paper will be re-reviewed by one or more of the original referees, and acceptance of your manuscript will depend on your addressing satisfactorily the reviewers' major concerns. Please also note that Development will normally permit only one round of major revision.

We are aware that you may be experiencing disruption to the normal running of your lab that make experimental revisions challenging. If it would be helpful, we encourage you to contact us to discuss your revision in greater detail. Please send us a point-by-point response indicating where you are able to address concerns raised (either experimentally or by changes to the text) and where you will not be able to do so within the normal timeframe of a revision. We will then provide further guidance. Please also note that we are happy to extend revision timeframes as necessary.

Please attend to all of the reviewers' comments and ensure that you clearly highlight all changes made in the revised manuscript. Please avoid using 'Tracked changes' in Word files as these are lost in PDF conversion. I should be grateful if you would also provide a point-by-point response detailing how you have dealt with the points raised by the reviewers in the 'Response to Reviewers' box. If you do not agree with any of their criticisms or suggestions please explain clearly why this is so.

© 2022. Published by The Company of Biologists under the terms of the Creative Commons Attribution License (https://creativecommons.org/licenses/by/4.0/).

Reviewer 1

Advance summary and potential significance to field

This paper explores different networks that may confine the expression of two transcription factors, BRAVO and WOX5 within the stem cell niche. It uses a set of experimental observations mostly published in previous papers by the authors, and explores the effect that altering network topology would have on the ability of a WOX5-BRAVO module to restrict the expression of BRAVO.

This work firstly involves the exploration of three models within a one dimensional framework. Although any of these scenarios could regulate patterning there are restrictions that make them more or less likely. The patterning ability is then explored in a rather beautiful 2D model based on realistic geometries.
This model further clarifies the feasibility of immobilisation of BRAVO by WOX5 to self-confine BRAVO. The model does not (and cannot) predict which of these scenarios operates, but provides important insights into what assumptions/paramaters would be necessary to enable these. For example differences in the relative expression levels of WOX5 and BRAVO support some versions more strongly.

This is an important contribution, and forms an important piece in the puzzle of regulation of the root stem cell niche. One particular area of interest is the proposal of an as yet unidentified protein downstream of WOX5 that regulates BRAVO. The paper provides insight into how such a protein would behave (mobile, and regulated in an opposing manner in bravo and wox5) that identifying such a protein would not be a distant proposition. It also serves as a neat exemplar of the effect that relatively small changes to network structure or diffusion of molecules can have on patterning. As such, I think it would have relevance across different stem cell niches and would be suitable for publication in broad journal such as Development.

Comments for the author

I am enthusiastic about the paper, but have some minor comments.
Title I think this simply has to have BRAVO and WOX in the title. In my opinion the current title is simply too broad.
Abstract I feel the description of the Root Stem Cell niche is too centred on WOX5 and BRAVO. The introduction is more balanced, but I think abstract doesn't put the work in context in the wider context of stem cell regulation.
Results As all models do, this relies on assumptions based on experimentation. In this case the relevant experiments had all been published and were cited, but I found myself having to go back to the literature to understand the rationale behind them. I found the images in 1B-E unconvincing. The data in the original paper (Betegon-Putze) was very convincing with quantification of the differences in expression pattern. I would have appreciated a better description of the data supporting these assumptions. In particular, I had problems understanding what the evidence for WOX5 being expressed at higher levels than BRAVO. Was this based on the reporters, or on RNASeq? I feel this is important as it is used as a method later to separate between the models.
The 1D models were clear but the schematics e.g. 2A show network topology but lack any spatial framework. This might be better served with an additional schematic in showing the spatial movement of components (WOX5).
I felt that the attenuation by sequestration model was peculiar. The authors state that in this model WOX5 does not move, but they note that in the experimental data it moves slowly. Given the discrepancy between assumption and observation, I would have expected to have seen more exploration of the parameter space around WOX5 mobility. Is it essential for the model that WOX5 doesn't move? If so, could the authors explain their rationale behind this model more clearly. The same comment about WOX5 movement is also relevant for the repression model.
The 2D modelling in realistic geometries was very elegant, but this part seemed to finish quite abruptly. I understand that the models are useful to see how the patterns of these two reporters shift, but I wonder if it is possible to more closely compare the predicted patterns with experimental data. I'm assuming that the middle panels on Fig 5 are model predictions. Is it possible to present side-by-side experimental data in this way too?

© 2022. Published by The Company of Biologists under the terms of the Creative Commons Attribution License (https://creativecommons.org/licenses/by/4.0/).

Reviewer 2

Advance summary and potential significance to field

The paper uses computer simulations of mathematical models to address the question of how regulatory interactions between transcription factors can underlie their spatial restriction in a stem cell niche. The focus is on the stem cell niche in the primary root of the plant Arabisopsis thaliana, and the transcription factors WOX5 and BRAVO. This is a well-studied system, but the models discussed here build on existing data (largely from this group) to address an important question that is currently unresolved.

The paper uses a suite of related models to explore the potential contributions of different types of interactions to localisation. Interestingly, models are simulated in highly idealised one-dimensional form and in more realistic data-
derived 2D form. The former allows a clear focus on the roles of the different regulatory mechanisms while the latter then allows the consequences of these mechanisms to be studied in the context of the cellular architcture of the root.

The research reported in the paper represents two advances:

1. The modelling shows the sufficiency of a mechanism based on "attenuation of a mobile activator" for localisation. The mechanism fits into a broader theme of spatially-restricted intercellular signalling in plants based on mobile factors (typically transcription factors and micro RNAs) and restricting interactions but introduces a novel variant (distinct to the proposed mechanism in the shoot apical meristem, for example). This provides an additional principle that can underlie restriction of activities to stem cell niches (which could also apply in animal tissues if taken as an abstract principle).

2. It provides a nice worked example of the simulation of intercellular movement and regulatory interactions in data-derived root tissue geometries.

Both these advances speak specifically to the question of the regulation of the stem cell niche in the Arabisopsis root, and to the broader question of how to understand and model the regulation of stem cell niches generally.

The paper demonstrates the sufficiency of the proposed mechanisms to account for current data on wild type and mutant expression patterns, and maps out (roughly)
the spaces of appropriate parameters (i.e. relative weights of different regulatory interactions). There are no new data presented to support the modelling.

Mathematical and numerical details are self-contained in the Methods section and Supplementary Information, and the paper should be accessible to a broad audience.

Comments for the author

1. I like the combination of rather idealised models followed by simulations in the more realistic root geometries. I think the rationale for the two could be spelled out more clearly, and that this would be helpful for the structure of the paper. It would help if the roles being played by the two modelling approaches were explained (as a reader, I might ask why you don't move immediately to the final models). In tandem with this, I think the principal assumptions that go in to each model could be made clearer. As it stands, the reader has to do some work to pull out the assumptions from the text.
2. Given that (as I see it) the initial models are there to allow a clear exploration of the relative contributions of the different types of regulatory interaction (complex formation, feedback regulation, etc.), I think it would be valuable to be more explicit in mapping out the parameter domains that give results compatible with existing data. Also, the constraints imposed by these data need to made more explicit (I infer from the paper that we should expect WOX5 promoter activity [in some sense] to be around 5 times higher than BRAVO promoter activity, but this isn't made very clear; I'm less clear about the numerical data on the extent of expression domain

© 2022. Published by The Company of Biologists under the terms of the Creative Commons Attribution License (https://creativecommons.org/licenses/by/4.0/).

changes in the mutants). Can these be made clear and then the appropriate parameter domains be mapped out (e.g. in a lambda-alpha plane for the "immobilisation by sequestration" model)?
3. Are there any data available on WOX5 protein distributions (not pWOX5::GFP)?
The models make predictions about the spatial distribution of WOX5 protein (when it is assumed to move), and it would be really helpful to be able to compare to these.
4. Some of the models depend quite sensitively on the balance between the concentrations of WOX5 protein, BRAVO protein, and protein complexes. I appreciate that complex concentrations have been ommited for clarity, but I find it difficult to really get a deep feel for the operation of some of the models without having these results. They could be in the Supplement, but I think they're an important part of the models (as alluded to in the paper at various points) and should be presented.
5. The idealised models use linear production terms, while the simulations on root geometries use nonlinear terms. I appreciate the value of the extra simplicity (reduced parameters) of the idealised models, but it would be interesting to know how the use of similar nonlinear production terms would affect the conclusions about appropriate parameter domains (since the balance between production [nonlinear; saturating] and binding [mass action] would be different at different concentrations).
6. The discussion of the effect of a general squestrator S is rather cursory
(lines 159-163). I'd like to know more, especially since it is known that there are other binding factors in the stem cell niche. What are the constraints? How much complex of B and W with S can be permitted compared to B with W? It is clear from the results that some S is permissible, but I can't get a good feel for how much. Since it is assumed that [BRAVO] << [WOX5], I presume things hinge on the relative concentrations of BRAVO and S. Is this right, and can it be determined from the simulations?
7. There are problems with Eqs. (34) and (35), and the expression for B on line 768. The expression for B should be
B = \frac{\alpha W}{\lambda W + d_B}
and then
d_W' = d_W + \frac{\lambda \alpha W}{\lambda W + d_B}.

B is an increasing function of W, and the effective degradation rate of W is an _increasing_ function of W. These need to be corrected, but I'm confident that these are typos and that the simulations use the correct forms.
8. Since the "attenuation by sequestration" model doesn't fit with data, is it really worth including it in the main text? I'd be inclined to restrict it to the Supplement, at best. As it is, it detracts from the logical flow of the main text.
9. It is stated that the results of transcriptomics in bravo and wox5 mutants yield potential candidates for Z. Can you say anything more than that? It would be really valuable to know some of the potential candidates. This would give a way of selecting between alternatives / validating the models. Are there any candidates with known expression patterns that match that expected of Z from the simulations?
10. On line 293, you state that pWOX5 activity should decrease in the bravo mutant. Is this observable? Are there any data to compare to?
11. The full root models have basal BRAVO production in the vasculature (in line with data) and WOX5 auto-repression (inspired by data). I think these should really be in the idealised models, too (since they're directly reflecting the data).
12. On line 295, it is claimed that the parameters are "biologically reasonable". Can you expand on this a bit to justify the claim? Is GFP really likely to be less diffisible than the TFs? How easily can you interpret movement parameters when the interior of the cell is taken as homogeneous (no vacuole)?
13. On line 298, you discuss conditions for the restriction of pWOX5 activity mostly to the QC, VI and CSC. However, the model claims to impose pWOX5 activity _only_ in the QC (by \gamma_{QC}(r)). I'm confused - you can't have expression outside the QC in the model...
14. One feature that has emerged from studies of morphogen gradients in animal tissues is the role that complex formation and feedbacks can make to buffering morphogen range against variation in source strength (e.g. citations [35] and
[36] for BMP, and Irons et al. (2010). Dev. Bio. 342, 180–193 for Hedgehog).
Given the broad parallels with the models described here, it would be really interesting to know if the proposed mechanisms for restricting the expression of SCN factors can also contribute to buffering against source strength. It would be well worth seeing the effect of changes in the WOX5

© 2022. Published by The Company of Biologists under the terms of the Creative Commons Attribution License (https://creativecommons.org/licenses/by/4.0/).

production rate (\gamma_{QC}), and how the interactions with BRAVO might buffer this. This has been a significant area of study for animal morphogen gradients, and the question is equally pertinent for plant tissues. Specifically, the QC is specified at least in part by hormone profiles, and some quantitative variability in these is to be expected (even if the position of, say, an auxin maximum is relatively stable). A robust mechanism for maintaining a stable SCN will surely require some buffering against quantitative fluctuations.

Minor comments:
- the diffusion rates in the figure legends are wrong at some points: D_W in Fig. 2, D_X in Fig. 3 and D_Z in Fig. 4. I believe they should be 0.01, not 4.
- the formatting of the \lambdas in the legend to Fig. 7 has gone awry.
- on line 202, you say "reducing the production of its mobile activator". I think it is actually reducing the diffusion of the mobile activator, not its production.
- you use different cellular architectures for the wild type and bravo mutant roots. Are there data to show that the mutant has a clearly and reproducibly distinct geometry to the wild type? If so, can you summarise the key differences? Does it make much difference to the outcome of the study?
- since all the figures / conclusions depend only on steady state distributions the discussion of fast complex dynamics is a bit distracting. It is only being used to motivate the reduction of the models to lower numbers of variables, but then the reduced equations are not really being used to infer much. Is it worth reducing the prominence of this, or re-wording somehow?
- the simulations of the models require the specification of boundary conditions, but I can't find any mention of these. Please include them.
- the title could be changed to be a bit more informative to the (potential) reader.

<u>Reviewer 3</u>

Advance summary and potential significance to field

The articles subject is of a relatively limited scope, it only adresses the narrowing of the BRAVO and WOX5 protein domeins in the Arabidopsis root stem cell niche, which furthermore was previously extensively adressed in another article of the same research group. In this previous article in addition to extensive experimentation also modeling was performed and one of the models was decided to be best based on the fit with experimental data.
(Previous article: Precise transcriptional control of cellular quiescence by BRAVO/WOX5 complex in Arabidopsis roots, Molecular Systems Biology, 2021).

In the now submitted manuscript, the main focus is on modeling, yet it is nowhere made explicit how the current models differ from the previous models and why more modeling is needed, i.e. whether the previous modeling work did not explain all observations, whether there are still alternative possibilities to explain matters that require testing, whether the previous models could not explain spatial patterning, or something in that direction. Thus the rationale behind why this current research was needed is lacking.

Additionally, the authors start with simple models that can be fully analyzed and then when switching from a 1D to a 2D realistic root model all of a sudden change everything:
-two model options are combined
-mass action terms are replaced by saturation functions
-an additional interaction is added The authors have some handwaving explanations for this being better, but it becomes nowhere clear how each of these changes would individually affect the results priorly obtained with the simpler model, how they add up,
whether they are necessary and why they were not investigated in the simpler models. As a consequence there is a large disconnect between the extensively analysied 1D simple models and the subsequent 2D root model.

Although I have great respect and appreciation for the overall work of this research group, based on the above for this particular manuscript I am left with the question whether this paper is more than a collection of left over model results or has true merit as a stand alone paper.

© 2022. Published by The Company of Biologists under the terms of the Creative Commons Attribution License (https://creativecommons.org/licenses/by/4.0/).

Comments for the author

Major concerns were addressed above. Repeated here for completeness:
The articles subject is of a relatively limited scope, it only adresses the narrowing of the BRAVO and WOX5 protein domeins in the Arabidopsis root stem cell niche, which furthermore was previously extensively adressed in another article of the same research group. In this previous article in addition to extensive experimentation also modeling was performed and one of the models was decided to be best based on the fit with experimental data.
(Previous article: Precise transcriptional control of cellular quiescence by BRAVO/WOX5 complex in Arabidopsis roots, Molecular Systems Biology, 2021).
In the now submitted manuscript, the main focus is on modeling, yet it is nowhere made explicit how the current models differ from the previous models and why more modeling is needed, i.e. whether the previous modeling work did not explain all observations, whether there are still alternative possibilities to explain matters that require testing, whether the previous models could not explain spatial patterning, or something in that direction. Thus the rationale behind why this current research was needed is lacking.
Additionally, the authors start with simple models that can be fully analyzed and then when switching from a 1D to a 2D realistic root model all of a sudden change everything:
-two model options are combined
-mass action terms are replaced by saturation functions
-an additional interaction is added The authors have some handwaving explanations for this being better, but it becomes nowhere clear how each of these changes would individually affect the results priorly obtained with the simpler model, how they add up,
whether they are necessary and why they were not investigated in the simpler models. As a consequence there is a large disconnect between the extensively analysied 1D simple models and the subsequent 2D root model.
Although I have great respect and appreciation for the overall work of this research group, based on the above for this particular manuscript I am left with the question whether this paper is more than a collection of left over model results or has true merit as a stand alone paper.

Intermediate points
-line 252: why are different geometries for wildtype and bravo mutants used, how do these geometries differ? would results be different if the same geometries were used?
-line 278-279: the text suggests that the same substances could get different diffusion coefficients when inside different cell types. It is nowhere made clear whether this was actually applied, for which cells and substances and what was the rationale behind this.
Minor points
-Figure 1, colors for QC and CX are too similar, some problem for VI and CSC also I do not see CEI. In legend vascular initials have in brackets (V), should be (VI).
-the authors discuss different diffusion rates, but it is nowhere made clear in the text how diffusion rates differ, e.g. in lines 177-189 it appears that WOX5 is less mobile than X, but how much? and order of magnitude, more, or less? It helps the interpretation to specify this in the text.
-line 189-190 the authors state that the attentuation mechanism also is able to drive BRAVO self-confinement but I find the effect rather limited, so this should be made explicit
-Figure 4 strange panel labelling, A, and B separate, than C for 4 panels, than D and E for separate panels
- line 402-403 common to describe this as mass action
-line 429 such a rectangular function is called a heaviside (step) function
-in section 4.6 the authors use the word settled, which is strange, better to use set/used/applied

© 2022. Published by The Company of Biologists under the terms of the Creative Commons Attribution License (https://creativecommons.org/licenses/by/4.0/).

First revision

Author response to reviewers' comments

Reviewer 1 Advance Summary and Potential Significance to Field:

This paper explores different networks that may confine the expression of two transcription factors, BRAVO and WOX5 within the stem cell niche. It uses a set of experimental observations mostly published in previous papers by the authors, and explores the effect that altering network topology would have on the ability of a WOX5-BRAVO module to restrict the expression of BRAVO.

This work firstly involves the exploration of three models within a one dimensional framework. Although any of these scenarios could regulate patterning, there are restrictions that make them more or less likely. The patterning ability is then explored in a rather beautiful 2D model based on realistic geometries. This model further clarifies the feasibility of immobilisation of BRAVO by WOX5 to self-confine BRAVO. The model does not (and cannot) predict which of these scenarios operates, but provides important insights into what assumptions/paramaters would be necessary to enable these. For example, differences in the relative expression levels of WOX5 and BRAVO support some versions more strongly.

This is an important contribution, and forms an important piece in the puzzle of regulation of the root stem cell niche. One particular area of interest is the proposal of an as yet unidentified protein downstream of WOX5 that regulates BRAVO. The paper provides insight into how such a protein would behave (mobile, and regulated in an opposing manner in bravo and wox5) that identifying such a protein would not be a distant proposition. It also serves as a neat exemplar of the effect that relatively small changes to network structure or diffusion of molecules can have on patterning. As such, I think it would have relevance across different stem cell niches and would be suitable for publication in broad journal such as Development.

Reviewer 1 Comments for the Author:
I am enthusiastic about the paper, but have some minor comments.

Response: We thank the reviewer fur such a positive evaluation of our manuscript.

Title
I think this simply has to have BRAVO and WOX in the title. In my opinion the current title is simply too broad.

Response: The title has been changed to “*BRAVO* self-confined expression through WOX5 in the *Arabidopsis* root stem cell niche”

Abstract
I feel the description of the Root Stem Cell niche is too centred on WOX5 and BRAVO. The introduction is more balanced, but I think abstract doesn't put the work in context in the wider context of stem cell regulation.
Response: The abstract has been modified to (added parts are in green) (lines 15-28 (line numbering referred to the PDF file of the manuscript)):

“In animals and plants, stem cell niches are local microenvironments that are tightly regulated to preserve their unique identity while communicating with adjacent cells that will give rise to specialized cell types. In the primary root of *Arabidopsis thaliana*, the stem cell niche comprises the expression of two transcription factors, BRAVO and WOX5, among others. Intriguingly, BRAVO, a repressor of QC divisions, confines its own gene expression to the niche, as evidenced in its mutant background. Here we propose through mathematical modeling that BRAVO confines its own expression domain to the stem cell niche by attenuating its WOX5-dependent diffusible activator. This negative feedback drives WOX5 action to be spatially restricted as well. The results show that WOX5 diffusion and sequestration by binding to BRAVO is sufficient to drive realistic confined *BRAVO* expression at the stem cell niche. We propose that attenuation of a diffusible activator can be a general mechanism acting at other stem cell niches to spatially confine genetic activity to a small region while at the same time maintain signaling within it and with the surrounding cells.”

© 2022. Published by The Company of Biologists under the terms of the Creative Commons Attribution License (https://creativecommons.org/licenses/by/4.0/).

Results

As all models do, this relies on assumptions based on experimentation. In this case the relevant experiments had all been published and were cited, but I found myself having to go back to the literature to understand the rationale behind them. I found the images in 1B-E unconvincing. The data in the original paper (Betegon-Putze) was very convincing with quantification of the differences in expression pattern. I would have appreciated a better description of the data supporting these assumptions. In particular, I had problems understanding what the evidence for WOX5 being expressed at higher levels than BRAVO. Was this based on the reporters, or on RNASeq? I feel this is important as it is used as a method later to separate between the models.

Response: Figure 1 (and its caption) has been modified to include:
+ new panel E: it depicts the profiles of BRAVO promoter expression extracted from panels 1B (WT) and 1C (bravo- 2 mutant) along the longitudinal axis of the root, integrated along the transversal direction of the longitudinal axis. It shows the expansion of the domain towards the vasculature.
+ new panel F: Quantitative data on spatially integrated pBRAVO:GFP expression in *bravo-2*, *wox5-1* and *bravo- 2 wox5-1* mutants over the WT, which were reported in Betegon-Putze et al. 2021.
+ new panel G: Transcript abundance (reads count) from the literature (Betegon-Putze et al 2021 and Clark et al. 2020) for BRAVO and WOX5 at the QC in the WT.
The picture of pBRAVO:GFP in the *bravo-2 wox5-1* mutant (very similar to that in *wox5-1* mutant) has been removed.

The 1D models were clear but the schematics e.g. 2A show network topology but lack any spatial framework. This might be better served with an additional schematic in showing the spatial movement of components (WOX5).

Response: In each main revised Figures 2, 3, 4 and 5, we have added a panel B with cartoons of each of the 1D models in a spatial framework.

I felt that the attenuation by sequestration model was peculiar. The authors state that in this model WOX5 does not move, but they note that in the experimental data it moves slowly. Given the discrepancy between assumption and observation, I would have expected to have seen more exploration of the parameter space around WOX5 mobility.

Is it essential for the model that WOX5 doesn't move? If so, could the authors explain their rationale behind this model more clearly. The same comment about WOX5 movement is also relevant for the repression model.

Response: We thank the reviewer for pointing this out. Following suggestion by reviewer 2 (point 8), the attenuation model has been removed. We have substituted it by the analysis of the immobilization model with an intermediary molecule Z that diffuses, being WOX5 also mobile (indeed, this is a mixture of the attenuation by sequestration and the immobilization by sequestration models). This model is now introduced to assess whether a mobile Z relaxes the condition of how much WOX5 diffusion is required. New results on the exploration of WOX5 diffusion coefficient values are presented in Figs. 3E,F (supplemented by Fig S4).

Regarding the repression model, we now introduce it with WOX5 that diffuses (lines 299-303) and show that this diffusion is not required to achieve self-confinement (line 306). Results that explore the effect of WOX5 diffusion and Z diffusion are now presented (Fig. 4G supplemented by Figs. S5). The repression model drives self- confinement also when WOX5 diffuses.

Following suggestions by Reviewer 2 (point 2), the new results in Figs. 3E,F and 4G are presented in the form of mapping out those values of WOX5 and Z diffusion coefficients (and of WOX5 diffusion coefficient and binding strength lambda for Fig. 3E) which drive results compatible with experimental observations (in Fig. 1E-G). In supplementary figures S4, S5 we show new data that complement these panels. Specifically, these supplementary figures show the measures extracted from the simulations which are used to compare the simulation results with the experimental data. We refer to answer to point 2 from Reviewer 2 for more detail on how these results are computed.

The 2D modelling in realistic geometries was very elegant, but this part seemed to finish quite abruptly. I understand that the models are useful to see how the patterns of these two reporters

© 2022. Published by The Company of Biologists under the terms of the Creative Commons Attribution License (https://creativecommons.org/licenses/by/4.0/).

shift, but I wonder if it is possible to more closely compare the predicted patterns with experimental data. I'm assuming that the middle panels on Fig 5 are model predictions. Is it possible to present side-by-side experimental data in this way too?

Response: In Figure 1, we have added a new panel E which shows the spatial profile of pBRAVO:GFP in WT and bravo-2 mutant corresponding to the images from real roots in Fig. 1B and 1C. This panel can now be compared to those obtained from simulations in realistic geometries (middle panels of Figure 6).

The results in Figure 6 (former Figure 5), which all are from simulations in realistic geometries, have been revised and changed to a new set of parameter values to increase the resemblance to those from real roots and to enable a clearer connection with the analyses done in the one-dimensional model.

Both Fig 1E and middle panels in Fig 6 show the value of pBRAVO:GFP (from a real root in Fig. 1E and from simulations of the mixed model in Figure 6) that is obtained by integrating pBRAVO:GFP along the transversal direction (in the plane of the image) at each longitudinal position. This is now specified in each caption.

At the end of this document, we provide a summary of changes in Figures.

Reviewer 2 Advance Summary and Potential Significance to Field:

The paper uses computer simulations of mathematical models to address the question of how regulatory interactions between transcription factors can underlie their spatial restriction in a stem cell niche. The focus is on the stem cell niche in the primary root of the plant Arabisopsis thaliana, and the transcription factors WOX5 and BRAVO. This is a well-studied system, but the models discussed here build on existing data (largely from this group) to address an important question that is currently unresolved.

The paper uses a suite of related models to explore the potential contributions of different types of interactions to localisation. Interestingly, models are simulated in highly idealised one-dimensional form and in more realistic data- derived 2D form. The former allows a clear focus on the roles of the different regulatory mechanisms while the latter then allows the consequences of these mechanisms to be studied in the context of the cellular architcture of the root.

The research reported in the paper represents two advances:

1. The modelling shows the sufficiency of a mechanism based on "attenuation of a mobile activator" for localisation. The mechanism fits into a broader theme of spatially-restricted intercellular signalling in plants based on mobile factors (typically transcription factors and micro RNAs) and restricting interactions, but introduces a novel variant (distinct to the proposed mechanism in the shoot apical meristem, for example). This provides an additional principle that can underlie restriction of activities to stem cell niches (which could also apply in animal tissues if taken as an abstract principle).

2. It provides a nice worked example of the simulation of intercellular movement and regulatory interactions in data- derived root tissue geometries.

Both these advances speak specifically to the question of the regulation of the stem cell niche in the Arabisopsis root, and to the broader question of how to understand and model the regulation of stem cell niches generally.

The paper demonstrates the sufficiency of the proposed mechanisms to account for current data on wild type and mutant expression patterns, and maps out (roughly) the spaces of appropriate parameters (i.e. relative weights of different regulatory interactions). There are no new data presented to support the modelling.
Mathematical and numerical details are self-contained in the Methods section and Supplementary Information, and the paper should be accessible to a broad audience.

© 2022. Published by The Company of Biologists under the terms of the Creative Commons Attribution License (https://creativecommons.org/licenses/by/4.0/).

Response: We thank the reviewer for the detailed and positive evaluation of our work.

Reviewer 2 Comments for the Author:

1. I like the combination of rather idealised models followed by simulations in the more realistic root geometries. I think the rationale for the two could be spelled out more clearly, and that this would be helpful for the structure of the paper. It would help if the roles being played by the two modelling approaches were explained (as a reader, I might ask why you don't move immediately to the final models). In tandem with this, I think the principal assumptions that go in to each model could be made clearer. As it stands, the reader has to do some work to pull out the assumptions from the text.

Response: We have modified the end of the Introduction to state the rationale for each of the two types of approaches being used (lines 105-120 (line numbering referred to the PDF file of the manuscript)). In Results, and for each model, we now state the principal assumptions and enumerate them (lines 162-167; 222-223; 276-281; 299-303).

2. Given that (as I see it) the initial models are there to allow a clear exploration of the relative contributions of the different types of regulatory interaction (complex formation, feedback regulation, etc.), I think it would be valuable to be more explicit in mapping out the parameter domains that give results compatible with existing data. Also, the constraints imposed by these data need to made more explicit (I infer from the paper that we should expect WOX5 promoter activity [in some sense] to be around 5 times higher than BRAVO promoter activity, but this isn't made very clear; I'm less clear about the numerical data on the extent of expression domain changes in the mutants). Can these be made clear and then the appropriate parameter domains be mapped out (e.g. in a lambda-alpha plane for the "immobilisation by sequestration" model)?

Response: We agree that in our original manuscript the constraints imposed by existing empirical data were dispersed across the text and were not made sufficiently clear. To address this criticism:

1) We have included in Figure 1 reported quantitative data on three different aspects that we use to compare with our simulation results: (new panel E) the profile of BRAVO promoter expression extracted from figures 1B and 1C along the longitudinal axis of the root (i.e. in the WT and in the bravo-2 mutant); (new panel F) Quantitative data on the ratio of spatially integrated pBRAVO:GFP expression in *bravo-2*, *wox5-1* and *bravo-2 wox5-1* mutants over that in the WT, which was reported in Betegon-Putze et al. 2021; and (new panel G) Transcript abundance (read counts) for BRAVO and WOX5 at the QC in the WT (from Betegon-Putze et al 2021 and Clark et al. 2020).

2) In the first section of Results, we have included a paragraph that enumerates and defines each of the observables (measures) we define to compare the model results with empirical data, and states which is the range of their values according to empirical data (lines 187-199, 240-249). In our original manuscript we assessed three different observables when comparing to experimental data. In the revised manuscript we assess these same three ones and add a fourth additional one. These observables are: (i) The adimensional measure of the expansion of BRAVO promoter expression in the bravo mutant, as defined in the original manuscript (xi); (iii) The increase of BRAVO promoter activity at the QC in the bravo mutant compared with the WT; this quantity was mentioned and used in the original manuscript but was not given any name. Now we name it as R_B; (iii) Ratio of BRAVO promoter expression at VI over that of WOX5 at the QC, both in the WT. In the original manuscript this ratio was named as R. Now we named it as R_{BW}, to distinguish it from the other observables, and to emphasize that it compares BRAVO and WOX5 expressions. Notice these three observables are related to the experimental data provided in new panels Fig 1E-G; (iv) Ratio of WOX5 promoter activity at the QC in the bravo mutant over that in the WT. This ratio was not defined in the original manuscript. We name it R_W and is introduced and used only when WOX5 self-repression is included in the 1D models (lines 240-249). This observable can be compared with data of pWOX5:GFP in the bravo-2 mutant in Betegon et al. 2021.

3) We have included new results on multiple parameter space explorations to map out the parameter regions where 1D simulation results are compatible with experimental data, according to the observables defined. We have explored 4 different parameter spaces: (i) binding strength (lambda)- BRAVO production rate (alpha_L,alpha); (ii) WOX5 diffusion-lambda; (iii) WOX5 diffusion-

© 2022. Published by The Company of Biologists under the terms of the Creative Commons Attribution License (https://creativecommons.org/licenses/by/4.0/).

Z diffusion; and (iv) Z production rate (beta) - BRAVO-mediated repression (c). We have replaced the panels on how xi and R observables change with a single parameter (e.g. lambda) by these new parameter space explorations.
Revised main figures show several of these parameter domains for each of the 1D models being studied: Fig 2F, 2G, 2I, 2J; Fig 3E, 3F; Fig 4G, 4H; Fig 5D, 5E, 5F. In revised and new supplementary figures S1-S7 we disclose each of these parameter domains in several colormaps that each depict the value of an observable across the parameter space. These colormaps are shown to see how the parameter domains are constructed and to better understand how each observable depends on each parameter.

In Methods, we have also rewritten the section where observables were defined to clarify how many we use and to state their mathematical definitions (subsection M6 of Methods). We have also included the details of how these computations were performed (in subsection M9, lines 798-804).

3. Are there any data available on WOX5 protein distributions (not pWOX5::GFP)? The models make predictions about the spatial distribution of WOX5 protein (when it is assumed to move), and it would be really helpful to be able to compare to these.

Response: Yes, in papers Berckmans et al. Plant Physiol 2020, and in Clark et al. PNAS 2020, WOX5 protein fused to GFP is seen in the QC and in VI and CSC but not much further away. This is now stated in the manuscript (lines 210-212, lines 384-387).

4. Some of the models depend quite sensitively on the balance between the concentrations of WOX5 protein, BRAVO protein, and protein complexes. I appreciate that complex concentrations have been ommited for clarity, but I find it difficult to really get a deep feel for the operation of some of the models without having these results. They could be in the Supplement, but I think they're an important part of the models (as alluded to in the paper at various points) and should be presented.

Response: We have included panels showing an amount proportional to the complex (i.e. B*W, in violet). Our simulations are done with the simplified models presented in Methods which do not have the complex as a variable. Hence, we do not simulate the complex per se. Yet, since the amount of complex is proportional to unbound BRAVO (B) times unbound WOX5(W), we used it to depict the complex profile. We have included these results in all revised Supp. Figs. which provide data from models with sequestration: Figs S1, S2, S3, S4 and S7.

5. The idealised models use linear production terms, while the simulations on root geometries use nonlinear terms. I appreciate the value of the extra simplicity (reduced parameters) of the idealised models, but it would be interesting to know how the use of similar nonlinear production terms would affect the conclusions about appropriate parameter domains (since the balance between production [nonlinear; saturating] and binding [mass action] would be different at different concentrations).

Response: In the revised manuscript we have studied all the 1D models with nonlinear saturating functions, as requested, for cooperativity n=1 and n=3. Our results show now the parameter domains were these simulations drive results compatible with experimental data. These analyses have been performed in the parameter spaces defined in response to point 2. The parameter domains as well as the value of each observable are presented. The stationary spatial profiles of all variables for the WT and the bravo mutant for a default set of parameter values are also depicted. For each model, the results are shown in the following figures:

- For the immobilization by sequestration model, new Fig. S2A,B contains the results with nonlinear functions. These are to be compared with the new results in Fig 2F-G and Fig. S1, which show the analogous but in the linear case.
- For the Repression model, new Fig. S6 contains all results with nonlinear functions. These are to be compared with revised Fig. S5, which contains all results for linear functions.

Notice that parameters related to the production of BRAVO and of Z (alpha and beta) have different meanings in the linear case than in the nonlinear one. Hence, we chose to use a slightly

© 2022. Published by The Company of Biologists under the terms of the Creative Commons Attribution License (https://creativecommons.org/licenses/by/4.0/).

different name: alpha_L (beta_L) for the linear case and alpha (beta) for the nonlinear case. In the linear case these parameters are the production rate per unit of concentration of the activating specie, whereas for the nonlinear case these parameters are the concentration produced per unit of time in the saturated regime. Hence, direct comparison between the linear and the nonlinear cases of the parameter domains involving these parameters cannot be made.

Regarding the parameter domains, the most significant difference we find when using nonlinear functions compared to the linear ones is that nonlinear terms facilitate the feasibility of the repression model, since the parameter domain in the beta-c plane is much larger than in the beta_L-c plane for the repression model. This is stated in lines 313-315.

In Methods, the 1D models were originally defined using linear terms. To include the description of nonlinear functions for production terms we have termed the production terms in the dynamical equations as functions, whose expression we specify for the linear case and for the non-linear case.

6. The discussion of the effect of a general squestrator S is rather cursory (lines 159-163). I'd like to know more, especially since it is known that there are other binding factors in the stem cell niche. What are the constraints? How much complex of B and W with S can be permitted compared to B with W? It is clear from the results that some S is permissible, but I can't get a good feel for how much. Since it is assumed that [BRAVO] << [WOX5], I presume things hinge on the relative concentrations of BRAVO and S. Is this right, and can it be determined from the simulations?

Response: The referee is correct and the relative concentration of BRAVO and S is crucial. We have modified this paragraph (lines 222-231) and have added several new results on Fig. S3 to clarify it. The new results depict amounts proportional to the complexes, compare the profiles with and without sequestrator and provide parameter space explorations.

7. There are problems with Eqs. (34) and (35), and the expression for B on line 768. The expression for B should be B = \frac{\alpha W}{\lambda W + d_B}

and then

d_W' = d_W + \frac{\lambda \alpha W}{\lambda W + d_B}.

B is an increasing function of W, and the effective degradation rate of W is an _increasing_ function of W. These need to be corrected, but I'm confident that these are typos and that the simulations use the correct forms.

Response: The reviewer is correct and this typo, which was not in the simulations, is now corrected in the SI text. These equations involved the parameter formerly defined as alpha. Now it is renamed to alpha_L and in SI text we explicitly indicate that the model refers to the linear case.

8. Since the "attenuation by sequestration" model doesn't fit with data, is it really worth including it in the main text? I'd be inclined to restrict it to the Supplement, at best. As it is, it detracts from the logical flow of the main text.

Response: the “attenuation by sequestration” has been removed from the manuscript: the section, original figure 3 and original Supp Fig. S3 have been all removed. To address criticisms by reviewer 3, we decided to study the immobilization by sequestration model with an intermediary Z that diffuses (lines 265-286). This case is a mixture of the original immobilization by sequestration model and the original attenuation by sequestration model. New Fig. 3 contains these new results (supplemented by new Fig. S4).

9. It is stated that the results of transcriptomics in bravo and wox5 mutants yield potential candidates for Z. Can you say anything more than that? It would be really valuable to know some of the potential candidates. This would give a way of selecting between alternatives / validating the models. Are there any candidates with known expression patterns that match that expected of Z from the simulations?

© 2022. Published by The Company of Biologists under the terms of the Creative Commons Attribution License (https://creativecommons.org/licenses/by/4.0/).

Response: We have added a new Fig S15 that makes a colormap representation of the transcript abundance (read counts) of potential candidates across the different cell types of the WT root SCN. For this visual representation, we have used the realistic geometry of the WT SCN used for simulations. The transcript abundance plotted is extracted from RNAseq data (read counts) of WT Arabidopsis from Clark et al. Nat Comm (2019). The list of genes whose abundance is shown is extracted from those genes that show significant antagonistic regulation by BRAVO and WOX5, as reported in Betegon-Putze Mol Sys Biol (2021). These results are commented in the Discussion (lines 414-429).

10. On line 293, you state that pWOX5 activity should decrease in the bravo mutant. Is this observable? Are there any data to compare to?

Response: Yes. We have reported this in Vilarrasa et al Dev Cell 2014 and Betegon-Putze et al Mol Sys Biol 2021. To clarify this, in the Introduction (lines 86-92) we now explicitly indicate that BRAVO promotes WOX5 expression and state the mechanism behind it, a mechanism we reported in Betegon-Putze et al Mol Sys Biol 2021. This mechanism relies on WOX5 self-repression and BRAVO-WOX5 binding.
In addition, we have added a new measure in our simulations, the observable R_W (as indicated in answer to point 2), to compare it with the experimental data in Betegon-Putze et al Mol Sys Biol 2021. This observable measures the change of WOX5 promoter activity in the bravo mutant with respect to the WT.

11. The full root models have basal BRAVO production in the vasculature (in line with data) and WOX5 auto- repression (inspired by data). I think these should really be in the idealised models, too (since they're directly reflecting the data).

Response: We have added both basal production in the vasculature and WOX5 self-repression as follows.

As indicated in the previous answer, the results in Betegon-Putze et al Mol Sys Biol 2021 proposed that BRAVO promotes WOX5 through protein binding with WOX5 and WOX5 self-repression (this self-repression, or negative feedback, is supported by the increased expression of WOX5 promoter in wox5-1 mutant compared to the WT). We have used this rationale to introduce WOX5 self-repression in the immobilization by sequestration mechanism (lines 233-249). The results are depicted in new panels Fig 2H-J and complemented by new Fig. S2C.

Because of this rationale, for the Repression model we have included WOX5 self-repression together with WOX5 and BRAVO protein binding, since both of them are required to account for the role of BRAVO on WOX5 promoter. This constitutes the mixed model in 1D (lines 317-328). The new results are shown in New Fig 5 and new Fig. S7 A,B.

Basal production in the vasculature has been added in the immobilization model with an intermediary and in the mixed model, and the results are shown in Fig. S4B and Fig. S7C respectively.

We have added in Methods the description of the Mixed model in 1D, as well as the WOX5 self-repression and basal production in 1D.

12. On line 295, it is claimed that the parameters are "biologically reasonable". Can you expand on this a bit to justify the claim? Is GFP really likely to be less diffisible than the TFs? How easily can you interpret movement parameters when the interior of the cell is taken as homogeneous (no vacuole)?

Response: We agree that the use of “biologically reasonable” is not the best choice. Hence, this sentence has been modified (lines 384-389). We focused on WOX5 diffusion coefficients and WOX5 degradations which could drive a WOX5 protein distribution like that found in experiments, which show WOX5 protein bound to GFP (Berckmans et al 2020, Clark et al 2020). Regarding GFP, we used GFP diffusions and degradations that can drive a pWOX5:GFP expression similar to that in experiments, which is mostly concentrated at the QC and not as much further away (lines 364-366). This results in smaller GFP diffusion coefficients. In addition, we changed the parameter values

© 2022. Published by The Company of Biologists under the terms of the Creative Commons Attribution License (https://creativecommons.org/licenses/by/4.0/).

from the original manuscript to make them more comparable to the 1D models, while fitting the conditions just stated.

13. On line 298, you discuss conditions for the restriction of pWOX5 activity mostly to the QC, VI and CSC. However, the model claims to impose pWOX5 activity _only_ in the QC (by \gamma_{QC}(r)). I'm confused - you can't have expression outside the QC in the model...

Response: The reviewer is right. The sentence on the conditions for restrictions was incorrect and stated pWOX5 while it should state WOX5. This sentence has been modified when addressing the previous point 12 (lines 384- 388).

14. One feature that has emerged from studies of morphogen gradients in animal tissues is the role that complex formation and feedbacks can make to buffering morphogen range against variation in source strength (e.g. citations [35] and [36] for BMP, and Irons et al. (2010). Dev. Bio. 342, 180–193 for Hedgehog). Given the broad parallels with the models described here, it would be really interesting to know if the proposed mechanisms for restricting the expression of SCN factors can also contribute to buffering against source strength. It would be well worth seeing the effect of changes in the WOX5 production rate (\gamma_{QC}), and how the interactions with BRAVO might buffer this. This has been a significant area of study for animal morphogen gradients, and the question is equally pertinent for plant tissues. Specifically, the QC is specified at least in part by hormone profiles, and some quantitative variability in these is to be expected (even if the position of, say, an auxin maximum is relatively stable). A robust mechanism for maintaining a stable SCN will surely require some buffering against quantitative fluctuations.

Response: Following the reviewer's suggestion, we have analyzed at which spatial position the BRAVO promoter activity reaches a defined value (L2) and how this position changes when the production of WOX5 is reduced at half. We have computed it in the immobilization model and compared it to the case when BRAVO is absent (and hence no immobilization mechanism is acting). In this latter case the gradient is exponential. If the immobilization mechanism provides robustness we should expect that the change in spatial position is smaller in the immobilization model than in the exponential case. This is indeed the case. However, when this expansion is divided by the distance at which L2 value is reached, then both cases drive similar results. Hence, we do not find a clear signature for a robust response. We only find it when BRAVO is much more strongly expressed, but this corresponds to a situation which is not consistent with other experimental data (e.g. with Fig. 1G). These new results have been included in new Fig. S16. These negative results are discussed in the Discussion (lines 473- 476).

Minor comments:

- the diffusion rates in the figure legends are wrong at some points: D_W in Fig. 2, D_X in Fig. 3 and D_Z in Fig. 4. I believe they should be 0.01, not 4.

Response: The correct values that correspond to the simulation results are 4. The values of the Table have been corrected.

- the formatting of the \lambdas in the legend to Fig. 7 has gone awry.

Response: We have corrected this.

- on line 202, you say "reducing the production of its mobile activator". I think it is actually reducing the diffusion of the mobile activator, not its production.

Response: The sentence was correct and has not been changed. The effect of BRAVO binding to WOX5, makes that there is less WOX5 protein (unbound) available to activate Z, which is mobile. Hence, BRAVO indirectly drives less production of Z.

- you use different cellular architectures for the wild type and bravo mutant roots. Are there data to show that the mutant has a clearly and reproducibly distinct geometry to the wild type? If so, can you summarise the key differences? Does it make much difference to the outcome of the study?

© 2022. Published by The Company of Biologists under the terms of the Creative Commons Attribution License (https://creativecommons.org/licenses/by/4.0/).

Response: Different geometries were used to make it more realistic albeit they are very similar, being only different by the presence of divided cells at the QC in the *bravo* mutant (Vilarrasa et al. Dev Cell 2014). Due to the similarities between the geometries, results do not depend significantly on which geometry is used. To show this, we have added a new Fig. S13, which makes the same computation as Fig.6A but using the same geometry for the WT and for the bravo mutant (the geometry being used is that of the WT). Results in 6A and in Fig. S13 are very similar. This is stated in lines 392-394.

- since all the figures / conclusions depend only on steady state distributions, the discussion of fast complex dynamics is a bit distracting. It is only being used to motivate the reduction of the models to lower numbers of variables, but then the reduced equations are not really being used to infer much. Is it worth reducing the prominence of this, or re-wording somehow?

Response: This has been mostly removed from Methods (lines 533-540), but maintained in SI text.

- the simulations of the models require the specification of boundary conditions, but I can't find any mention of these. Please include them.

Response: We apologize this was missing for the 1D model analysis and we did not notice. We have included them (lines 794-796; for the parameter space exploration, boundary conditions are defined in lines 798-804).

- the title could be changed to be a bit more informative to the (potential) reader.
Response: The title has been changed to "BRAVO self-confined expression through WOX5 in the *Arabidopsis* root stem cell niche"

At the end of this document, we provide a summary of changes in Figures.

Reviewer 3 Advance Summary and Potential Significance to Field:

The articles subject is of a relatively limited scope, it only adressesthe narrowing of the BRAVO and WOX5 protein domeins in the Arabidopsis rootstem cell niche, which furthermore was previously extensively adressed in another article of the same research group. In this previous article in addition to extensive experimentation also modeling was performed and oneof the models was decided to be best based on the fit with experimental data. (Previous article: Precise transcriptional control of cellular quiescence by BRAVO/WOX5 complex in Arabidopsis roots, Molecular Systems Biology, 2021).

In the now submitted manuscript, the main focus is on modeling, yet it is nowhere made explicit how the current models differ from the previous models and why more modeling is needed, i.e. whether the previous modelingwork did not explain all observations, whether there are still alternative possibilities to explain matters that require testing, whether the previous models could not explain spatial patterning, or something in that direction. Thus the rationale behind why this current research was needed is lacking.

Additionally, the authors start with simple models that can be fully analyzed and then when switching from a 1D to a 2D realistic root model all of a sudden change everything:

-two model options are combined
-mass action terms are replaced by saturation functions
-an additional interaction is added

The authors have some handwaving explanations for this being better, but it becomes nowhere clear how each of these changes would individually affect the results priorly obtained with the simpler model, how they add up, whether they are necessary and why they were not investigated in the simpler models. As a consequence there is a large disconnect between the extensively analysied 1D simple models and the subsequent 2D root model.

© 2022. Published by The Company of Biologists under the terms of the Creative Commons Attribution License (https://creativecommons.org/licenses/by/4.0/).

Although I have great respect and appreciation for the overall work of this research group, based on the above for this particular manuscript I am left with the question whether this paper is more than a collection of left over model results or has true merit as a stand alone paper.

Response: We thank the reviewer for pointing out weaknesses and unclear aspects of our manuscript, which we have now thoroughly addressed. Addressing them has strongly improved our manuscript.
The Specific answers are detailed below, in Comments to the Author:

Reviewer 3 Comments for the Author:

Major concerns were addressed above. Repeated here for completeness: The articles subject is of a relatively limited scope, it only adresses the narrowing of the BRAVO and WOX5 protein domeins in the Arabidopsis root stem cell niche, which furthermore was previously extensively adressed in another article of the same research group. In this previous article in addition to extensive experimentation also modeling was performed and one of the models was decided to be best based on the fit with experimental data. (Previous article: Precise transcriptional control of cellular quiescence by BRAVO/WOX5 complex in Arabidopsis roots, Molecular Systems Biology, 2021).

In the now submitted manuscript, the main focus is on modeling, yet it is nowhere made explicit how the current models differ from the previous models and why more modeling is needed, i.e. whether the previous modeling work did not explain all observations, whether there are still alternative possibilities to explain matters that require testing, whether the previous models could not explain spatial patterning, or something in that direction. Thus the rationale behind why this current research was needed is lacking.

Response:
In the final part of the Introduction we now describe in more detail which regulations where inferred from the previous study. We then indicate which aspects were not covered and prompt for the current investigation. In short, the previous study and modeling efforts did not evaluate any spatial pattern, while this manuscript does. These changes are found in lines 86-120 (line numbering referred to the PDF file of the manuscript).

Additionally, the authors start with simple models that can be fully analyzed and then when switching from a 1D to a 2D realistic root model all of a sudden change everything:
-two model options are combined
-mass action terms are replaced by saturation functions
-an additional interaction is added

The authors have some handwaving explanations for this being better, but it becomes nowhere clear how each of these changes would individually affect the results priorly obtained with the simpler model, how they add up, whether they are necessary and why they were not investigated in the simpler models. As a consequence there is a large disconnect between the extensively analysied 1D simple models and the subsequent 2D root model.

Although I have great respect and appreciation for the overall work of this research group, based on the above for this particular manuscript I am left with the question whether this paper is more than a collection of left over model results or has true merit as a stand alone paper.

Response: We appreciate these criticisms which prompted us to think about the structure and rationale of the manuscript and the missing connections between the results. We have modified the rationale and have added multiple new results, to make the changes step by step. In the revised manuscript we have added:

i) The study in 1D of the mixed model, which combines the two models (lines 317-328), with results in New Figure 5 and New Fig. S7.
ii) The study of 1D models for saturating functions and not only for linear terms (see Answer to point 5 from reviewer 2).
iii) The additional interaction (wox5 self-repression) in 1D models (see Answer to point 11 from reviewer 2).

© 2022. Published by The Company of Biologists under the terms of the Creative Commons Attribution License (https://creativecommons.org/licenses/by/4.0/).

The rationale of the manuscript has been modified to make one change at a time:
First, we study the Immobilization by sequestration model in 1D with mass action terms (Figure 2A-G, Revised Fig. S1), as originally conceived, and then with saturating functions for production (lines 201-203, New Fig. S2 A-B). In this model (with nonlinear saturating functions) we then include WOX5 self-repression which, together with BRAVO binding to WOX5, can account for BRAVO promoting WOX5 expression (lines 233-249). The results are shown in new panels Fig 2H-J and Fig S2C.
Then the immobilization by sequestration model, with WOX5 self-repression, is extended to the case when WOX5 activates BRAVO through an intermediate molecule Z, which diffuses. This enables to address whereas BRAVO self-confinement can arise at lower WOX5 diffusion coefficients if Z is mobile. This new section (lines 265-286) is entitled “A mobile intermediary can facilitate BRAVO self-confinement”. This substitutes the attenuation by sequestration model which has been removed, following suggestion by reviewer 2. Results are presented in New Fig 3 and New Fig S4, and are all made with saturating functions.
We then asses the repression model, as originally conceived with linear productions, and with saturating functions (new Fig.4H and New Fig S6, lines 309-311). WOX5 diffusion is also included.
In the repression model we then include WOX5 self-inhibition and BRAVO-WOX5 protein binding to account for BRAVO promoting WOX5 expression. This is the mixed model in 1D, which combines the repression and the Immobilization by sequestration model (lines 317-328). The results are in New Figure 5 and New Fig. S7. We then study the mixed model in the realistic root geometry to overcome the limitations of the 1D geometry.
Accordingly, the main text has been extensively revised to accommodate to this change in the rationale, and to explain the new results obtained from the new analyses.
In Methods, we have defined all these 1D models and have used a very similar notation for the Mixed model in the realistic geometry as in the 1D framework to facilitate comparison (line 734-760).

The original Figure 5 exhibited results in the realistic geometries for different cases. These cases can now be readily connected to the 1D models. This Figure has been recomputed for a new set of parameter values and constitutes Figure 6. The new parameter set has been chosen to improve the resemblance of the simulation results with experimental data and to better connect with the parameter values used for 1D models.

While performing these revisions, we have obtained that either sequestration or repression can help confine the BRAVO promoter expression domain when there is a molecule Z that diffuses (lines 395-399). Figure 6 and new Fig. S14, which is analogous to Fig.6 but for another parameter set, show these results.

Intermediate points

-line 252: why are different geometries for wildtype and bravo mutants used, how do these geometries differ? would results be different if the same geometries were used?

Response (The response to reviewer 2 on this aspect is copied here): Different geometries were used to make it more realistic albeit they are very similar, being only different by the presence of divided cells at the QC in the *bravo* mutant (Vilarrasa et al. Dev Cell 2014). Due to the similarities between the geometries, results do not depend significantly on which geometry is used. To show this, we have added a new Fig. S13, which makes the same computation as Fig.6A but using the same geometry for the WT and for the bravo mutant (the geometry being used is that of the WT). Results in 6A and in Fig. S13 are very similar. This is stated in lines 392-393.

-line 278-279: the text suggests that the same substances could get different diffusion coefficients when inside different cell types. It is nowhere made clear whether this was actually applied, for which cells and substances and what was the rationale behind this.

Response: This is now clarified (lines 373-374): “we set the diffusion coefficient of each molecular species to be the same in all cell types.”

In Methods, we also added this clarification (lines 763-764).

© 2022. Published by The Company of Biologists under the terms of the Creative Commons Attribution License (https://creativecommons.org/licenses/by/4.0/).

Minor points

-Figure 1, colors for QC and CX are too similar, some problem for VI and CSC also I do not see CEI. In legend vascular initials have in brackets (V), should be (VI).

Response: Colors have been changed. CEI and Pericycle have been added. VI has been corrected in the legend.

-the authors discuss different diffusion rates, but it is nowhere made clear in the text how diffusion rates differ, e.g. in lines 177-189 it appears that WOX5 is less mobile than X, but how much? and order of magnitude, more, or less? It helps the interpretation to specify this in the text.

Response: In main Figures and Supplementary Figures several panels of parameter space exploration have been added, being one of them the space of WOX5 and Z diffusion coefficients. In these panels, we have added a star to denote the default value of WOX5 and of Z diffusion coefficients used to depict the profile (Fig. 3F and 5D). In Fig. 3D the values of WOX5 and Z diffusion coefficients have been also added inside the panel.

-line 189-190 the authors state that the attentuation mechanism also is able to drive BRAVO self-confinement but I find the effect rather limited, so this should be made explicit

Response: Following suggestions from reviewer 2 (point 8), the attenuation model has been removed from the manuscript: the section, original figure 3 and original Supp Fig. S3 have been all removed.

-Figure 4 strange panel labelling, A, and B separate, than C for 4 panels, than D and E for separate panels

Response: In main figures we have now lettered each panel.

- line 402-403 common to describe this as mass action

Response: We have now indicated as such (line 533)

-line 429 such a rectangular function is called a heaviside (step) function

Response: We have now indicated as such (line 570). We realized we had to introduce it earlier, already for BRAVO (since BRAVO is only produced at the QC and towards the vasculature). Hence, we have introduced it in line 561.

-in section 4.6 the authors use the word settled, which is strange, better to use set/used/applied

Response: It has been changed to "set".

To all reviewers, we provide a summary of changes in Figures:

Figure 1: has been revised to clarify it and to provide quantitative data (reported in publications) from Arabidopsis roots (new panels E,F,G). The revised manuscript contains new results on the parameter domains (for multiple parameter spaces) where simulations reproduce these data.

Figure 2: new results have been added, which constitute new panels F, G, I and J. These new results concern parameter space explorations in two different spaces, for the Immobilization by sequestration model and when WOX5 self-repression is added. Results for linear and nonlinear productions are shown. Results on valid parameter domains substitute former analyses on single parameter variation. Panels B and H with the models' cartoon in the one dimensional geometry have also been added.

Figure 3: it is entirely new. It is devoted to the study of the Immobilization by sequestration model when an intermediary factor, which is mobile, is added. This model helps the transition towards the Mixed model.

© 2022. Published by The Company of Biologists under the terms of the Creative Commons Attribution License (https://creativecommons.org/licenses/by/4.0/).

Figure 4: it contains data from former Figure 3 on the Repression model and new results (panels G, H) on explorations of parameter spaces of this model when WOX5 diffusion is included and for linear and nonlinear production functions. Results on valid parameter domains substitute former analyses on single parameter variation. Panel B the model's cartoon in the one dimensional geometry has also been added.

Figure 5: it is entirely new. It is devoted to the study of the Mixed model in one dimension. The Mixed model combines the models in Figs 3 and 4.

Figure 6: it is devoted to the Mixed model in realistic root geometries. It corresponds to former Figure 5 but re- computed for a new set of parameter values. This new set has been chosen to increase the resemblance of the model results to the spatial expression profiles now shown in Fig.1 E, and to better establish the connection with one dimensional models in Figs 2-5.

Regarding Supplementary Figures, we have added 8 new Supplementary figures and have revised 5 original ones.

Fig. S1: Results in A are new and substitute analyses on one single parameter by parameter space explorations. It is devoted to the Immobilization by sequestration model with linear production in one dimension (1D). Spatial profiles of BRAVO-WOX5 complex have been added in the other panels.

Fig. S2: it is entirely new. It shows parameter space exploration of the Immobilization by sequestration model in 1D for two non-linear regulatory functions and when WOX5 self-repression is added.

Fig. S3 (former Fig. S2): Spatial profiles of complexes have been added. Results in C on parameter space explorations are new and substitute former analyses on single parameter variation.

Fig. S4: it is entirely new. It is devoted to the analysis of the Immobilization by sequestration model with intermediary with and without basal BRAVO production, in 1D.

Fig. S5 (former Fig. S4): Panel A is new and contains parameter exploration for the Repression model with linear functions in 1D, and substitute previous analyses of single parameter variation.

Fig. S6: it is entirely new. It evaluates the Repression model in 1D for two nonlinear production functions.

Fig. S7: it is entirely new. It evaluates the Mixed model in 1D for two nonlinear cases and with basal production of BRAVO.

Figs S8-10 have not been changed and correspond to original Figs S5-S7.

Fig. S11: it is former Fig. S8 but re-computed for the new set of parameter values of WOX5 in Fig.6. Panels on the bravo mutant case were redundant and have been removed.

Fig. S12: it is former Fig. S9 but re-computed for the new set of parameter values of Fig.6. The spatial profile of pWOX5:GFP along the longitudinal axis has been added.

Fig. S13: it is entirely new. It performs the same computation as Fig. 6A, with the same parameter values, but using the same root geometry for the WT and for the bravo mutant.

Fig. S14: it is new. It is analogous to Fig. 6 but for another set of parameter values.

Fig. S15: it is new. It depicts published data on transcript read counts in the stem cell niche to identify candidates for Z in our models.

Fig. S16: it is new and is devoted to the study of robustness to perturbations in WOX5 level in the Immobilization by sequestration model in 1D.

© 2022. Published by The Company of Biologists under the terms of the Creative Commons Attribution License (https://creativecommons.org/licenses/by/4.0/).

The main text has accordingly been adapted (main changes highlighted in green). We have followed Development guidelines for manuscript extension and formats.

Second decision letter

MS ID#: DEVELOP/2022/200510

MS TITLE: *BRAVO* self-confined expression through WOX5 in the *Arabidopsis* root stem-cell niche

AUTHORS: Josep Mercadal, Isabel Betegón-Putze, Nadja Bosch, Ana I. Caño-Delgado and Marta Ibañes
ARTICLE TYPE: Research Article

I am happy to tell you that your manuscript has been accepted for publication in Development, pending our standard ethics checks.

Reviewer 1

Advance summary and potential significance to field

I was previously enthusiastic about this paper, and I continue to be so. I previously outlined my thoughts on the advances of this paper, and they have not changed.

Comments for the author

I only had minor points, and they have all been resolved.
Abstract and Title These were small things, but I feel that the revised versions better describe the content of the paper.
Assumptions and rationale behind them The authors have made a good effort to explain and provide reasoning behind their assumptions. In particular, I was concerned about how they showed the comparitive levels of expression between WOX and BRAVO, I feel that read count on RNASeq is a reliable method.
WOX5 movement My previous concerns have been explored, and in my non-technical viewpoint this section looks much better.
The comparison between experimental and model prediction is much clearer.

Reviewer 2

Advance summary and potential significance to field

I am happy with the revisions that the authors have made to the manuscript.

Comments for the author

No further comments

Reviewer 3

Advance summary and potential significance to field

See first review report

Comments for the author

© 2022. Published by The Company of Biologists under the terms of the Creative Commons Attribution License (https://creativecommons.org/licenses/by/4.0/).

The authors have substantially improved the manuscript, adding a rationale for the need of the current study in the introduction and using a more incremental model buildup in the 1D models to eventually lead to the 2D anatomical model.

I have two remaining major issues
1) The article only provides an explanation for the self-confimenemt of BRAVO given a certain expression domain of WOX5 and given that WOX5 can only induce BRAVO in the QC and shootward direction. How WOX5 expression is determined and what limits the potential of WOX5 to induce BRAVO spatially remain open questions, among with many others. I would recommend the authors write something on the limitations of their study and the many remaining questions in the field of QC/SCN patterning.
2)Paragraph starting at line 478: Here the authors claim that most other models do not consider diffusion of molecules within cells and that they are among the first to do so. This is simply a false statement, all the root tip models by the Grieneisen group, both on rectangular and realistic topologies, as well as all the root tip models by the Ten Tusscher lab, again both on rectangular and realistic topologies, include diffusion of hormones within cells and within cell walls.

Other remaining less major comments:
1) line 132: it seems to me that the data show that the FAILURE of BRAVO self-confinement requires WOX5, so perhaps better to write "involves WOX5" rather than "requires WOX5"
2) line 187 and below on observables:
- eta can only define spatial expansion relative to a threshold expression level still counting as within the expression domain, this is explained in the methods but it would help to also briefly mention this here
- simlarly Rh only makes sense if it is defined where this expression is measured, again explained in methods, but would help to briefly mention here
-is it not more logical to define for Rh a lower level of e.g. minimal 2-fold upregalation rather than an upper level of up to 2-fold increase?
-last sentence hard to read reformulate "if the ratio between BRAVO VI and WOX5 QCe expression"
3) line 209/201 replace "expands but gets dimmer" with "spatially expands while absolute levels decline".
Also the formulation "The results show" is a bit confusing, at first it seems as if this sentence refers to the previous ones. Maybe better to write "The results also show" or something along those lines

4)line 210-212: It is argued that WOX5 should have limited diffusion based on experimental observations. However, if indeed WOX5 is sequestered by binding to BRAVO and other proteins this is kind of circular reasoning as then the observed mobility of WOX5 is a product not only of its diffusion but also this sequestration. Please clarify.
5)line 225: I think it should read "If instead S production"?
6)line 305-328: I would think that even if WOX5 is completely immobile, still it would be sequestered by the BRAVO it induces in the QC itself, so even in the repression mechanism without WOX5 diffusion there would be a combination of sequestration and repression. If so, this would be good to point out in the paragraph running from line 305 to 315.
Therefore, I think that the mixed model that incorporates the negative feedbackof WOX5 does not so much put back in sequestration with the repression, but rathe adds a certain effect of this sequestration. Please clarify.

© 2022. Published by The Company of Biologists under the terms of the Creative Commons Attribution License (https://creativecommons.org/licenses/by/4.0/).
